# A ribozyme ligase that requires a 3′ terminal phosphate on its RNA substrate

**DOI:** 10.1038/s41467-026-74622-8

**Published:** 2026-07-13

**Authors:** Annyesha Biswas, Zoe Weiss, Jack W. Szostak, Saurja DasGupta

**Affiliations:** 1 Department of Chemistry and Biochemistry, University of Notre Dame, Notre Dame, IN USA; 2 Harvard/Massachusetts Institute of Technology MD-PhD Program, Harvard Medical School, Boston, MA USA; 3 Howard Hughes Medical Institute, The University of Chicago, Chicago, IL USA; 4 Department of Chemistry, The University of Chicago, Chicago, IL USA; 5 Department of Biological Sciences, University of Notre Dame, Notre Dame, IN USA

**Keywords:** RNA, Origin of life

## Abstract

Ribozymes likely played essential roles in catalyzing metabolic processes and facilitating genome replication in primordial RNA-based life. In vitro evolution has allowed us to expand the biochemical capabilities of RNA, especially new ribozyme chemistries. Here, we report the serendipitous discovery of ribozyme ligases that catalyze the attack of the 2′-hydroxyl group of an RNA substrate on its own 5′-triphosphate group, but only when the substrate possesses a 3′-phosphate vicinal to its nucleophilic 2′-hydroxyl group. The ligases′ requirement for a 3′-phosphate group on its substrate resembles enzymatic mechanisms found in protein-based RNA repair pathways. We propose that ribozyme-catalyzed ligation of 3′-phosphorylated RNA could have provided pathways for RNA repair in primordial cells. We demonstrate that these ribozymes ligate specifically to 3′-phosphorylated RNA present in a heterogeneous mixture of cellular RNAs. We further show that these ribozymes can capture cleaved RNAs with 3′-phosphate and 2′−3′-cyclic phosphate termini, enabling us to selectively amplify the captured RNAs. These results demonstrate their potential utility as enrichment reagents for profiling RNA cleavage products in transcriptomics studies. Our findings not only report a new catalytic reactivity in RNA but also provide insights into ribozyme evolution, primordial RNA repair, and potential applications in RNA sequencing.

## Abstract (web summary)

Catalytic RNAs, also referred to as ribozymes, likely played a crucial role in primordial RNA-based life. Here, the authors evolved ribozymes that selectively ligate RNAs bearing 3′-phosphates and show that these catalysts can be used to enrich and amplify cleaved RNAs for downstream transcriptomics.

## Introduction

Enzyme catalysis is a fundamental feature of extant life. The emergence of catalytic properties in biomolecular scaffolds is central to one of the biggest mysteries in science—the origin of life—because the appearance and evolutionary diversification of the earliest enzymes determined the fate of primordial cell lineages. Early life is thought to have used enzymes made of RNA, referred to as ribozymes, to catalyze its metabolic reactions as well as to replicate its genomes, also made of RNA^1^. Therefore, exploring the catalytic capabilities of ribozymes provides an opportunity to understand the origins of biocatalysis. The study of RNA catalysis has been greatly expanded by our ability to evolve ribozymes outside the cell^2,3^. In vitro selection from completely randomized RNA sequence libraries has yielded ribozyme chemistries that do not exist in nature, and directed evolution of existing ribozymes has resulted in the isolation of new ribozymes with different and sometimes unexpected functions^4^. Although in vitro selection protocols employ controlled selection pressures to isolate ribozymes with desired functions, it is not uncommon for ribozymes with unexpected functions to emerge from such experiments^5,6^. In those cases, ribozymes with unexpected activities use alternative (and often unforeseen) reaction pathways to get enriched through a selection protocol designed to yield a different desired activity. More than thirty years of artificial ribozyme evolution experiments have revealed that RNA can support a diversity of catalytic phenotypes^4^. However, considering the small number of ribozyme selection experiments that have been carried out to date compared to the range of interesting metabolic and replication-related reactions that could potentially be catalyzed by RNA, it is clear that we have not yet approached the full catalytic potential of RNA.

The diversity of ribozyme activities is well illustrated by RNA ligases. These ribozymes catalyze ligation reactions between the activated 5′-phosphate of an RNA substrate and either the 2′-hydroxyl, 3′-hydroxyl, or 5′-phosphate of a second RNA^7–9^. Some ligases require an external template, while others do not^7,8^. Although certain ligase ribozymes function as true enzymes by catalyzing ligation between two separate RNA oligonucleotides in trans, the majority facilitate the ligation of an oligonucleotide substrate to itself^7,8^. The many variations of a simple RNA ligase phenotype further highlight the possibility of discovering new but related ribozyme reactivities. In this work, we report the serendipitous discovery of a new ligase activity, where the ribozyme catalyzes a reaction between its 5′-triphosphate group and the 2′-hydroxyl group of an RNA oligonucleotide substrate only when the substrate possesses a 3′-phosphate group vicinal to its 2′-hydroxyl nucleophile. Ribozymes with this activity were isolated from an in vitro evolution experiment designed to evolve ribozymes that catalyze ligation with 5′-triphosphorylated RNA substrates. It is interesting that of the isolated sequences, >60% exhibited this unexpected ligase activity, while only ~30% exhibited the desired ‘triphosphate ligase′ activity via the generation of a canonical 3′−5′ phosphodiester linkage between the ribozyme and the substrate.

The absolute requirement for a 3′-phosphate group on the RNA substrate, together with ligation through its 2′-hydroxyl group, represents a surprising variation of the standard ribozyme ligase reactivity. Curiously, this reactivity is reminiscent of protein-based RNA ligase enzymes in extant biology that perform RNA repair in tRNA maturation pathways and in response to cellular stress^10^. In that context, we outline plausible roles for such ribozymes in genome repair in the extinct biology of the RNA World. The unique specificity of the isolated ribozymes toward 3′-phosphorylated RNAs opens the possibility of adapting these ribozymes as reagents for the specific enrichment of cleaved RNAs from cells. To that end, we show that RNA cleavage products containing 3′-phosphorylated ends can be selectively captured from cellular RNA using these ribozymes, enabling their amplification. In addition to reporting a novel enzyme reactivity, our work demonstrates that a single selection pressure can lead to the enrichment of multiple catalytic functions and highlights the versatility of RNA as a catalytic molecule.

## Results

### New ligase ribozymes isolated from in vitro evolution

**Fig. 1:**
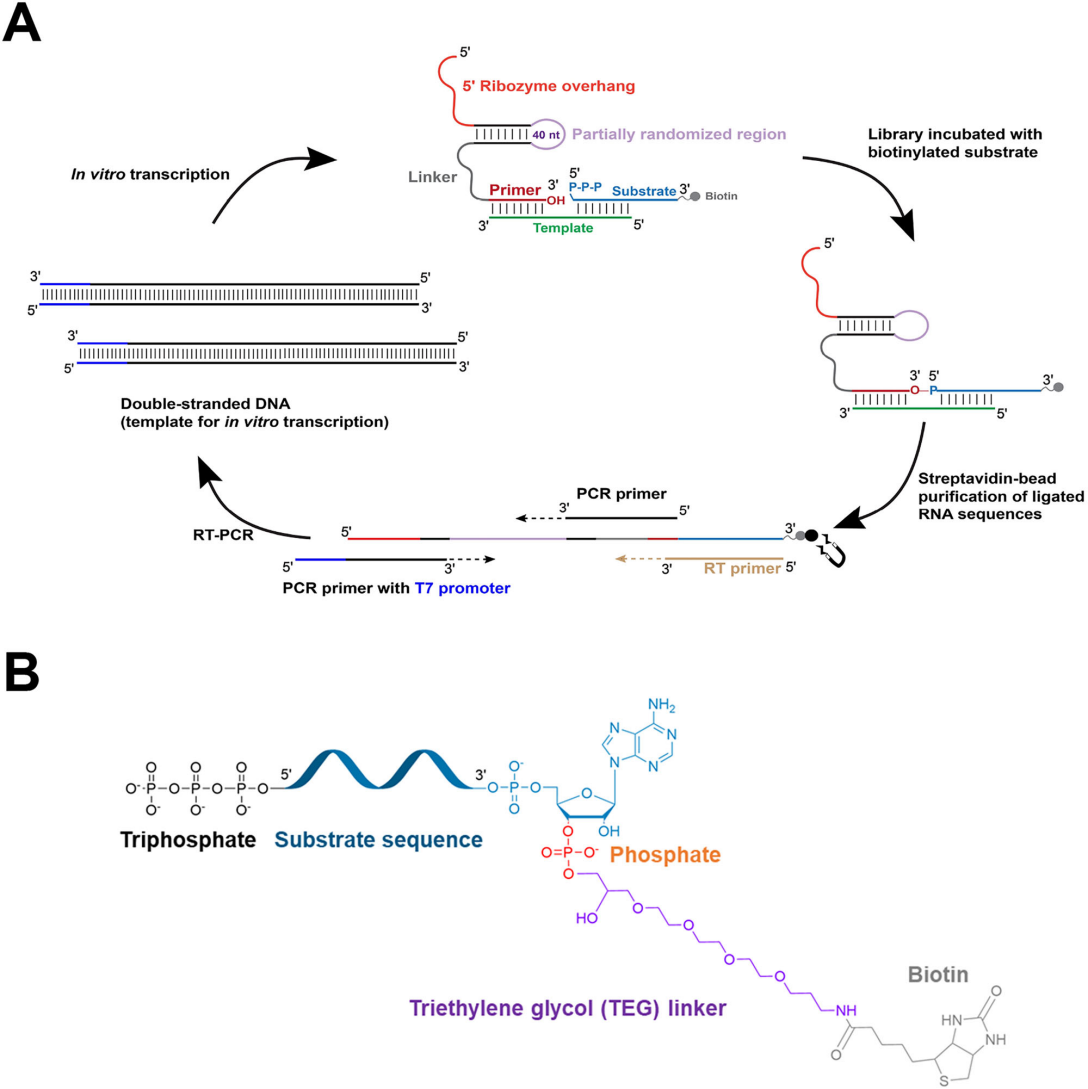
Selection protocol and substrate used to isolate RNA ligase ribozymes. **A** An RNA library containing a partially randomized sequence having an estimated complexity of ~10^14^ sequences (depicted in light purple) derived from an AIP-Ligase (RS1)^12^ was challenged with an RNA substrate (depicted in light blue), containing a 5*’*-triphosphate group and a 3*’*-TEG-biotin group (depicted in gray) in the presence of an RNA template (depicted in green). Ligated sequences were purified by binding to streptavidin-coated magnetic beads and reverse transcribed using a primer (RT primer; depicted in gold) that is complementary to the entire substrate sequence. The RT primer also has the potential to bind directly to the library sequences by forming four base-pairs with the 3*’* end of their ‘primer’ sequence (depicted in red). The cDNA was PCR-amplified, with the T7 promoter sequence (depicted in blue) added to the dsDNA sequence during PCR. This dsDNA was transcribed to generate the library for subsequent rounds of selection. All sequences are included in Supplementary Data Table 1. **B** The chemical features of the substrate used in the selection (PPP-Substrate-Biot). The substrate contains a triphosphate group (black) on its 5*’* end and is connected to a biotin moiety (gray) via a triethylene glycol (TEG) linker (purple) attached to the 3*’*-phosphate (orange) on the terminal adenine of the substrate.

Recently, we used in vitro evolution to ask whether and how ligase ribozymes might switch substrate specificity from RNA oligonucleotides activated with a 5′-phosphorimidazole group (5′-AIP) to those containing the biologically relevant 5′-triphosphate group (5′-PPP)^11^. In that work, we created a partially randomized RNA library derived from a previously characterized ‘phosphorimidazolide ligase′ (AIP-Ligase)^12^ by mutagenizing a 40-nt region of this ribozyme at 21% per nucleotide position. This variable region was flanked by constant regions, which provide binding sites for reverse transcription and PCR primers. The 3′ constant region included an 8-nt ‘primer′ sequence connected to the rest of the construct by a hexauridine linker. The terminal nucleotide of this primer sequence (i.e., the last nucleotide of the selection construct) was the intended site of ligation (Fig. 1A). Library sequences with the ability to ligate to a 16-nt RNA substrate containing a 5′-triphosphate group and 3′-biotin tag were purified away from unreactive sequences by streptavidin bead capture and subsequently amplified by RT-PCR and in vitro transcription (Fig. 1). The substrate sequence was connected to a triethylene glycol (TEG) linker by its 3′-phosphate group, and the TEG linker was in turn covalently attached to a biotin group (Fig. 1B). The target reaction for this selection involved a nucleophilic attack of the 3′-hydroxyl (3′-OH) end of the selection construct (the ‘primer′) on the α-phosphate of the 5′-PPP group of the substrate. (Fig. 1). Although we isolated five distinct classes of ligase ribozymes from that experiment, only one ribozyme class was found to catalyze the desired reaction^11^. Here, we report that the other ribozyme classes that collectively covered >60% of the isolated RNA population catalyze a new and unexpected variation on this standard mode of RNA ligation.

**Fig. 2:**
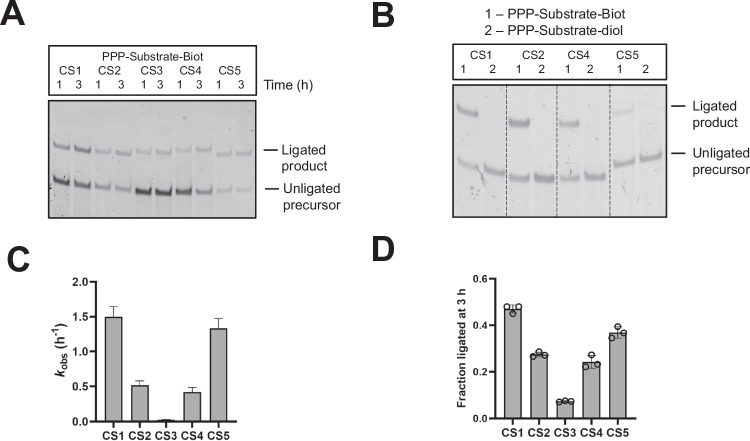
Ligase activities of the isolated sequences. **A** Peak sequences from clusters 1–5, CS1-CS5, catalyze ligation with a substrate containing a 5*’*-triphosphate group and 3*’*-TEG-biotin group (PPP-Substrate-Biot). **B** CS1, CS2, CS4, and CS5 do not ligate to a substrate oligonucleotide with a 2*’*,3*’* cis-diol (PPP-Substrate-diol). Ligation was assayed at 3 h. **C** CS1, CS2, CS4, and CS5 exhibit *k*_obs_ values of 0.4–1.5 h^-1^, but CS3-catalyzed ligation is significantly slower with PPP-Substrate-Biot. Error bars indicate standard error of the mean (S.E.M). **D** CS1, CS2, CS4, and CS5 ligated to 20–50%, while CS3 ligated to ~7% with PPP-Substrate-Biot in 3 h. Error bars indicate standard deviation. Data in (**C**) and (**D**) were obtained from triplicate measurements. Ligation reactions contained 1 µM ribozyme, 1.2 µM RNA template, and 2 µM RNA substrate, PPP-Substrate-Biot, in 100 mM Tris-HCl (pH 8.0), 300 mM NaCl, and 100 mM MgCl_2_. Experiments were performed at least in triplicate. Source data are provided as a Source Data file.

Outputs from each round were analyzed by high-throughput sequencing^13^. Closely-related sequences isolated from the last round (round 6) were binned into clusters. Collectively, these sequences represented >90% of the entire selected population (Table 1). The peak sequences of the five most abundant clusters, referred to henceforth as CS1-CS5 (Table 1, Supplementary Fig. 1) were 10–28 mutations from the parent AIP-Ligase, RS1 (Table 1). We tested CS1-CS5 for their capacity to ligate to the 5′-triphosphorylated, 3′-biotinylated substrate (henceforth, PPP-Substrate-Biot) used in each selection round (Fig. 1B). CS1, CS2, CS4, and CS5 catalyzed ligation with rates between 0.4 h^−1^ and 1.5 h^−1^, yielding between ~20% and ~50% ligated product after 3 h. In contrast, CS3 showed reduced activity with a ~ 75-fold lower ligation rate than CS1 (Fig. 2A, C, D). Interestingly, only CS3 catalyzed the desired ligation reaction with a PPP-Substrate, which we previously reported as a bona fide triphosphate ligase ribozyme^11^. CS1, CS2, CS4, and CS5, on the other hand, appeared to exhibit an unexpected dependence on the 3′ biotinylation state of the substrate (Fig. 2B). This indicated that CS1, CS2, CS4, and CS5 do not catalyze the desired reaction. However, the appearance of a ligated product when incubated with PPP-Substrate-Biot suggests that these sequences are ligases that use an unforeseen reaction pathway. In the following sections, we investigate the unexpected features of these ligases and uncover a novel enzyme reactivity.

**Table 1:** Sequence alignment of the isolated ligase ribozymes

The 40-nt variable region from the dominant sequences of the five most abundant sequence families. RS1 is the parent AIP-Ligase that CS1, CS2, CS4 and CS5 were evolved from. Sequence divergence from RS1 is highlighted in gray.

### Unexpected reaction between the substrate 2′-hydroxyl and the ribozyme 5′-triphosphate groups

**Fig. 3:**
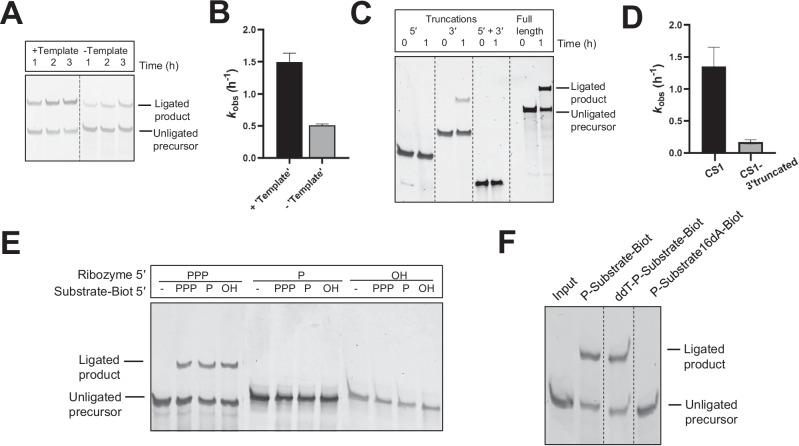
Untemplated ligation between the 5*’*-triphosphate group of CS1 and the 2*’*-hydroxyl group of the substrate. **A**, **B** CS1-catalyzed ligation is preserved in the absence of an external template with only a 3-fold decrease in activity. **C** Truncating CS1 by deleting its first 25 nucleotides abrogates ligation; however, deleting its last 14 nucleotides preserves activity. **D** Deleting 14 nucleotides from the 3*’* end of CS1 results in 8-fold slower ligation than ligation catalyzed by a full-length CS1 ribozyme. Data in (**B**) and (**D**) were obtained from triplicate measurements, where error bars indicate standard error of the mean (S.E.M). **E** Ligation requires a triphosphate group at the ribozyme 5*’* end but is agnostic to the chemistry at the substrate 5*’* end. **F** Blocking the 5*’* end of the substrate with an inverted dideoxythymidine group (ddT-P-Substrate-Biot) preserves ligation; however, a substrate with a 2*’* terminal deoxyribonucleotide (P-Substrate16dA-Biot) fails to ligate to CS1. Ligation reactions contained 1 µM ribozyme and 2 µM RNA substrate (PPP-Substrate-Biot unless otherwise specified) in 100 mM Tris-HCl (pH 8.0), 300 mM NaCl, and 100 mM MgCl_2_. Reactions do not contain an external template unless indicated (**A**) at 1.2 µM concentration. Ligation reactions in (**E**) and (**F**) were assayed at 3 h. Experiments were performed at least in triplicate. Source data are provided as a Source Data file.

Given that CS1, CS2, CS4, and CS5 all exhibited an identical apparent dependence on the 3′ biotinylation state of the substrate, we selected the most abundant and active sequence, CS1, for detailed biochemical characterization. The desired reaction was a templated ligation between the 3′ and 5′ termini of the ribozyme and substrate, respectively, where the 16-nt template oligonucleotide was expected to bring the two RNA termini together by forming 8 base-pairs with sequences at the 3′ and 5′ end of each RNA (Fig. 1A). However, CS1-catalyzed ligation was only 3-fold slower in the absence of the external template (Fig. 3A, B). This absence of template requirement was reminiscent of a previous in vitro selection experiment that unintentionally isolated ribozymes that catalyze a ligation reaction between the ribozyme 5′-PPP and the 5′-phosphorimidazole moiety of an RNA substrate, generating a 5′-5′ linkage^9^. To test the possibility that we had accidentally selected similar 5′-5′ ligases, we assayed the ligase activities of truncated versions of CS1, created by deleting either its first 25 nucleotides (5′ truncation or 5′t) or its last 14 nucleotides (3′ truncation or 3′t). While the 3′ truncation preserved ligation, albeit 8-fold slower, the 5′ truncated ribozyme was inactive (Fig. 3C, D). This suggested that the deleted 25-nt sequence at the ribozyme 5′ end is important for its activity, either by being part of its active fold or by directly participating in ligation. To decouple the potential structural vs. functional roles of the 5′ sequence of CS1, we tested CS1 variants containing either a triphosphate (PPP), monophosphate (P), or hydroxyl (OH) group at their 5′ ends against substrate variants with either a 5′-PPP, 5′-P, or 5′-OH. While all substrate variants ligated to CS1, only a 5′-triphosphorylated CS1 retained activity (Fig. 3E). Efficient ligation with 5′-P or 5′-OH substrates discounted the possibility of a nucleophilic attack by the ribozyme 5′-PPP on the substrate 5′ end as observed in the case of the 5′-5′ ligase^9^. The inactivity of CS1 without a 5′-PPP, on the other hand, indicated that the ribozyme 5′ end was the likely site of nucleophilic attack by the substrate. In principle, a promiscuous ribozyme could catalyze the nucleophilic attack of substrate 5′-PPP, 5′-P, or 5′-OH groups on its own 5′-α-phosphate, with the release of a pyrophosphate group^6,14,15^. However, similar reactivities of all three substrates, under a range of different conditions, suggested that the 5′ end of the substrate did not participate in ligation. CS1 exhibited similar reaction rates (*k*_obs_ = ~1.5 h^−1^) with all three 5′-modified substrates (Supplementary Fig. 2) and exhibited comparable Mg^2+^ requirements with substrates possessing 5′-PPP or 5′-OH groups: [Mg^2+^]_1/2_ (OH-Substrate-Biot) = ~15 mM; [Mg^2+^]_1/2_ (PPP-Substrate-Biot) = ~20 mM (Supplementary Fig. 3). Additionally, CS1 showed similar pH-rate profiles (log *k*_obs_ vs pH) when ligating 5′-P or 5′-OH substrates, exhibiting linearity with a slope of ~1 between pH 6.5 and 8 for both reactions (Supplementary Fig. 4). This usually indicates a single H^+^ transfer in the rate-determining step in this pH regime involving the nucleophilic hydroxyl group on the substrate^16^. The irrelevance of the substrate 5′ chemistry was conclusively shown by the ligation of CS1 to a substrate variant containing an inverted dideoxythymidine(ddT) group blocking its 5′ end (Fig. 3F).

With the 5′ end of the substrate discounted as a reactive center and the 3′ end of the substrate being blocked by TEG-biotin, we turned to the 2′-OH of the terminal adenine residue of the substrate as the most likely candidate for the nucleophile. A substrate where the terminal adenine ribonucleotide (rA16) was replaced by a deoxyribonucleotide (dA16) was inactive for ligation, confirming the substrate terminal 2′-OH as the nucleophile (Fig. 3F and Supplementary Fig. 5A). Identical results with CS2, CS4, and CS5 established a common reactivity for all four ligases (Supplementary Fig. 5B). Nucleophilic attack of the substrate 2′-OH on the ribozyme 5′-PPP would generate a noncanonical 2′-5′ linkage between the ribozyme and substrate. We performed a dual exonuclease digestion assay to test for the presence of this noncanonical 2′-5′ linkage. We purified the products of ligation between CS1 and substrates with either 5′-PPP or 5′-P groups and subjected them to 5′-P-dependent Terminator 5′→3′ exonuclease digestion. As expected, the 5′-PPP ligated product was resistant to degradation (negative control), but the digestion of the 5′-P ligated product yielded RNA that was comparable in size to the ribozyme (Supplementary Fig. 6A). On the other hand, 3′→5′ RNase R digestion of the ligated product generated RNA that was one nucleotide longer than the substrate (Supplementary Fig. 6B). These results suggest the presence of a 2′-5′ phosphodiester bond between the ribozyme and substrate and are consistent with a reaction between substrate terminal 2′-OH and ribozyme 5′-PPP groups.

The reactivity described above also explains the higher-order ligation products generated during CS1-catalyzed ligation with a 5′ 2AI-activated substrate (AIP-Substrate-Biot) in the presence of the external template and 100 mM Mg^2+^ (Supplementary Fig. 7). These higher-order products are concatemeric RNA sequences with a heterogenous backbone composed of both 2′-5′ and 3′-5′ phosphodiester linkages formed as a result of a combination of ribozyme-catalyzed 2′-5′ ligation and template-directed nonenzymatic 3′-5′ ligation at a high Mg^2+^ concentration. The production of concatemeric RNA demonstrates the ribozyme′s ability to function in the context of longer transcripts.

### Ligation requires a 3′-phosphate group on the substrate

**Fig. 4:**
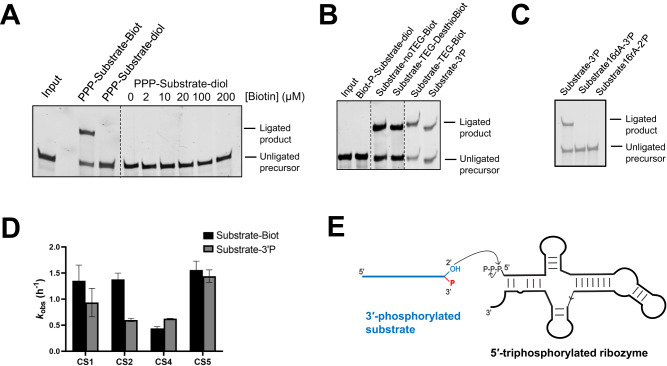
Ligation requires a 3*’*-phosphate group on the substrate. **A** Ligation with an unbiotinylated substrate containing a terminal cis-diol is not rescued upon the addition of free biotin. **B** Ligation is abolished when the TEG-biotin moiety is moved to 5*’* end of the substrate, but a substrate with the biotin group replaced by a desthiobiotin group retains the ability to be ligated. A substrate with a 3*’*-phosphate group is active for ligation. **C** A substrate with 2*’* phosphate and 3*’* hydroxyl groups (Substrate16rA-2*’*P) is not ligated, indicating that only the 2*’*-OH is catalytically-activated for nucleophilic attack in the presence of a vicinal 3*’*-P, but not a 3*’*-OH in the presence of a vicinal 2*’*-P. **D** Ligation rates for CS1, CS2, CS4, and CS5 with substrates containing terminal TEG-biotin or 3*’*-phosphate groups are comparable. Data were obtained from triplicate measurements, where error bars indicate standard error of the mean (S.E.M). **E** The isolated ligase activity involves the nucleophilic attack of the 2*’*-OH group of a 3*’*-phosphorylated substrate on the 5*’*-triphosphate group of the ribozyme. The secondary structure of the ribozyme depicted here is the SHAPE-derived structure of CS1 (see Supplementary Fig. 13B). Ligation reactions contained 1 µM ribozyme, 1.2 µM template and 2 µM RNA substrate in 100 mM Tris-HCl (pH 8.0), 300 mM NaCl, and 100 mM MgCl_2_. Biotin was added to the reaction as indicated in (A). Ligation reactions in (**A**–**C**) were assayed at 3 h. Experiments were performed at least in triplicate. Source data are provided as a Source Data file.

The unexpected observation that substrates without the 3′-‘TEG-biotin′ moiety (i.e., with a 2′,3′ diol) are not substrates for ligation catalyzed by CS1, CS2, CS4, and CS5 (Fig. 2B) pointed to a potential role for the 3′-biotin tag in this reaction. As ribozymes that utilize thiamin (Vitamin B1) as cofactor have been reported^17^, we wondered if CS1, CS2, CS4, and CS5 might utilize biotin (Vitamin B7) for catalysis. We found that supplementing a reaction between CS1 and an unbiotinylated substrate with free biotin did not rescue ligation (Fig. 4A), moving the biotin modification from the 3′ to the 5′ end of the substrate eliminated ligation (Fig. 4A), and replacing the biotin moiety with desthiobiotin (biotin without a S atom) preserved ligation (Figs. 1B, 4B). These results collectively cast doubt on the direct involvement of biotin. Deleting the TEG spacer between the substrate 3′-phosphate and the biotin (so that the 3′-phosphate was directly connected to the biotin) also preserved ligation (Fig. 1B, Fig. 4B). Importantly, CS1 ligated a substrate that lacked TEG-biotin but possessed a 3′-P group (Substrate-3′P) with rates comparable to that of a biotinylated substrate (Fig. 4B, D). Therefore, the apparent dependence of CS1 on the substrate 3′-‘TEG-biotin′ was, in fact, a requirement for a 3′-phosphate group on the substrate. CS2, CS4, and CS5, like CS1, ligated to Substrate-3′P revealing that the isolated ribozymes represented a new class of ligases that catalyze a reaction between its 5′-triphosphorylated end and the 2′-OH group of a 3′-phosphorylated oligoribonucleotide substrate (Fig. 4D, E).

The essential role for the substrate 3′-phosphate is underscored by the rate acceleration of over 5 orders of magnitude relative to the background ligation rate (background ligation between a 5′-PPP-RNA oligonucleotide corresponding to the first 25 nt of the ribozymes and a 5′-FAM labeled 3′-phosphorylated substrate was measured as 2.4 × 10^−6 ^h^−1^ at pH 8 and 100 mM Mg^2+^). Although a 3′-P can, in principle, accelerate substrate ligation by activating the vicinal 2′-OH nucleophile by acting as a general base catalyst, similar ligation rates with Substrate-3′P, which contains a phosphomonoester with a pKa ∼7 and Substrate-TEG-Biot (Fig. 4D), which contains a phosphodiester with a pKa ∼2, discount this potential catalytic role. In fact, the presence of 3′-phosphate or 3′-phosphodiester groups lowers the nucleophilicity of the vicinal 2′-hydroxyl group^18^, which is supported by a slight increase in the 2′-OH pKa in the presence of a 3′-phosphate relative to a 3′-hydroxyl (~13.4 vs. ~12.4)^19^. A more likely role of the 3′-P is in localizing catalytic divalent cations to the active site. Ribozymes use active site-bound divalent metal ions in diverse catalytic mechanisms, including RNA cleavage and RNA ligation reactions^20–22^. Crystal structures of the L1 ligase and the class I ligase ribozymes, both of which catalyze ligation between the substrate 3′-OH and a ribozyme 5′-PPP, reveal that an active site Mg^2+^ interacts with a non-bridging oxygen on the α-phosphate of the ribozyme 5′-PPP and also with the ribozyme phosphodiester backbone^23,24^. In addition, the structure of the class I ligase suggests a possible interaction between Mg^2+^ and the 3′-O nucleophile^24^. Similar interactions in these ligases between active site Mg^2+^ ions and the 3′-P group of the substrate and pyrophosphate leaving group of the ribozyme 5′-PPP could be important in catalysis. To examine the nature of the interaction between the terminal phosphate group and Mg^2+^, we measured reaction kinetics with substrates containing either a terminal phosphate or a thiophosphate in the presence or absence of the thiophilic cation Cd^2+^ in the background of Mg^2+^. We observed a 2-fold reduction in ligation rate with a terminal thiophosphate-containing substrate (Substrate-3′sP). When the reaction was supplemented with Cd^2+^ (25 µM or 1 mM), ligation was restored with a metal rescue value of ~2.5 (Supplementary Fig. 8A, B). More pronounced thio effects and metal rescue values have been reported in ribozymes where Mg^2+^ directly interacts with either of the non-bridging oxygens of the phosphate group^22^. A diminished effect is expected as our experiment does not involve a stereospecific O to S substitution in the phosphate. The modest effect we observed might also reflect a weak inner-sphere Mg^2+^ interaction. Substitution of Mg^2+^ with the divalent metal ions Ba^2+^, Ca^2+^, Co^2+^, Cu^2+^, Mn^2+^, Ni^2+^ abolished ligation, consistent with a possible catalytic role for Mg^2+^ (Supplementary Fig. 8C). The specific requirement for the substrate terminal configuration, i.e., 2′-OH, 3′-P was further highlighted by the lack of reactivity of a substrate with a 3′-OH, 2′-P terminus (Substrate16rA-2′P) (Fig. 4C).

As the isolated ribozymes catalyze substrate ligation to their 5′ ends as opposed to their 3′ ends, we were surprised that these sequences could be reverse transcribed at 42 °C using a primer (RT primer) that binds to its 3′ end by forming just 4 base-pairs. This indicates that the isolated sequences survived this step in the selection cycle by exploiting weak interactions between the RT primer and their 3′ ends (Supplementary Fig. 9). Because this ligation pathway joins the 2′ end of the substrate to the 5′ end of the ribozyme, the external template cannot be responsible for bringing these two ends into close proximity, as it does for the expected ligation junction between the ribozyme 3′ end and the substrate 5′ end (Supplementary Fig. 9). Furthermore, since the CS1 ribozyme does not require the external template, we were surprised to find that CS2, CS4, and CS5 did require an external template for activity (Supplementary Fig. 10A). We hypothesized that this template dependence was due to functional base-pairing interactions between the ribozyme 3′ sequence (‘primer′) and the template (Supplementary Fig. 10B). Disrupting base pairs between the primer and the template through template mutations eliminated ligation, which was rescued by compensatory mutations in the ribozyme 3′ primer, supporting this hypothesis (Supplementary Fig. 10C). We found similar interaction between the 3′ end of CS1 and the template (Supplementary Fig. 10D), which explains the decrease in ligation observed in the absence of the template (Fig. 3A, B) and the 3′-truncated version of this ribozyme (Fig. 3C, D). Base-pairing interactions between the template and both the 3′ end of the ribozyme and the 5′ end of the substrate may make this three-component reaction pseudo-intramolecular, thereby stimulating ligation.

### Comparative structural analysis of the isolated ligase ribozymes

Although at first glance, CS1, CS2, CS4, and CS5 diverge from each other by 11–22 mutations (Supplementary Fig. 11), which could indicate distinct structural folds, upon more careful inspection, we found that the sequences, especially CS1, CS2, and CS4, could be aligned to highlight structural similarities (Supplementary Fig. 12). To get a better understanding of their secondary structures, we performed SHAPE-probing on CS1, CS2, CS4, and CS5. Constraining their computationally predicted secondary structure with SHAPE reactivities yielded structures that differed significantly from each other and from their parent ligase, RS1 (Supplementary Fig. 13). Despite this apparent structural divergence, we noticed similarities in SHAPE reactivity patterns across all four sequences (Supplementary Fig. 12). The combination of common SHAPE reactivity patterns and comparative sequence analysis revealed the possibility that these ligases, at least CS1, CS2, and CS4, share a common fold.

We identified regions that may form conserved base-paired stems in all four RNAs (Supplementary Fig. 12). In CS1, CS2, and CS4, one of the putative stems consists of 5′-CCACUCA-3′ and 3′-GGUGAGU-5′ regions, while in CS5, the putative stem is shorter, composed of 5′-CUCA-3′ and 3′-GAGU-5′ (shown in green in Supplementary Fig. 12). The presence of this stem in all four RNAs is supported by their overall insensitivity to SHAPE modification. The SHAPE-derived structures of CS1, CS2, and CS4 feature this stem, whereas in CS5, the relevant residues (5′-CUCA-3′ and 3′-GAGU-5′) appear unpaired despite showing low reactivities (Supplementary Fig. 13). The fact that this stem is composed of nucleotides in the constant region and those that emerged as a result of selection increases the likelihood of its presence in these ribozymes. The presence of a second putative stem composed of 5′-GGACAGCG-3′ and 3′-CCUGUCGC-5′ regions (shown in blue in Supplementary Fig. 12) is less certain as residues in the 3′-strand sequence were reactive in SHAPE experiments in CS1 and CS4, indicating a lack of base-pairing. Further prediction is complicated by the lack of reactivity data for a significant portion of this region (Supplementary Figs. 12, 13). Even without SHAPE data for the 3′ end of the ribozymes, the fact that the last eight nucleotides of CS1, CS2, CS4, and CS5 base-pair with the template during ligation (Supplementary Fig. 10) indicates that they are likely unpaired in the ribozyme′s secondary structure. We also noticed high SHAPE reactivities in the 5′-AAUGA-3′ region (residues 66-70) in all four ribozymes, indicating that this region is unpaired, further pointing to the possibility of a shared structure.

In light of these observations, we queried for a common secondary structure for CS1, CS2, CS4, and CS5 using TurboFold, an iterative probabilistic RNA secondary structure prediction algorithm for estimating common secondary structures for multiple sequences, even when they exhibit substantial divergence^25^. TurboFold outputs converged on a common secondary structure for CS1, CS2, and CS4, but predicted a different structure for CS5 (Supplementary Fig. 14). Structures of CS1, CS2, and CS4, as predicted by TurboFold, featured a base-paired stem composed of 5′-CCACUCA-3′ and 3′-GGUGAGU-5′ regions similar to their SHAPE-derived structures; however, unlike its SHAPE-derived structure, the structure of CS5 obtained from TurboFold featured the shorter stem composed of 5′-CUCA-3′ and 3′-GAGU-5′, we identified from sequence analysis (highlighted by a green a box in Supplementary Fig. 14). TurboFold did not predict the existence of the second putative stem composed of 5′-GGACAGCG-3′ and 3′-CCUGUCGC-5′, which is consistent with the SHAPE reactivity of certain residues in this region. Some of the inconsistencies between the secondary structures derived from SHAPE probing and those predicted by sequence inspection or TurboFold may arise due to misfolding of the ribozyme in the absence of the template and substrate RNAs in SHAPE experiments. Regardless, in the absence of high-resolution structures, it is difficult to claim structural relationships between these ligases, although there are likely significant similarities due to the observations outlined above.

### Specific capture and amplification of 3′-phosphorylated RNA

This reactivity of ligating specifically to 3′-phosphorylated RNAs exhibited by the isolated ribozymes is also interesting in the context of extant biology. RNAs with terminal phosphates in the form of 2′, 3′-cyclic phosphates (cP) and 3′-phosphates (3′-P) are generated as products of enzymatic cleavage pathways in RNA processing and maturation and have been implicated in diseases including cancers, amyotrophic lateral sclerosis, tuberculosis, and Parkinson′s disease^26–31^. However, cleaved RNAs constitute a poorly characterized portion of the transcriptome, primarily because they remain invisible to standard library preparation protocols used in high-throughput sequencing. This is due to the inability of 3′-phosphorylated cleaved RNAs to ligate to the 3′ sequencing adapter because they lack free 3′-hydroxyl groups. The handful of recently reported methods for sequencing terminal phosphate-containing RNAs cannot distinguish between cP and 3′-P-containing RNAs due to the promiscuity of the RNA ligases used (*Arabidopsis thaliana* tRNA ligase or RtcB ligase) or rely on indirect enrichment of cP-RNAs via periodate cleavage of RNA terminal diols^32–35^. As the ribozymes identified in this work show absolute discrimination between 3′-OH and 3′-P RNA termini we explored their potential application as reagents for the specific enrichment of 3′-phosphorylated RNA from total cellular RNA.

**Fig. 5:**
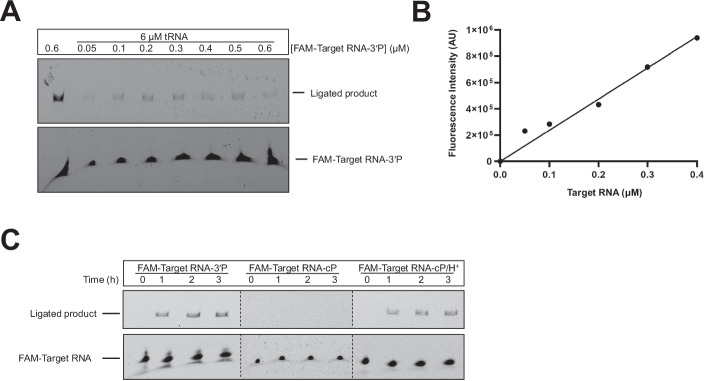
Ribozyme-assisted capture of 3*’*-phosphorylated RNA. **A** CS1 captures a FAM-labeled 3*’*-phosphorylated substrate, FAM-Target RNA-3*’*P, from a heterogeneous mixture of cellular tRNAs. **B** Capture, as measured by the fluorescence intensity of the ligated product, is linear in response to substrate concentration between 0.05 µM and 0.4 µM. **C** CS1 shows specificity toward substrate 3*’* termini. It specifically ligates to substrates with 3*’*-phosphate groups, while being inert to those terminating in 2*’*, 3*’*-cyclic phosphate (cP) groups. Acid hydrolysis of the terminal cP group makes these RNAs substrates for ribozyme-assisted capture. Ligation reactions contained 1 µM ribozyme, CS1, and the indicated amounts of FAM-Target RNA-3*’*P (**A**, **B**) or 2 µM RNA targets (FAM-Target RNA-3*’*P or FAM-Target RNA-cP) in 100 mM Tris-HCl (pH 8.0), 300 mM NaCl, and 100 mM MgCl_2_. Capture reactions in (**A**) were assayed at 3 h. Experiments were performed in triplicate. Source data are provided as a Source Data file.

We found the CS1 has a low *K*_M_ value of 0.1649 ± 0.042 µM and a high catalytic efficiency (*k*_cat_/ *K*_M_ value) of 27.65 µM^−1^ h^−1^ (7670 M^−1^ s^−1^) indicating that CS1 could function as a potential reagent for enriching 3′-P-RNAs (Supplementary Fig. 15). We spiked in different concentrations a 5′ FAM-labeled 3′-phosphorylated substrate (FAM-Target RNA-3′P) into an *E. coli*-derived tRNA mix and incubated this mixture with CS1. The appearance of a band corresponding to the ligated product even when tRNAs were in 60-fold excess supports the ribozyme′s ability to enrich 3′-phosphorylated RNAs from a heterogeneous mixture of cellular RNA (Fig. 5A). The fluorescence signal from the ligated product increased linearly with substrate concentration between 0.05 µM to 0.4 µM, suggesting a potential for quantitative detection of 3′-P-RNAs (Fig. 5B). The captured 3′-P-RNAs must be first reverse transcribed and then PCR-amplified to generate material that can be sequenced (Supplementary Fig. 16A). To see whether the 3′-P next to a 2′-5′ phosphodiester linkage would prevent reverse transcription, we compared RT-PCR before and after removing the phosphate group by shrimp alkaline phosphatase (SAP). Interestingly, the target RNA was amplified even without SAP treatment, showing that reverse transcriptase can copy across the unusual 2′-5′ phosphodiester linkage harboring an adjacent 3′-phosphate (Supplementary Fig. 16B).

These ribozymes may also be used to indirectly capture cP-RNAs by converting their cP ends to 3′-P (and 2′-P) by mild acidification. Upon incubating a cP-containing substrate (FAM-Target RNA-cP) pre-treated with 10 mM HCl (0 °C/3 h)^35^ with CS1, we observed a steady increase in ligation with increasing incubation time, indicating cP-RNA capture. FAM-Target RNA-cP, not subjected to the acidification step, was not captured by the ribozyme (Fig. 5C). As self-cleaving ribozymes generate cleavage products with cP ends, this ribozyme-assisted enrichment method, in conjunction with standard RNA-seq, may be used in high-throughput screens to discover new self-cleaving ribozymes^36,37^. For maximal utility as RNA sequencing reagents, ribozymes must enrich RNAs in a sequence-general manner. Therefore, we tested the substrate scope of ribozyme-assisted target capture. We tested CS1 against truncated versions of the Substrate-3′P, which has the sequence: 5′-ACCACCGCAUUCCGC**A**p-3′, where **A** contains the 2′-OH nucleophile. CS1 captured a target representing the last 8 residues of Substrate-3′P (5′-AUUCCGC**A**p-3′) but was unable to ligate an oligoribonucleotide representing its first 8 residues (5′-ACCACCGCp-3′) (Supplementary Fig. 17A). Target 5′-AUUCCGC**A**p-3′ was further truncated to two 5 nt pieces and tested for ligation. Once again, the piece containing the nucleophilic adenine (5′-CCGC**A**p-3′) was captured but 5′-AUUCCp-3′ was not (Supplementary Fig. 17A). Shorter oligomers were not tested due to the difficulty in resolving captured products from the ribozyme by denaturing gel electrophoresis. Substrates, where their terminal A was replaced by C or U, could not be captured by CS1, and a substrate with a 3′ terminal G showed ~500-fold slower ligation with CS1 (Supplementary Fig. 17B, C). CS2, CS4, and CS5 showed similar dependence on the identity of the 3′ terminal residue of the substrate (Supplementary Fig. 18). These results indicate the importance of the adenine nucleotide at the 3′ end of the substrate. Similarly, the ribozyme 5′ end was constrained to a guanine; a G to A mutation in all four ribozymes abolished ligation with all four substrate variants (Supplementary Figs. 17D, 18). These constraints on both ligation junction nucleotides suggest that there is an internal template that brings them together to allow ligation; however, the identity of this internal template, if it exists, remains to be defined.

## Discussion

Directed evolution of RNA has made it possible to experimentally access RNA sequences with new functions from existing ones. Although in most cases, RNAs with a desired target function have been identified, there are several examples where unexpected functions have emerged^5,6,9^. Thus, a single selection condition may give rise to multiple RNA activities as long as the activity allows the sequences to survive the selection pressure, i.e., exhibit the required fitness. In this work, we isolated ligase ribozymes that catalyze a reaction between the 2′-OH group of an RNA substrate and the 5′-PPP group of the ribozyme. The isolated activity is the opposite of the targeted activity—the nucleophilic attack of the ribozyme 3′-OH on the substrate 5′-PPP group. While ribozymes with 2′-5′ ligase activity have been identified before^7,38^, the ligases identified in this work present a new variation with a strict requirement for a 3′-phosphate, vicinal to the nucleophilic 2′-OH group on the substrate. The similarities of this ribozyme ligase reactivity to protein-based RNA repair ligases such as tRNA ligase and RtcB ligase^10,26^ are worth noting (Supplementary Fig. 19). tRNA ligase catalyzes a reaction between the 5′-adenyl-pyrophosphate (App) moiety of one RNA substrate and a 3′-OH group of the other, where the second RNA substrate contains a 2′-phosphate vicinal to the nucleophilic 3′-OH. This pathway resembles the ligase ribozymes reported here, wherein ligation involves a 5′-triphosphate of one RNA and the terminal 2′-OH of the other RNA, in the presence of a vicinal 3′-phosphate. RtcB ligase uses 3′-P-RNAs as substrates; however, there, the 3′-phosphate participates directly in reaction, unlike the ligase ribozymes we isolated.

It is important to recognize that designing a selection strategy to intentionally isolate 3′-P-specific ligases where the 3′-phosphate group does not directly participate in the ligation reaction would be challenging. However, we unintentionally isolated four prominent classes of ribozyme ligases with this activity. These ligases diverge considerably from the parental AIP-Ligase (by 10–16 mutations) and from each other (by 11–22 mutations). The predominance of 3′-P-specific ligases isolated from our selection suggests that our selection scheme, with minor modification, may be used to isolate more efficient catalysts, including those with expanded substrate scopes. Optimized ribozymes that ligate to RNA substrates regardless of the identity of their terminal residue, unlike those reported here, have potential to act as reagents for the transcriptome-wide probing of terminal phosphate-containing RNA cleavage products^26^. The ability to generate a complete catalog of cellular cP- and 3′-P-RNAs under various cellular conditions and in health and disease will expand our understanding of RNA cleavage and repair pathways and potentially identify disease biomarkers.

Going back in time, RNAs containing terminal phosphates would have been generated on the early Earth by non-specific phosphorylation at their 5′ and 3′ termini, and due to cleavage of the RNA backbone followed by hydrolysis of the 2′-3′-cyclic phosphate^39–41^. Excessive RNA cleavage would prevent efficient genome replication in early RNA-based lifeforms. Ribozymes capable of ligating cleaved RNAs to regenerate a continuous RNA backbone provide a salvage pathway by which the information contained within the cleaved RNA may be preserved (Supplementary Fig. 20). 5′ RNA cleavage products containing terminal 2′,3′-cyclic phosphates are easily acid-hydrolyzed to 3′-phosphates (and 2′-phosphates) generating substrates for the ribozymes reported in this work^35^. 3′ cleavage products containing 5′-hydroxyl groups may be triphosphorylated by ribozymes reported by the Muller lab in the presence of cyclic-trimetaphosphate, an abundant prebiotic reagent^42–44^. Ligation between 5′-triphosphorylated RNAs and 3′-phosphorylated RNAs catalyzed by trans versions of these ribozymes would restore the original RNA sequence before cleavage, with the 2′-5′ phosphodiester linkage being the only point of distinction. The presence of a 2′-5′ phosphodiester linkage in the ligated product does not prevent its replication^45^ and RNAs containing interspersed 2′-5′ linkages have been shown to retain their functions^46^. The viability of prebiotically plausible backbone repair pathways that convert internal 2′-5′ linkages to 3′-5′ linkages^47^ support this proposed ribozyme-assisted salvage pathway. This model complements a recently proposed nonenzymatic pathway for ‘patching′ cleaved RNAs containing 3′-phosphates via the formation of 3′-5′ pyrophosphate linkages^48^. In the wider context of the origin and evolution of early life, our results demonstrate the ease with which diverse RNA catalysts can emerge under the same selection pressure, further bolstering the case for the existence of a primordial RNA-based biology on the early Earth.

## Methods

### Materials

All chemicals were purchased from Sigma, unless otherwise specified. The hydrochloride salts of 1-ethyl-3-(3-dimethylaminopropyl) carbodiimide and 2-aminoimidazole were purchased from Alfa Aesar and Combi-Blocks, Inc, respectively. Enzymes were purchased from New England Biolabs unless mentioned. SYBR Gold Nucleic Acid Gel Stain was purchased from ThermoFisher Scientific. 100% ethanol was purchased from Decon Laboratories, Inc. QIAquick PCR purification kits were purchased from Qiagen. The Sequagel-UreaGel concentrate and diluent system for denaturing polyacrylamide gels was purchased from National Diagnostics. All DNA oligonucleotides were purchased from Integrated DNA Technologies (IDT). RNA oligonucleotides were either purchased from Integrated DNA Technologies (IDT), except for PPP-Substrate, PPP-Substrate-Biot, and Substrate16rA-2′P, which were purchased from Chemgenes, or generated by in vitro transcription (IVT) of DNA templates purchased from IDT (Supplementary Data Table 1).

### RNA preparation and substrate activation

Ribozyme constructs were prepared by in vitro transcription of dsDNA templates generated by PCR of single-stranded DNA (Supplementary Data Table 1). These dsDNA templates contained 2′-*O*-methyl modifications on the last two nucleotides of the template strand to reduce transcriptional heterogeneity at the 3′ end of the RNA^49^. Each transcription reaction containing 40 mM Tris-HCl (pH 8), 2 mM spermidine, 10 mM NaCl, 25 mM MgCl_2_, 10 mM dithiothreitol (DTT), 30 U/mL RNase inhibitor murine, 2.5 U/mL thermostable inorganic pyrophosphatase (TIPPase), 4 mM of each NTP, 30 pmol/mL DNA template, and 1U/µL T7 RNA Polymerase were incubated at 37 °C for 2–3 h. The dsDNA template was removed by DNase I-digestion (5 U/mL at 37 °C for 30 min) and the RNA was extracted with phenol-chloroform-isoamyl alcohol (Invitrogen), ethanol precipitated, and purified by 10% denaturing PAGE.

CS1 variants with 5′-phosphate (5′-P) and 5′-hydroxyl (5′-OH) groups were generated by the splinted ligation of three RNA oligonucleotide pieces (Supplementary Data Table 1). The first piece contained either a 5′-P or a 5′-OH modification. The second and third pieces were 5′-monophosphorylated to enable ligation by T4 RNA ligase 2. 60 pmol of each piece were incubated with 40 pmol of two DNA splints each at 90 °C for 3 min, followed by a 10 min incubation at 30 °C, which was followed by the addition of 1U/ µL RNA ligase 2 and 1X T4 RNA ligase buffer. The 20 µL reaction was incubated at 30 °C for 2 h, the RNA was extracted with phenol-chloroform-isoamyl alcohol, and purified by 10% denaturing PAGE.

The 5′-monophosphorylated oligonucleotide corresponding to the AIP-Substrate (Supplementary Data Table 1) was activated by reacting with 0.2 M 1-ethyl-3-(3 dimethylaminopropyl) carbodiimide (HCl salt) and 0.6 M 2-aminoimidazole (HCl salt, pH adjusted to 6) in aqueous solution for 2 h at room temperature^12^. Salts were removed by four or five washes (200 µL water per wash) in 3 kDa cutoff Amicon Ultra spin columns. This was followed by reverse phase analytical HPLC purification using a gradient of 98% to 75% 20 mM TEAB (triethylamine bicarbonate, pH 8) versus acetonitrile over 40 min.

### Ribozyme-catalyzed RNA ligation assays

Ligation reactions contained 1 µM ribozyme, 1.2 µM RNA template, and 2 µM RNA substrate in 100 mM Tris-HCl pH 8.0, 300 mM NaCl, and 100 mM MgCl_2_, unless specified. Aliquots were quenched with 5 volumes of quench buffer (8 M urea, 100 mM Tris-Cl, 100 mM boric acid, 100 mM EDTA) and analyzed on a 10% denaturing PAGE. Gels were stained using SYBR Gold and imaged on an Amersham Typhoon RGB instrument (Cytiva), Scans were analyzed in ImageQuant IQTL 8.1. Kinetic data were nonlinearly fitted to the modified first order rate equation, y = A (1−e^−^^*k*x^), where A represents the fraction of active complex, *k* is the first order rate constant, x is time, and y is the fraction of ligated product in GraphPad Prism 9. Reported rate constants and errors were obtained directly from curve-fitting of triplicate time-course datasets on GraphPad Prism.

### Confirmation of a 2′-5′ phosphodiester bond in the ligated product

*Terminator 5*′*-3*′ *exonuclease stop assay*. ~ 6 pmol of the purified ligated products obtained from an overnight ligation reaction between CS1 and PPP-Substrate-Biot or P-Substrate-Biot were digested with 2 U of Terminator (Lucigen) in a 20 μL reaction in the presence of 1X Terminator buffer A at 30 °C for 2 h. The reaction was cleaned up using Zymo RNA Clean & Concentrator-5 kit (Zymo Research) and the RNA was eluted in 6 μL milliQ water. 10 μL of quench buffer (8 M urea, 100 mM Tris-Cl, 100 mM boric acid, 100 mM EDTA) was added to it, followed by 10% denaturing PAGE.

*RNase R 3*′*-5*′ *exonuclease stop assay*. ~ 5 pmol of the purified ligated product obtained from an overnight ligation reaction between CS1 and P-Substrate-Biot was digested with 20 U of RNase R (Lucigen) in a 10 μL reaction in the presence of 1X RNase R buffer at 50 °C for 1 h. The reaction was quenched by adding 0.3 μL of 0.5 M EDTA and the enzyme was heat-inactivated at 95 °C for 3 min. 10 μL of quench buffer (8 M urea, 100 mM Tris-Cl, 100 mM boric acid, 100 mM EDTA) was added to the reaction, followed by 20% denaturing PAGE.

### Secondary structure determination by SHAPE probing

SHAPE probing was performed using a protocol reported in Walton et al., 2020^12^. Briefly, 100 pmol of the RNA construct containing 5′ and 3′ SHAPE cassettes (see Supplementary Data Table 1) was folded in 100 mM Tris-HCl, pH 8, 250 mM NaCl, 10 mM Mg^2+^ and divided into ‘modification′ and ‘control′ tubes. ~40 mM of the SHAPE reagent, 1M7 (dissolved in DMSO), was added to the ‘modification′ tube and equal volumes of DMSO were added to the ‘control′ tube. Both modified and unmodified RNAs were reverse transcribed using 40 pmol of a 5′ FAM-labeled primer (SHAPE_RT_primer; Supplementary Data Table 1) and Superscript III reverse transcriptase (Invitrogen). Reverse transcription of 30 pmol unmodified RNA in the presence of each of the four ddNTPs and 25 pmol SHAPE_RT_primer was used to generate sequencing lanes. Quenched reactions were analyzed by 10% denaturing PAGE. Normalized SHAPE reactivities were calculated by first excluding the most reactive nucleotide position and then dividing reactivities at each position by the average of the 10% most reactive positions. Normalized SHAPE reactivities were used to constrain secondary structure prediction in the RNAstructure program^50^.

### Capture and amplification of 3′-phosphorylated RNA

To demonstrate the capture of 3′-phosphorylated RNA from a mixture of cellular RNA, 0.3 μM CS1 was added to 6 μM *E. coli* tRNA mix (Millipore Sigma) that was spiked with different concentrations (0.1, 0.2, 0.3, 0.4, 0.5, 0.6 μM) of 5′ FAM-labeled RNA target (FAM-Target RNA-3′P) under standard ligation conditions. Aliquots were taken after 3 h, quenched with 9 μL of quench buffer and analyzed by 10% denaturing PAGE.

To demonstrate the capture of target RNA containing 2′-3′-cyclic phosphate, first the 2′-3′-cyclic phosphate-containing RNA (FAM-Target RNA-cP) was generated via hairpin ribozyme-mediated cleavage of FAM-HP_sub (Supplementary Data Table 1). 4 μM hairpin ribozyme, HP_ribozyme, was heated at 70 °C for 1 min followed by incubation at room temperate for 5 min. The solution was incubated at 37 °C for 15 min after adding Tris-HCl (pH 8) to a final concentration of 300 mM. This was followed by the addition of 100 mM MgCl_2_ and 2 μM FAM-HP_sub. The cleavage reaction was incubated at 37 °C for 5 h. The 5′ cleaved product containing 2′, 3′-cyclic phosphate (FAM-Target RNA-cP) was purified by 10% denaturing PAGE. Purified FAM-Target RNA-cP (42 pmol) was converted to FAM-Target RNA-3′P by adding 10 mM HCl and incubating for 3 h on ice. The RNA was then ethanol precipitated. The target capture reaction contained 2.5 pmol FAM-Target RNA-cP and 6 pmol CS1 under regular ligation conditions and was analyzed by 10% denaturing PAGE.

To demonstrate the efficient amplification of captured 3′-phosphorylated RNA, the ligated product obtained from a reaction between CS1 and Substrate-3′P was incubated with 10 U shrimp alkaline phosphatase (SAP; New England Biolabs) at 37 °C for 30 min, followed by heat inactivation at 65 °C for 20 min. The inactivated SAP was removed by Zymo RNA Clean & Concentrator-5 kit. The SAP-treated ligated product was reversed transcribed in a 20 μL reaction containing 10 μM RT primer, 2 mM dNTP mix, 2 mM DTT, 1X Protoscript buffer, and 5 U/μL Protoscript II reverse transcriptase, incubated at 37 °C for 3 h. Ligated product that was not treated with SAP was also reverse transcribed as a control. The reverse transcriptase was heat inactivated at 80 °C for 5 min and the cDNAs were purified with QIAquick PCR purification kit. The purified product was directly used as input for a PCR with primers, PCR_LigFwd_primer and PCR_Rvs_primer (Supplementary Data Table 1). The PCR cycle was: 1) 95 °C for 2 min; 2) 16 cycles of 94 °C for 30 s, 54 °C for 1 min., and 72 °C for 1 min.; 3) 72 °C for 10 min. The PCR product was purified using QIAquick PCR purification kit and analyzed by 10% denaturing PAGE.

### Reporting summary

Further information on research design is available in the Nature Portfolio Reporting Summary linked to this article.

## Supplementary information

Supplementary Information

Description of Additional Supplementary Files

Supplementary Data

Reporting Summary

Transparent Peer Review file

## Source data

Source Data

## Data Availability

The data supporting the findings of this study are available from the corresponding authors upon request. The main data supporting the findings of this study are available within the article and RNA/DNA sequences used in this work are provided in a Supplementary Data Table 1. The Source Data file includes source data underlying Figs. 2–5, Supplementary Figs. 2–10, and 12-16. This file contains data used for all plots and secondary structure determination by SHAPE probing. It also contains uncropped gels corresponding to all gels presented in this paper. Raw sequencing data obtained from the in vitro evolution relevant to this work can be accessed at CurateND (10.7274/30810947). Source data are provided with this paper.

## Code availability

The codes used for pre-processing and analyzing sequencing data can be freely accessed on the lab′s GitHub (https://github.com/dasguptalab/Directed-evolution-from-PI-to-PPP-Ligases)^51^.
