# Supplementary material for: A ribozyme ligase that requires a 3′ terminal phosphate on its RNA substrate
Source: Nat Commun. 2026 Jul 13;17:5634. doi: 10.1038/s41467-026-74622-8 (PMC13365474; doi:10.1038/s41467-026-74622-8)
Supplement: Supplementary file 1 — Supplementary Information [file 41467_2026_74622_MOESM1_ESM.pdf]

Supplementary Information

A ribozyme ligase that requires a 3′ terminal phosphate on its RNA substrate

Annyesha Biswas[a], Zoe Weiss[b], Jack W. Szostak[c,d,#], and Saurja DasGupta[a,e*]

[a] Department of Chemistry and Biochemistry, University of Notre Dame, Notre Dame, IN 46556, USA
[b] Harvard/Massachusetts Institute of Technology MD-PhD Program, Harvard Medical School, Boston, MA 02115, USA
[c] Howard Hughes Medical Institute, The University of Chicago, Chicago, IL 60637, USA
[d] Department of Chemistry, The University of Chicago, Chicago, IL 60637, USA
[e] Department of Biological Sciences, University of Notre Dame, Notre Dame, IN 46556, USA

Correspondence should be addressed to:
[#] Email: jwszostak@uchicago.edu
[*] Email: sdasgupta@nd.edu

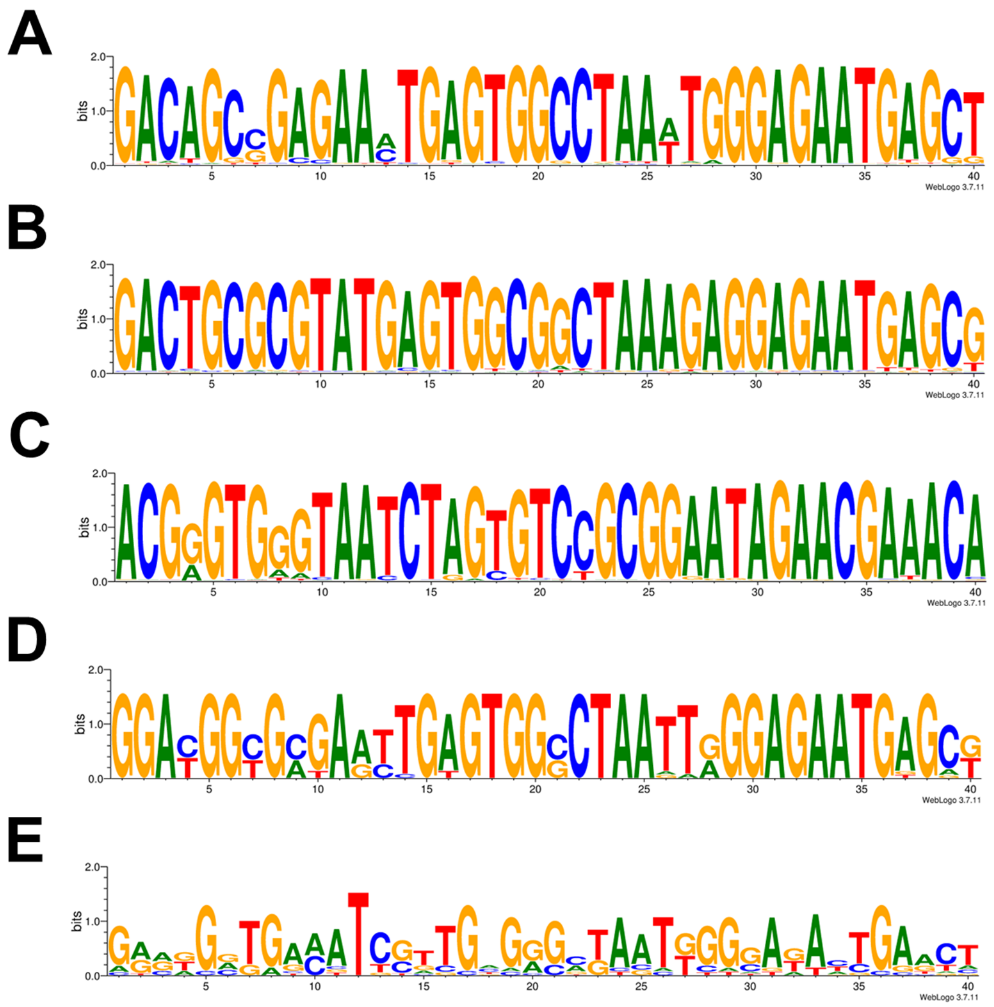

Supplementary Fig. 1. Sequence logos of the variable region in clusters 1-5 (A-E). The sequence logos show the consensus sequence for the 40-nt variable region and depict the relative abundances of each nucleotide for each position. The sequence logo was generated by the WebLogo online server (https://weblogo.berkeley.edu/logo.cgi).

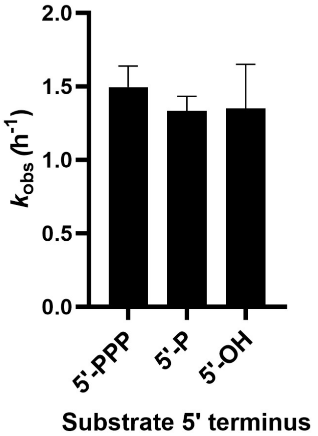

Supplementary Fig. 2. CS1-catalyzed ligation reactions with RNA substrates containing 5′-triphosphate (5′-PPP), 5′-monophosphate (5′-P), or 5′-hydroxyl (5′-OH) groups are comparable. Ligation rates exhibited by CS1, the most abundant ligase isolated from the selection, are similar with substrates containing 5′-PPP, 5′-P, or 5′-OH groups. Data were obtained from triplicate measurements, where error bars indicate standard error of the mean (S.E.M). Ligation reactions contained 1 μM ribozyme CS1 and 2 μM Substrate-Biot in 100 mM Tris-HCl (pH 8.0), 300 mM NaCl, and 100 mM $MgCl_2$. Source data are provided as a Source Data file.

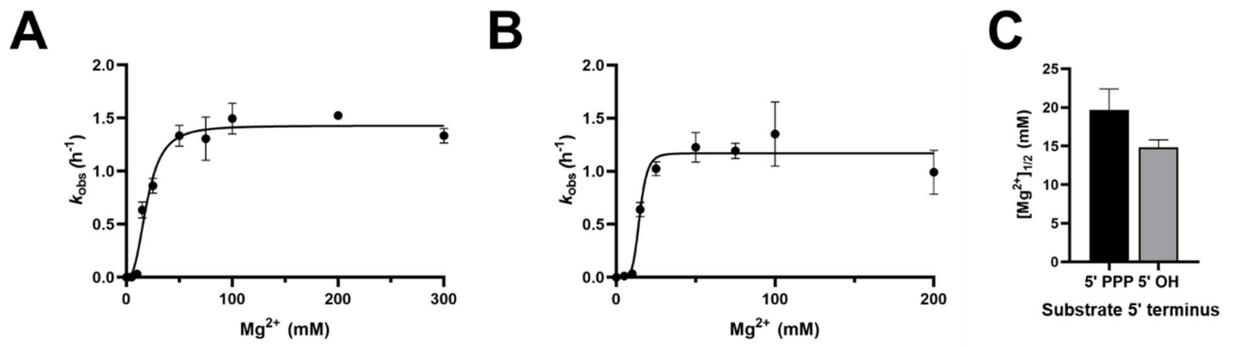

Supplementary Fig. 3. Comparable effect of Mg^{2+} concentration on the rates of CS1-catalyzed ligation of RNA substrates containing 5′-triphosphate (5′-PPP) and 5′-hydroxyl (5′-OH) groups. A. k_{obs} values of CS1 with a PPP-Substrate-Biot increase steeply till $[Mg^{2+}]$ reaches 50 mM, and plateaus at higher concentrations. **B.** k_{obs} values with OH-Substrate-Biot increase steeply till $[Mg^{2+}]$ reaches 25 mM and plateaus at higher concentrations. **C**. Mg^{2+} titration data are fitted to the Hill Equation and yield $[Mg^{2+}]_{1/2}$ values of 19.7 ± 2.70 mM and 14.8 ± 2.98 mM for PPP-Substrate-Biot and OH-Substrate-Biot, respectively. Data were obtained from triplicate measurements, where error bars indicate standard error of the mean (S.E.M). Ligation reactions contained 1 μM ribozyme and 2 μM PPP-Substrate-Biot or OH-Substrate-Biot in 100 mM Tris-HCl (pH 8.0), 300 mM NaCl, and the indicated amounts of $MgCl_2$. Source data are provided as a Source Data file.

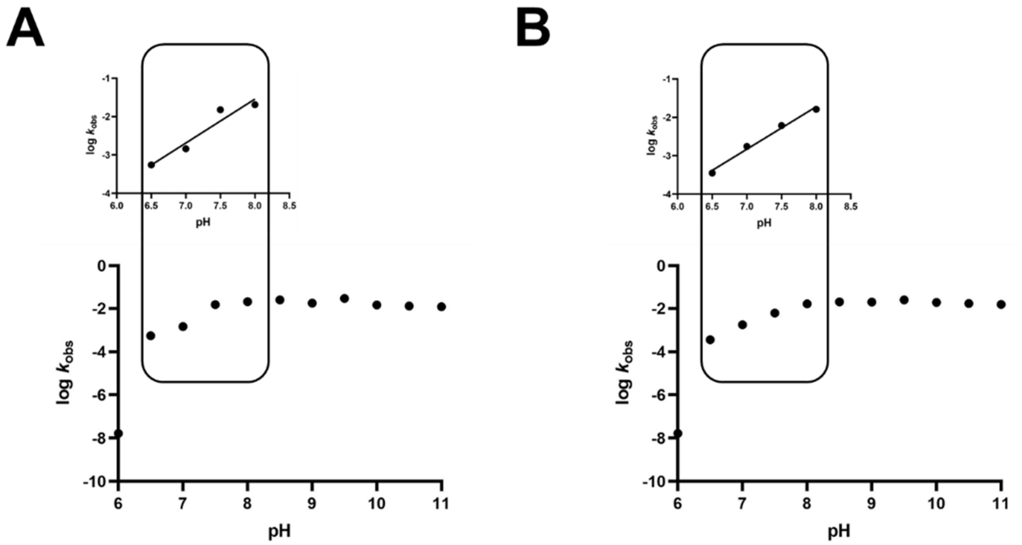

Supplementary Fig. 4. Comparable effect of pH on the rates of CS1-catalyzed ligation of RNA substrates containing 5′-monophosphate (5′-P) and 5′-hydroxyl (5′-OH) groups. Log k_{obs} values increase till pH 8 and then remain approximately constant. The inset shows that the increase in ligation rate is log-linear in the range pH 6.5-8 with a slope of ~1 with both P-Substrate-Biot (A) and OH-Substrate-Biot (B). Ligation reactions contained 1 μM ribozyme and 2 μM P-Substrate-Biot or OH-Substrate-Biot, 300 mM NaCl, and 100 mM $MgCl_2$ at the indicated pH values. The following buffers were used: MES: pH 6.0, 6.5; Tris: pH 7.0, 7.5, 8.0, 8.5, 9.0; Bis-tris propane: pH 9.5; CAPS: 10, 10.5, 11. Experiments to determine k_{obs} at each pH were performed in triplicate. Source data are provided as a Source Data file.

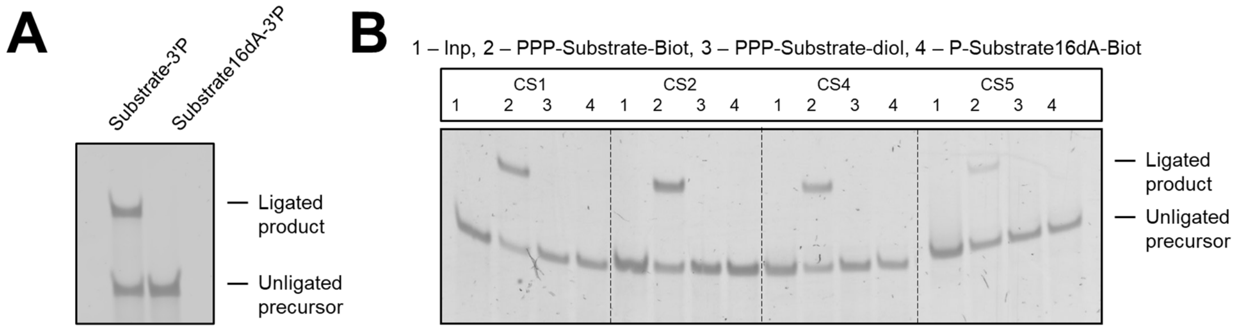

Supplementary Fig. 5. The reactivity of ribozymes toward RNA substrates with different 3′ termini. A. CS1-catalyzed ligation with 3′-phosphorylated substrates involves the substrate terminal 2′ hydroxyl group; 2′ deoxy substrates are inert to ligation. **B.** CS1, CS2, CS4, and CS5 react with substrates that possess a 3′-TEG-biotin group but are inert toward substrates containing a terminal diol or terminating in a deoxynucleotide (dA). Lane 1 (Inp) is the input lane that contains only ribozyme. Ligation reactions contained 1 μM ribozyme and 2 μM RNA substrate in 100 mM Tris-HCl (pH 8.0), 300 mM NaCl, and 100 mM $MgCl_2$. Ligation reactions were assayed at 3 h. Experiments were performed in triplicate. Source data are provided as a Source Data file.

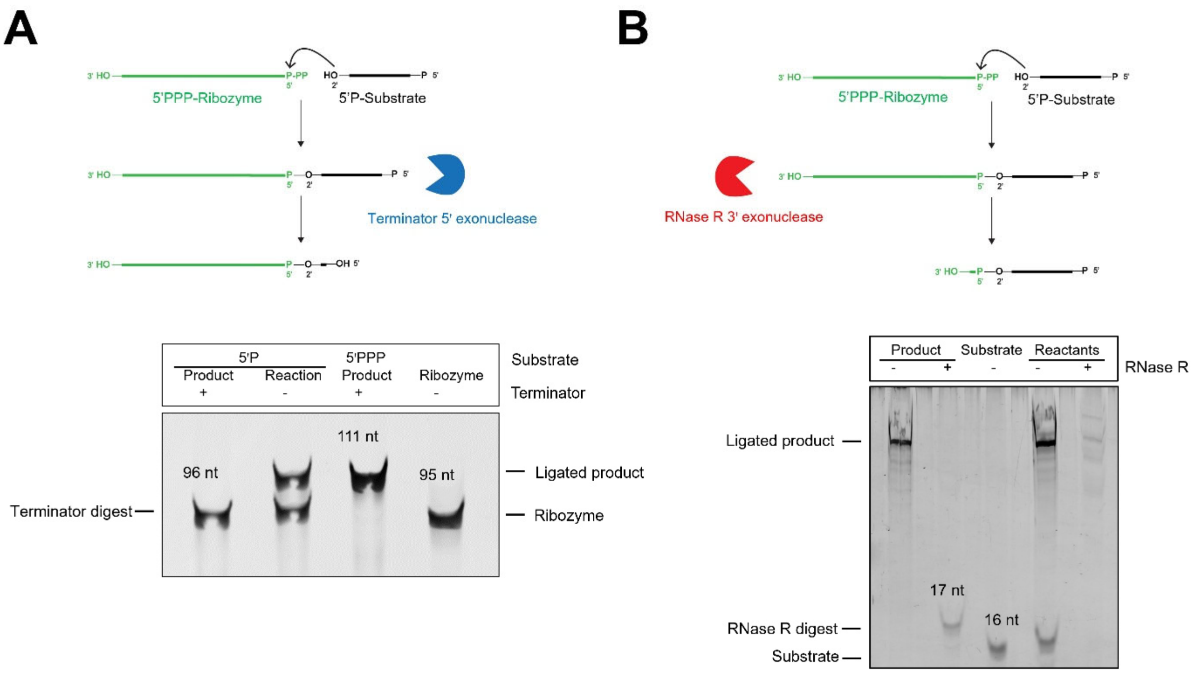

Supplementary Fig. 6. Ligation creates a 2′-5′ phosphodiester bond between the substrate and the ribozyme. The lengths of the partially digested products from separate 5′→3′ and 3′→5′ exonuclease digestions support the presence of a noncanonical 2′-5′ linkage between the substrate and the ribozyme. **A.** The purified ligated product from the reaction between CS1 and P-Substrate-Biot is partially degraded by the 5′→3′ exonuclease, Terminator, to generate a digested product that is 1 nt longer than the ribozyme. The corresponding ligated product with PPP-Substrate-Biot is unreactive to Terminator degradation, serving as a negative control. **B.** The purified ligated product from the reaction between CS1 and P-Substrate-Biot is almost completely degraded by the 3′→5′ exonuclease, RNase R, to generate a digested product that is 1 nt longer than the substrate. A pre-reaction mixture of CS1 and the substrate, on the other hand, is completely degraded by RNase R. The 3′-TEG-Biot groups are not shown. Exonuclease digestions were carried out according to vendor specifications (See ‘Confirmation of a 2′-5′ phosphodiester bond in the ligated product’ in Methods). Experiments were performed in triplicate. Source data are provided as a Source Data file.

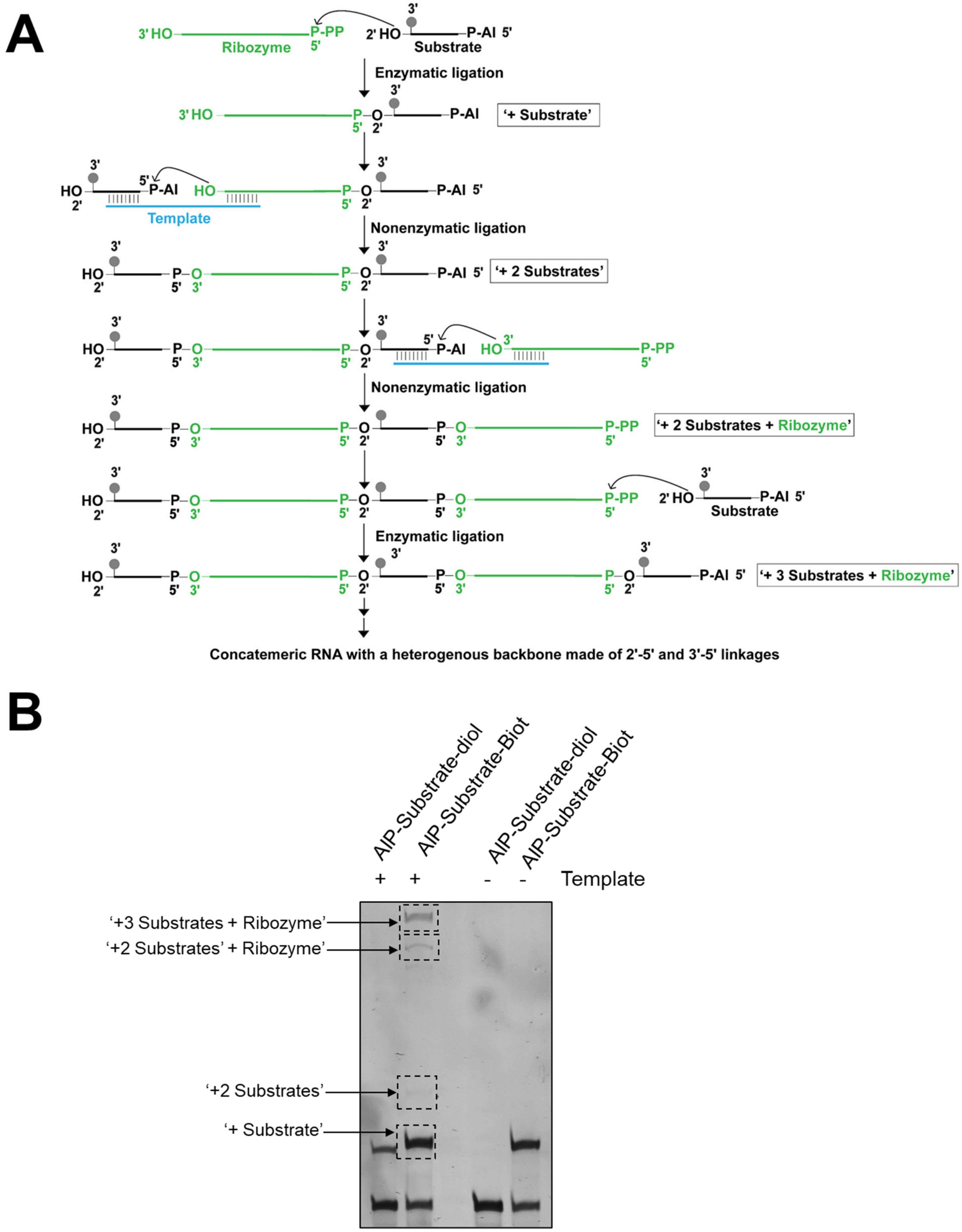
A
3' HO
Ribozyme
P-PP
5'
2' HO
3'
Substrate
P-AI 5'
Enzymatic ligation
'+ Substrate'
Template
Nonenzymatic ligation
'+ 2 Substrates'
Nonenzymatic ligation
'+ 2 Substrates + Ribozyme'
Enzymatic ligation
'+ 3 Substrates + Ribozyme'
Concatemeric RNA with a heterogenous backbone made of 2'-5' and 3'-5' linkages
B
AIP-Substrate-diol
AIP-Substrate-Biot
AIP-Substrate-diol
AIP-Substrate-Biot
+ + - - Template
'+3 Substrates + Ribozyme'
'+2 Substrates' + Ribozyme'
'+2 Substrates'
'+ Substrate'

Supplementary Fig. 7. Ligation with 5′ 2-aminoimidazole-activated, 3′ biotinylated substrates in the presence of an external template produces concatemeric RNA containing both 2′-5′ and 3′-5′ linkages. A. A combination of ribozyme-catalyzed and templated-nonenzymatic ligation generates concatemeric RNA. The gray circle represents the TEG-Biotin moiety. **B.** Higher-order bands corresponding to concatemeric ligation products are observed when CS1 is incubated with AIP-Substrate-Biot in the presence of a template at 100 mM Mg^{2+}. These bands are not observed in the absence of a template because detectable nonenzymatic ligation requires a template. Using an AIP-substrate without TEG-biotin also fails to generate higher-order products because the ribozyme does not ligate RNAs with terminal diols. The '+substrate' band observed with AIP-Substrate-diol in the presence of a template is due to nonenzymatic ligation. Ligation reactions contained 1 μM ribozyme, 1.2 μM RNA template, and 2 μM RNA substrate in 100 mM Tris-HCl (pH 8.0), 300 mM NaCl, and 100 mM $MgCl_2$. Ligation reactions were assayed at 3 h. Experiments were performed in triplicate. Source data are provided as a Source Data file.

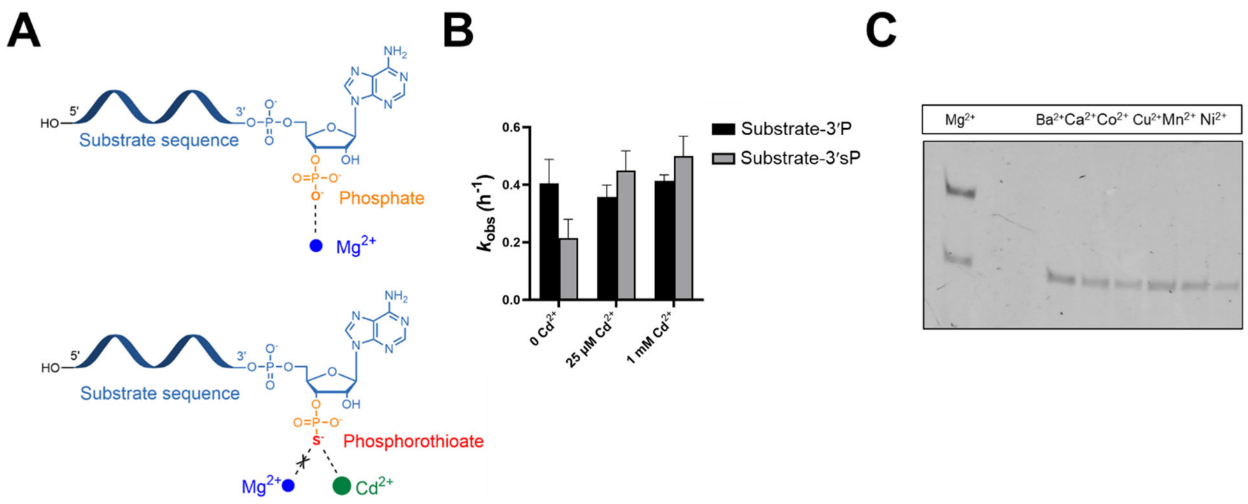

Supplementary Fig. 8. Mg^{2+} plays a potential catalytic role in ligation. A. Schematic illustrating a phosphorothioate metal rescue experiment. If the interaction between Mg^{2+} and a phosphate O is important for catalysis, replacing the O atom with a S atom (Substrate-3′sP), which weakens this interaction, is expected to cause a catalytic defect. Supplementing the reaction with thiophilic Cd^{2+} restores the putative interaction and is expected to rescue activity. **B.** A substrate with a 3′-thiophosphate causes a 2-fold reduction in ligation rate, which was rescued upon Cd^{2+} supplementation with an overall metal rescue of ~2.5. Kinetic data were obtained from triplicate measurements, where error bars indicate standard error of the mean (S.E.M). **C.** Only Mg^{2+}, among the divalent cations tested, supported ligation, suggesting a catalytic role. Ligation reactions contained 1 μM ribozyme and 2 μM substrate in 100 mM Tris-HCl (pH 8.0), 300 mM NaCl, 50 mM $MgCl_2$ (B) or 100 mM $MgCl_2$ (C), and the indicated amounts of Cd^{2+} (B) or 100 mM of the indicated divalent cations instead of Mg^{2+} (C). Ligation reactions with different metal ions were assayed at 3 h. Source data are provided as a Source Data file.

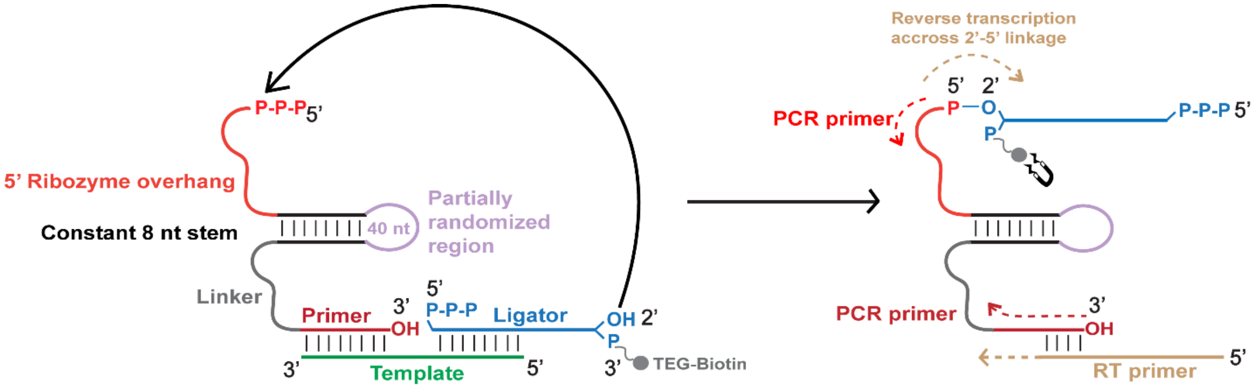

Supplementary Fig. 9. The unexpected ligation pathway exhibited by the isolated ribozymes. The ribozymes reported in this work catalyze ligation between the substrate 2′-hydroxyl and the ribozyme 5′-triphosphate groups. The 4-nt overlap between the RT primer (gold) and 3′ end of the ribozyme ('primer', red) allowed ligated products that were separated by streptavidin capture to be reverse transcribed. PCR primers targeting the 5′ and 3′ ends of the ribozyme amplified the ribozyme sequences to produce dsDNA as the template for *in vitro* transcription, which was used to generate the selection library for the subsequent round.

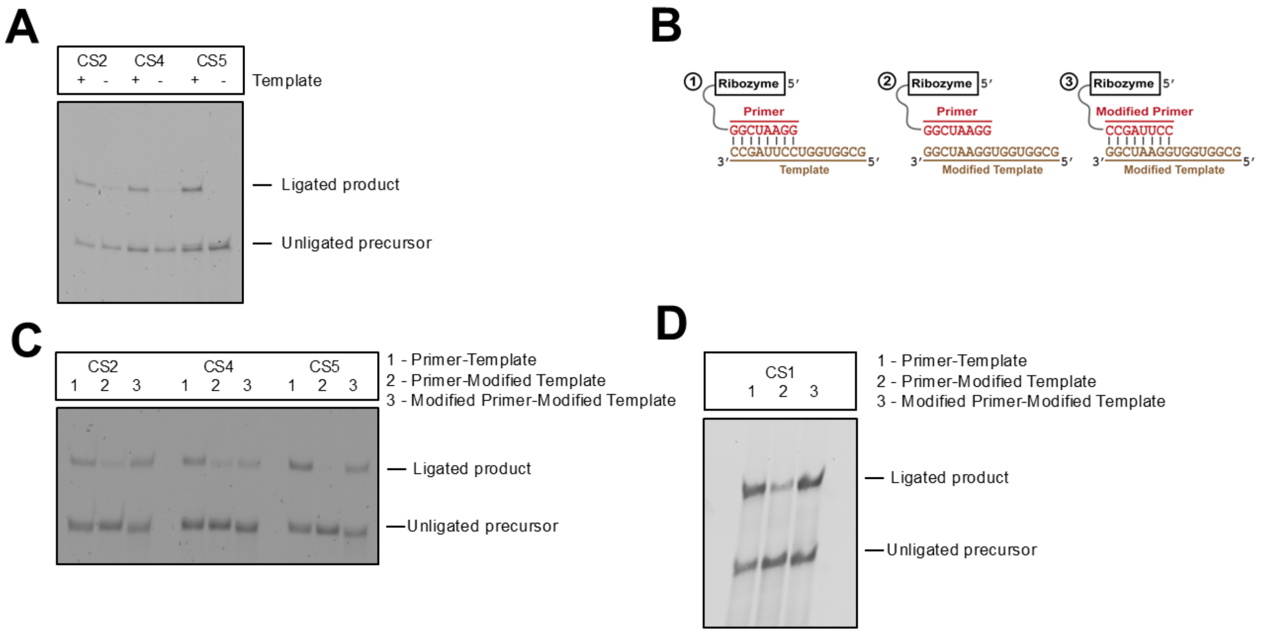

Supplementary Fig. 10. The role of template in ligation catalyzed by CS1, CS2, CS4, and CS5. A. CS2, CS4, and CS5 require an external template for ligation. **B.** Schematic for the compensatory mutational rescue of ligase activity in CS2, CS4, and CS5. **C.** Base-pairing interaction between the template and the 3′-primer sequence of the ribozyme enhances ligation. **D.** Base-pairing interactions between the template and the 3′-primer sequence of CS1 stimulate ligation. Disrupting this interaction by mutations in the template diminishes ligation; however, compensatory mutations in the 3′-primer sequence of the ribozymes rescue ligation. Ligation reactions contained 1 μM ribozyme, 1.2 μM RNA template, and 2 μM Substrate-3′P in 100 mM Tris-HCl (pH 8.0), 300 mM NaCl, and 100 mM $MgCl_2$. Ligation reactions were assayed at 3 h. Experiments were performed in triplicate. Source data are provided as a Source Data file.

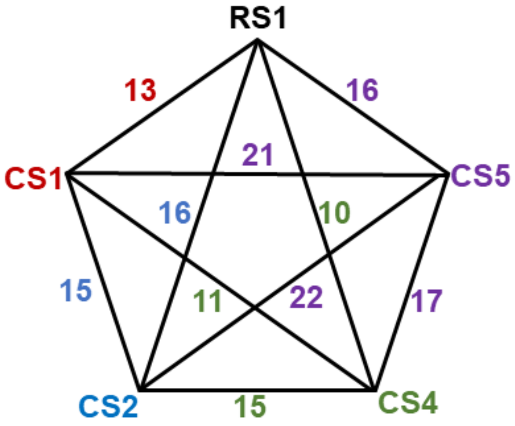

Supplementary Fig. 11. Sequence divergence of the ribozymes. CS1, CS2, CS4, and CS5 ribozymes are 10-16 mutations from the parent AIP-Ligase, RS1, and diverge by 11-22 mutations from each other (See Table 1). These numbers change when the sequences are aligned by considering insertion/deletions as shown in Supplementary Fig. 12**.**

```
RS1: GACUCACUGACACAGAUCCACUCACGGACAGCG-GAAUGCUGCCA--ACCGUGCG-GG--CUAAUUG-GCAGACUGAGCUCGCUGUCCUUUUUUGGCUAAGG

CS1: GACUCACUGACACAGAUCCACUCACGGACAGCG-GACAGCCGAGA--AAUGAGUG-GC--CUAAAUG-GGAGAAUGAGCUCGCUGUCCUUUUUUGGCUAAGG
CS2: GACUCACUGACACAGAUCCACUCACGGACAGCG-GACUGC-GCGU--A-UGAGUG-GCGGCUAAA-GAGGAGAAUGAGCGCGCUGUCCUUUUUUGGCUAAGG
CS4: GACUCACUGACACAGAUCCACUCACGGACAGCGGGAUGGU-GCGA--ACUGAGUG-GG--CUAAUU-AGGAGAAUGAGCGCGCUGUCCUUUUUUGGCUAAGG
CS5: GACUCACUGACACAGAUCCACUCACGGACAGCGGGAGGGU-GACAUCGUUGAGAGAG------AA-UGGGGAUAUUGAACUCGCUGUCCUUUUUUGGCUAAGG
```

Supplementary Fig. 12. Sequence alignment of the isolated ribozymes by considering insertions/deletions. Putative base-paired stems are underlined and colored. Nucleotides reactive to SHAPE probing are shown in bold and depicted in orange or red, where red indicates a normalized SHAPE reactivity of >0.85 and orange indicates a normalized SHAPE reactivity of 0.4-0.85 (See Supplementary Fig. 13). Source data are provided as a Source Data file.

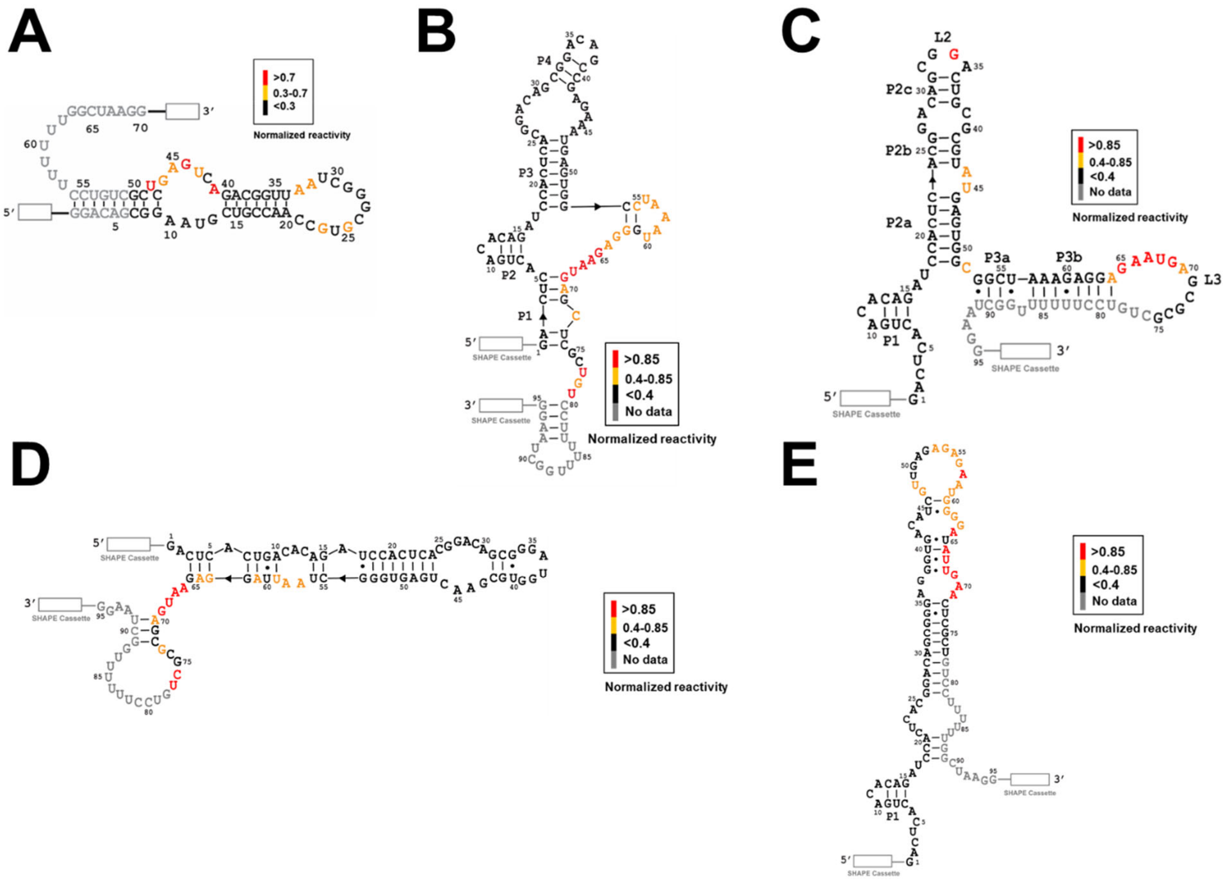

Supplementary Fig. 13. SHAPE-derived secondary structures of CS1, CS2, CS4, and CS5. Secondary structures of (B) CS1, (C) CS2, (C) CS4, and (D) CS5 determined by the RNAstructure program[1] using reactivity constraints obtained from SHAPE experiments. The SHAPE-derived secondary structure of the (A) parental AIP-Ligase, RS1 given as reference.[2] 5′ and 3′ SHAPE cassettes are denoted by white rectangles with gray borders. Nucleotides for which no data was obtained are shown in gray. Nucleotides are colored in red, orange, and black according to their normalized SHAPE reactivities, as shown in the reactivity legend. Source data are provided as a Source Data file.

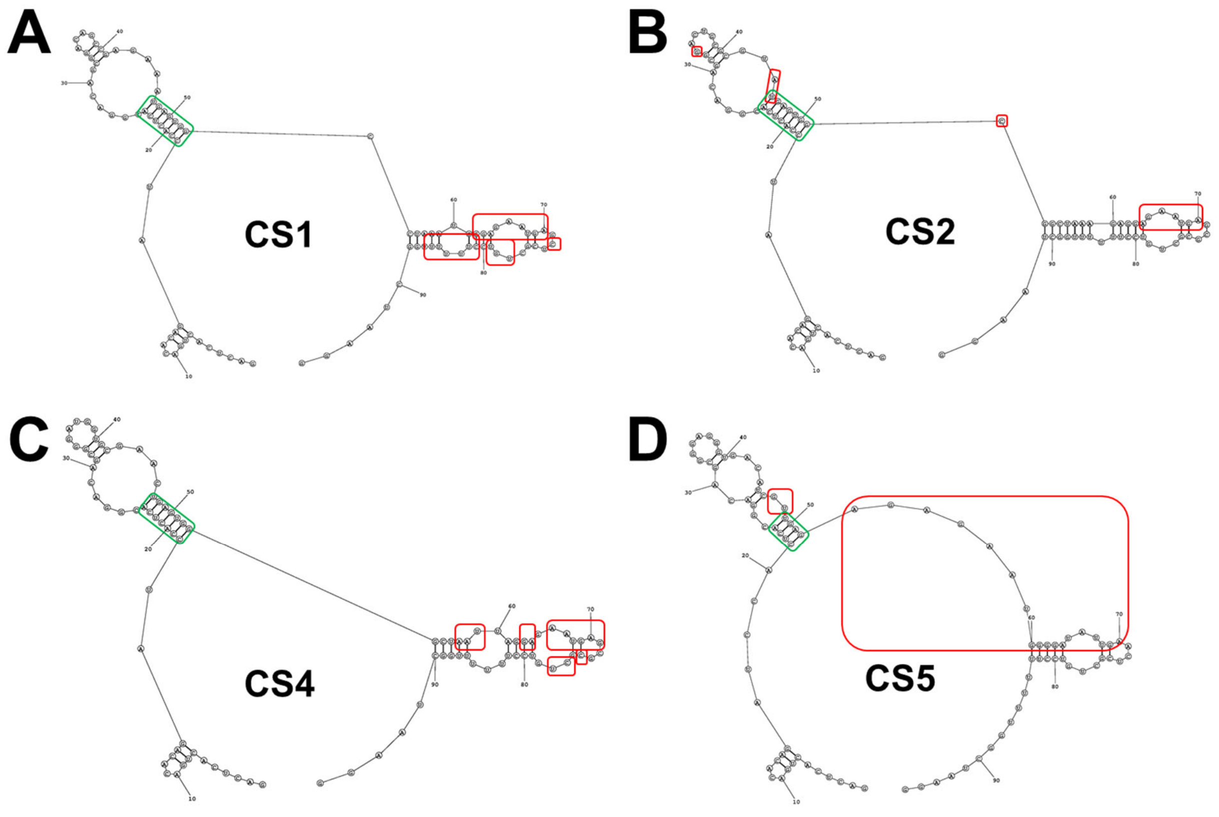

Supplementary Fig. 14. Computationally-predicted common secondary structures of CS1, CS2, CS4, and CS5. Secondary structures of (A) CS1, (B) CS2, (C) CS4, and (D) CS5 determined by the TurboFold algorithm within the RNAstructure program.[1,3] SHAPE-reactive regions (normalized reactivity of >0.4) in each RNA are highlighted in red boxes (See Supplementary Figs. 12 and 13). The base-paired stems composed of 5′-CCACUCA-3′ and 3′-GGUGAGU-5′ regions common to CS1, CS2, and CS4 in TurboFold-predicted and SHAPE-derived structures are highlighted in green boxes. A shorter version of this stem composed of 5′-CUCA-3′ and 3′-GAGU-5′ is featured in the TurboFold-predicted structure (but not in the SHAPE-derived structure) of CS5 is also highlighted in a green box. The predicted structures of CS1, CS2, and CS4 converge into an overall common fold, whereas CS5 appears to assume a different fold.

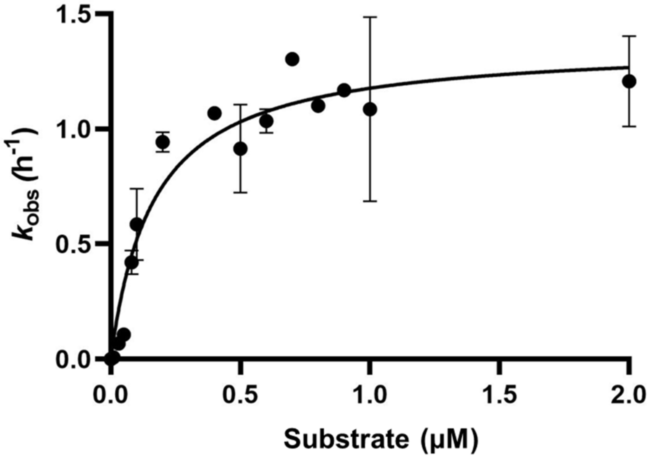

Supplementary Fig. 15. The effect of substrate concentration on ligation rate constant for CS1-catalyzed ligation. Michaelis-Menten plot for CS1-catalyzed ligation with Substrate-3′P reveals a K_M value of 0.1649 ± 0.042 μM and a k_{cat}/K_M value (catalytic efficiency) of 27.65 μM^{-1} h^{-1}. Ligation reactions contained 0.3 μM ribozyme and the indicated amounts of Substrate-3′P in 100 mM Tris-HCl (pH 8.0), 300 mM NaCl, and 100 mM $MgCl_2$. Experiments were performed in triplicate. Source data are provided as a Source Data file.

A

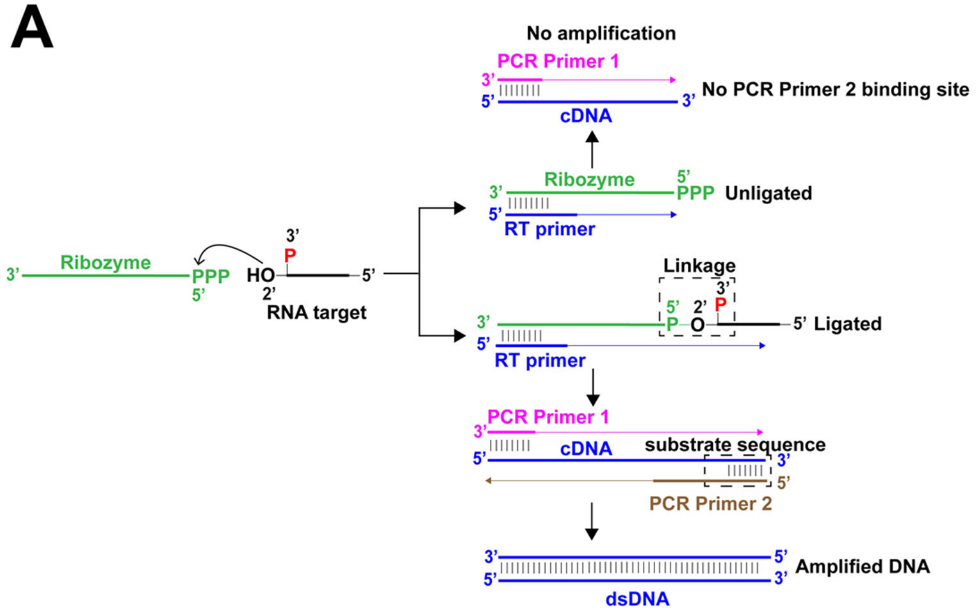

B

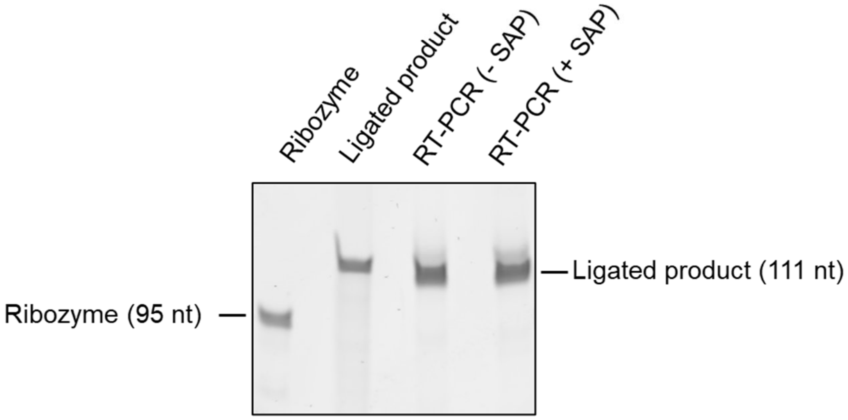

Supplementary Fig. 16. Ribozyme-assisted capture and amplification of 3′-phosphorylated RNA. **A.** Schematic illustrating the reverse transcription and PCR amplification of the ligated product generated by a reaction between CS1 and Substrate-3′P. **B.** Detection of a full-length dsDNA product after RT-PCR amplification of the ligated product in the absence of phosphatase treatment (-SAP) indicates successful reverse transcription across the unique 2′-5′, 3′P linkage between the ribozyme and the substrate. The SAP reaction was performed according to vendor specifications (See ‘Capture and amplification of 3′-phosphorylated RNA’ in Methods). Experiments were performed in triplicate. Source data are provided as a Source Data file.

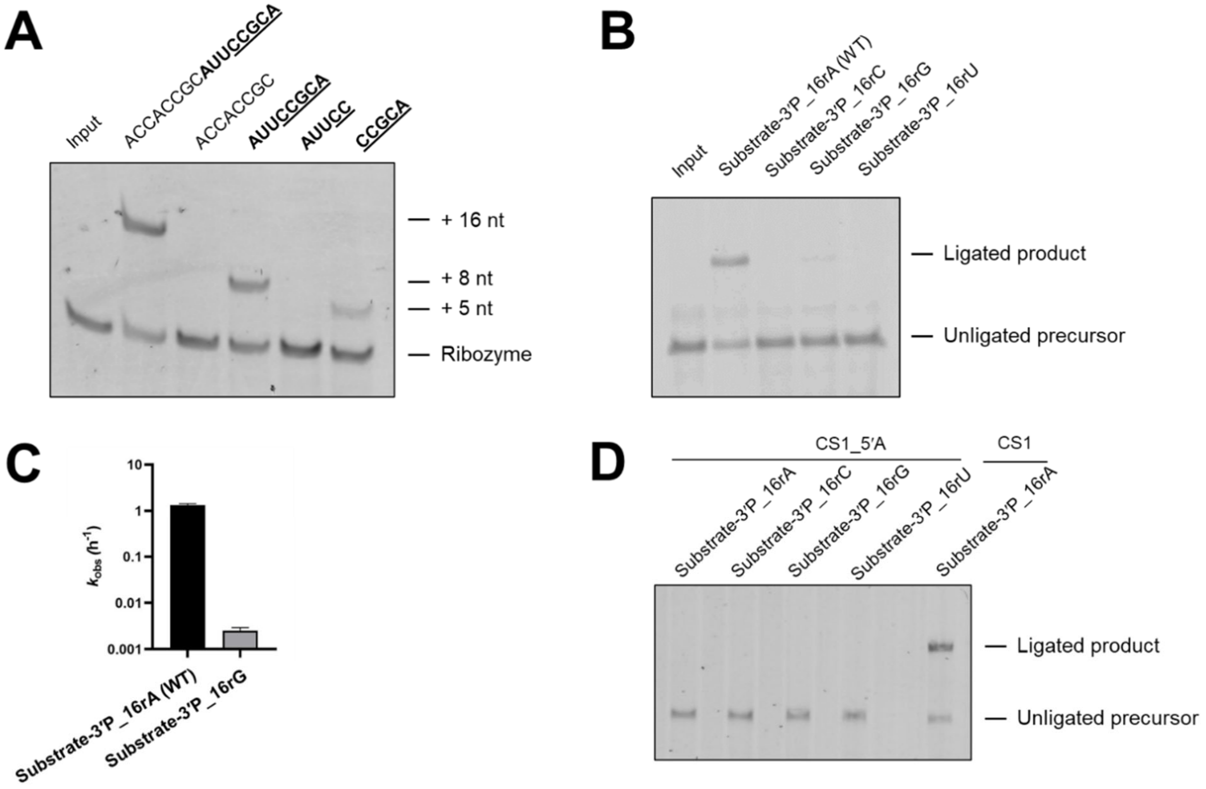

Supplementary Fig. 17. Substrate scope of ribozyme-catalyzed RNA ligation. **A**. 3′-phosphorylated RNA substrates of lengths ranging from 5-16 nt were active for ligation by CS1; however, only substrates containing the sequence CCGCA were ligated. **B.** The terminal adenine (the residue that contains the nucleophilic 2′-OH group) of the substrate is important for ligation. Substrates with terminal uridine or cytidine were inactive. Ligation with a substrate containing a terminal guanine was detectable but inefficient. **C.** Ligation was ~500-fold slower with a substrate containing a terminal guanine relative to that with a substrate containing a terminal adenine. Data were obtained from triplicate measurements, where error bars indicate standard error of the mean (S.E.M). **D.** The 5′ terminal guanine (the residue that contains the electrophilic 5′-PPP group) of CS1 is indispensable for ligation. Ligation reactions contained 1 μM ribozyme and 2 μM substrate in 100 mM Tris-HCl (pH 8.0), 300 mM NaCl, and 100 mM $MgCl_2$. Ligation reactions were assayed at 3 h. Experiments were performed in triplicate. Source data are provided as a Source Data file.

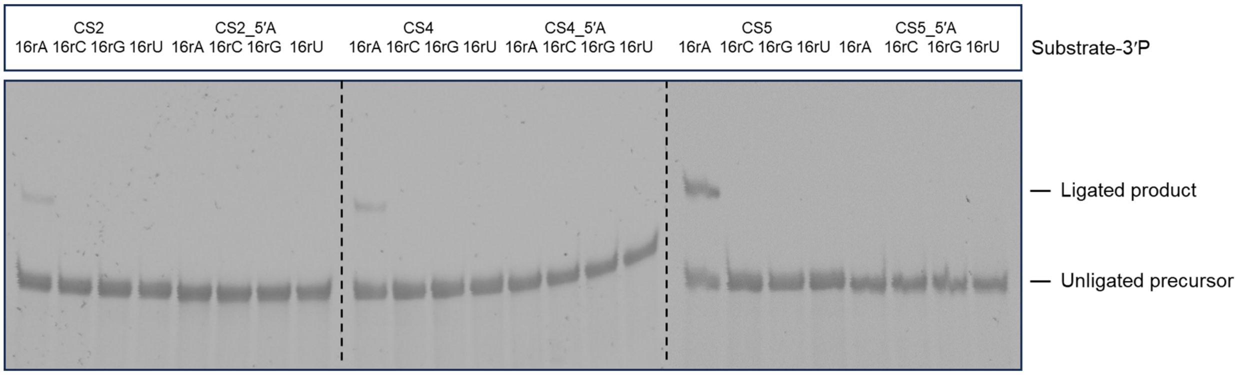

Supplementary Fig. 18. Sequence requirements for the ribozyme and substrate at the ligation junction for CS2, CS4, and CS5. The 5′ terminal guanine (the residue that contains the electrophilic 5′-triphosphate group) of CS2, CS4, and CS5 and the terminal adenine (the residue that contains the nucleophilic 2′-hydroxyl group) of the substrate are essential for ligation. Ligation reactions contained 1 μM ribozyme, 1.2 μM template, and 2 μM substrate in 100 mM Tris-HCl (pH 8.0), 300 mM NaCl, and 100 mM $MgCl_2$. Ligation reactions were assayed at 3 h. Experiments were performed in triplicate. Source data are provided as a Source Data file.

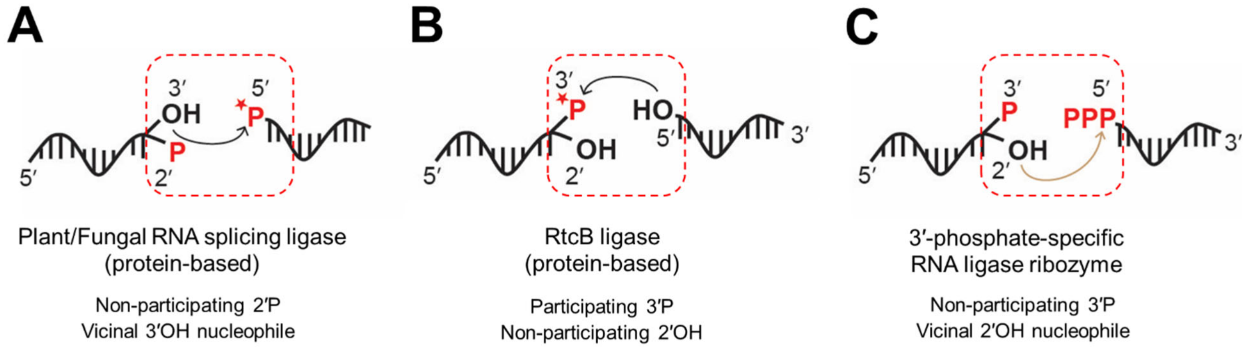

Supplementary Fig. 19. Resemblance of the evolved ribozyme reactivity with reactivities of protein-based RNA repair ligases.[4] A. The plant/fungal tRNA ligase joins 2′-phosphorylated RNAs with 5′-activated RNAs, with the 3′-hydroxyl vicinal to the 2′-phosphate acting as the nucleophile. **B.** RtcB ligase joins 3′-phosphorylated RNAs to RNAs containing 5′-hydroxyl groups, where these two groups participate in the ligation reaction. **C.** The ribozymes reported in this work ligate 3′-phosphorylated RNAs to 5′-triphosphorylated RNA, where the 2′-hydroxyl group vicinal to the 3′-phosphate on one RNA functions as the nucleophile and reacts with the 5′-triphosphate group of the other.

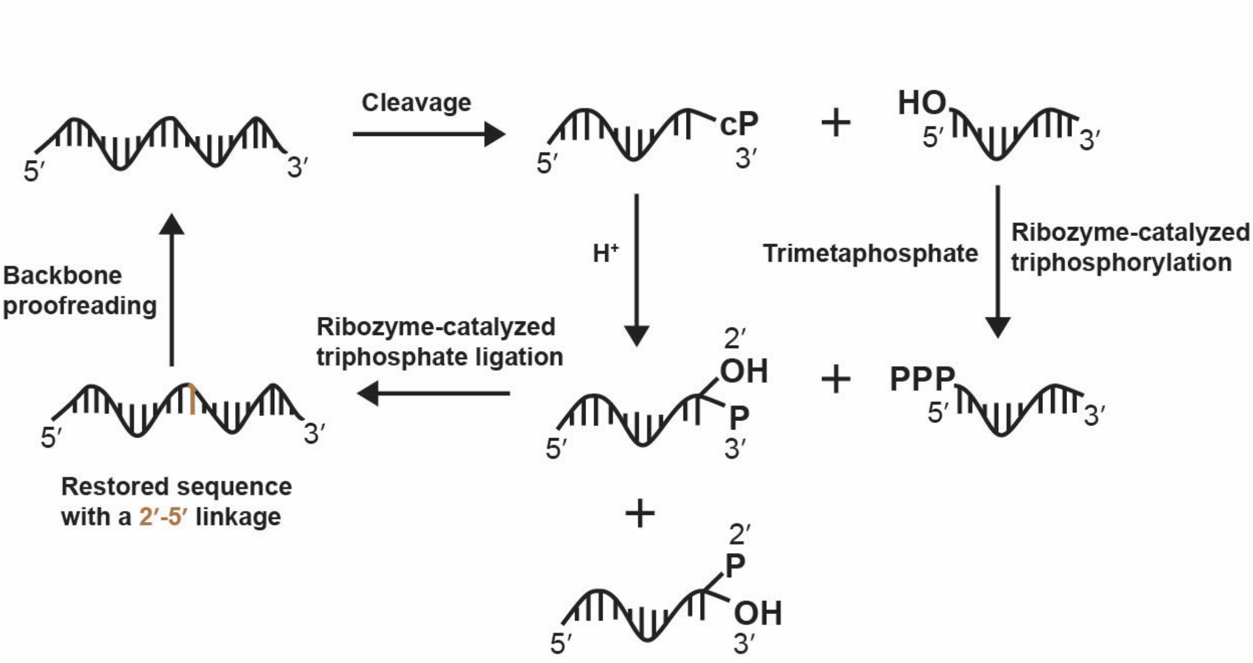

Supplementary Fig. 20. A hypothetical prebiotic pathway for ribozyme-assisted repair of RNA cleavage products. The 5′ RNA cleavage product containing a 2′, 3′-cyclic phosphate is hydrolyzed to a 2′- or 3′-phosphate. The 5′ hydroxyl group of the 3′ cleavage product is activated with a triphosphate group by a ribozyme-catalyzed reaction that uses trimetaphosphate as the phosphorylating agent.[5] A trans-acting ribozyme with the ligase activity reported in this work joins the resulting RNA oligonucleotides to regenerate the original RNA sequence pre-cleavage. This ligated product contains a 2′-5′ linkage in its backbone as opposed to an all 3′-5′ phosphodiester backbone in the original RNA. 2′-5′ linkages are recycled nonenzymatically to 3′-5′ linkages through energy dissipative cycles.[6]

REFERENCES

1. Mathews, D. H.; Disney, M. D.; Childs, J. L.; Schroeder, S. J.; Zuker, M.; Turner, D. H. Incorporating chemical modification constraints into a dynamic programming algorithm for prediction of RNA secondary structure. *Proc Natl Acad Sci U S A* **2004**, *101* (19), 7287-7292. DOI: 10.1073/pnas.0401799101
2. Walton, T.; DasGupta, S.; Duzdevich, D.; Oh, S. S.; Szostak, J. W. In vitro selection of ribozyme ligases that use prebiotically plausible 2-aminoimidazole-activated substrates. *Proc Natl Acad Sci U S A* **2020**, *117* (11), 5741-5748. DOI: 10.1073/pnas.1914367117
3. Harmanci, A. O.; Sharma, G.; Mathews, D. H. TurboFold: iterative probabilistic estimation of secondary structures for multiple RNA sequences. *BMC Bioinformatics* **2011**, *12*, 108. DOI: 10.1186/1471-2105-12-108
4. Shuman, S. RNA Repair: Hiding in Plain Sight. *Annu Rev Genet* **2023**, *57*, 461-489. DOI: 10.1146/annurev-genet-071719-021856
5. Moretti, J. E.; Muller, U. F. A ribozyme that triphosphorylates RNA 5'-hydroxyl groups. *Nucleic Acids Res* **2014**, *42* (7), 4767-4778. DOI: 10.1093/nar/gkt1405
6. Mariani, A.; Sutherland, J. D. Non-Enzymatic RNA Backbone Proofreading through Energy-Dissipative Recycling. *Angew Chem Int Ed Engl* **2017**, *56* (23), 6563-6566. DOI: 10.1002/anie.201703169
