# Supplementary material for: A ribozyme ligase that requires a 3′ terminal phosphate on its RNA substrate
Source: Nat Commun. 2026 Jul 13;17:5634. doi: 10.1038/s41467-026-74622-8 (PMC13365474; doi:10.1038/s41467-026-74622-8)
Supplement: Supplementary file 2 — Description of Additional Supplementary Files [file 41467_2026_74622_MOESM2_ESM.pdf]

Description of Additional Supplementary Files

File name: Supplementary Data 1

Description: RNA/DNA sequences used in this work
