# Supplementary material for: A ribozyme ligase that requires a 3′ terminal phosphate on its RNA substrate
Source: Nat Commun. 2026 Jul 13;17:5634. doi: 10.1038/s41467-026-74622-8 (PMC13365474; doi:10.1038/s41467-026-74622-8)
Supplement: Supplementary file 3 — Supplementary Data [file 41467_2026_74622_MOESM3_ESM.pdf]

Supplementary Data Table

A ribozyme ligase that requires a 3′ terminal phosphate on its RNA substrate

Annyesha Biswas[a], Zoe Weiss[b], Jack W. Szostak[c,d,#], and Saurja DasGupta[a,e*]

[a] Department of Chemistry and Biochemistry, University of Notre Dame, Notre Dame, IN 46556, USA
[b] Harvard/Massachusetts Institute of Technology MD-PhD Program, Harvard Medical School, Boston, MA 02115, USA
[c] Howard Hughes Medical Institute, The University of Chicago, Chicago, IL 60637, USA
[d] Department of Chemistry, The University of Chicago, Chicago, IL 60637, USA
[e] Department of Biological Sciences, University of Notre Dame, Notre Dame, IN 46556, USA

Correspondence should be addressed to:
[#] Email: jwszostak@uchicago.edu
[*] Email: sdasgupta@nd.edu

Oligonucleotide sequences used in this work. In the ribozyme sequences, variable nucleotides are shown in blue, the T7 promoter sequence is shown in purple, the hexauridine linker is shown in italics, the 5′ terminal nucleotide possessing the triphosphate that participates in ligation is shown in bold, and 3′ primer modifications are underlined. 5′ and 3′ SHAPE cassettes are highlighted in green. Cleavage site nucleotides in the hairpin ribozyme substrate, FAM-HP_sub, are shown in bold and underlined. Oligonucleotides were either purchased from Integrated DNA Technologies (IDT) or Chemgenes, or generated enzymatically by *in vitro* transcription (IVT) of dsDNA templates. AIP-Substrate was generated by incubating the corresponding 5′ monophosphorylated RNA with EDC and 2-aminoimidazole (2AI) (See 'RNA preparation and substrate activation' in Methods).

#	Oligo Name	Sequence (5′→3′)	Type	Source
1.1	Parent AIP-Ligase (RS1)	GACUCACUGACACAGAUCCACUCACGGACAGCGGAAUGCUGCCAACCGUGCGGGCUAAUUGGCAGACUGAGCUCGCUGUCC*UUUUUU*GGCUAAGG	RNA	IVT
1.2	r0 DNA<br>(Mutagenesis at 21% for each nucleotide position: 79% WT nucleotide and 7% of the other three)<br>Note: The sequence is written according to IDT specifications	TAATACGACTCACTATA**G**ACTCACTGACACAGATCCACTCACGGACAGCG(N1:07077907)(N2:79070707)(N2)(N3:07070779)(N1)(N4:07790707)(N3)(N1)(N4)(N4)(N2)(N2)(N4)(N4)(N1)(N3)(N1)(N4)(N1)(N1)(N1)(N4)(N3)(N2)(N2)(N3)(N3)(N1)(N1)(N4)(N2)(N1)(N2)(N4)(N3)(N1)(N2)(N1)(N4)(N3)CGCTGTCC*TTTTTT*GGCTAAGG	DNA	IDT
2.1	Template	GCGGUGGUCCUUAGCC	RNA	IDT
2.2	Modified Template	GCGGUGGUGGAAUCGG	RNA	IDT
3.1	PPP-Substrate-Biot	(5′-triphosphate)-ACCACCGCAUUCCGCA -(3′-BioTEG)	RNA	Chemgenes
3.2	PPP-Substrate-diol	(5′-triphosphate)-ACCACCGCAUUCCGCA	RNA	Chemgenes
3.3	AIP- Substrate -Biot	(5′-phosphoro-2-aminoimidazole)-ACCACCGCAUUCCGCA-(3′-BioTEG)	RNA	Activation of P-Substrate-Biot
3.4	AIP-Substrate-diol	(5′-phosphoro-2-aminoimidazole)-ACCACCGCAUUCCGCA	RNA	Activation of P-Substrate-diol
3.5	P- Substrate-Biot	(5′-monophosphate)-ACCACCGCAUUCCGCA-(3′-BioTEG)	RNA	IDT
3.6	Biot-P-Substrate-diol	(5′-BioTEG)-(5′-monophosphate)-ACCACCGCAUUCCGCA	RNA	IDT
3.7	ddT-P- Substrate-Biot	(5′inverted ddT)-(5′-monophosphate)-ACCACCGCAUUCCGCA-(3′-BioTEG)	RNA	IDT
3.8	Substrate-Biot (Substrate-TEG-Biot or OH-Substrate-Biot)	(5′-hydroxyl)-ACCACCGCAUUCCGCA-(3′-BioTEG)	RNA	IDT
3.9	Substrate-noTEG-Biot	(5′-hydroxyl)-ACCACCGCAUUCCGCA-(3′-Bio)	RNA	IDT
3.10	Substrate-TEG-DesthioBiot	(5′-hydroxyl)-ACCACCGCAUUCCGCA-(3′-desBioTEG)	RNA	IDT
3.11	Substrate-3′P (Substrate-3′P_16rA)	(5′-hydroxyl)-ACCACCGCAUUCCGCA-(3′-monophosphate)	RNA	IDT
3.12	FAM-Target RNA-3′P	(5′-FAM)- ACCACCGCAUUCCGCA-(3′-monophosphate)	RNA	IDT

3.13	Substrate-3′sP	(5′-hydroxyl)-ACCACCGCAUUCCGCA-(3′-monothiophosphate)	RNA	IDT
3.14	P-Substrate16dA-Biot	(5′-monophosphate)-ACCACCGCAUUCCGCdA-(3′-BioTEG)	RNA	IDT
3.15	Substrate16dA-3′P	(5′-hydroxyl)-ACCACCGCAUUCCGCdA-(3′-monophosphate)	RNA	IDT
3.16	Substrate16rA-2′P	(5′-hydroxyl)-ACCACCGCAUUCCGCA-(2′-monophosphate)	RNA	Chemgenes
3.17	Substrate-3′P_8mer1	(5′-hydroxyl)-ACCACCGC-(3′-monophosphate)	RNA	IDT
3.18	Substrate-3′P_8mer2	(5′-hydroxyl)-AUUCCGCA-(3′-monophosphate)	RNA	IDT
3.19	Substrate-3′P_5mer1	(5′-hydroxyl)-AUUCC-(3′-monophosphate)	RNA	IDT
3.20	Substrate-3′P_5mer2	(5′-hydroxyl)-CCGCA-(3′-monophosphate)	RNA	IDT
3.21	Substrate-3′P_16rC	(5′-hydroxyl)-ACCACCGCAUUCCGCC-(3′-monophosphate)	RNA	IDT
3.22	Substrate-3′P_16rG	(5′-hydroxyl)-ACCACCGCAUUCCGCG-(3′-monophosphate)	RNA	IDT
3.23	Substrate-3′P_16rU	(5′-hydroxyl)-ACCACCGCAUUCCGCU-(3′-monophosphate)	RNA	IDT
4.1	RT primer	GTGCGGAATGCGGTGGTCCTT	DNA	IDT
4.2	SHAPE_RT_primer	(5′-FAM)-GAACCGGACCGAAGCCCG	DNA	IDT
5.1	PCR_Fwd_primer	TAATACGACTCACTATAGACTCACTGACAC	DNA	IDT
5.2	PCR_LigFwd_primer	ACCACCGCATTCCG	DNA	IDT
5.3	PCR_Rvs_primer	mCmCTTAGCCAAAAAAGGACAGCG	DNA	IDT
6.1	CS1	**G**ACUCACUGACACAGAUCCACUCACGGACAGCGGACAGCCGAGAAAUGAGUGGCCUAAAUGGGAGAAUGAGCUCGCUGUCC*UUUUUU*GGCUAAGG	RNA	IVT
6.2	CS1_5′A	**A**ACUCACUGACACAGAUCCACUCACGGACAGCGGACAGCCGAGAAAUGAGUGGCCUAAAUGGGAGAAUGAGCUCGCUGUCC*UUUUUU*GGCUAAGG	RNA	IVT
6.3	CS1_5′ truncated	GGACAGCGGACAGCCGAGAAAUGAGUGGCCUAAAUGGGAGAAUGAGCUCGCUGUCC*UUUUUU*GGCUAAGG	RNA	IVT
6.4	CS1_3′ truncated	**G**ACUCACUGACACAGAUCCACUCACGGACAGCGGACAGCCGAGAAAUGAGUGGCCUAAAUGGGAGAAUGAGCUCGCUGUCC	RNA	IVT
6.5	CS1_5′+3′ truncated	GGACAGCGGACAGCCGAGAAAUGAGUGGCCUAAAUGGGAGAAUGAGCUCGCUGUCC	RNA	IVT
6.6	CS1_modified primer	**G**ACUCACUGACACAGAUCCACUCACGGACAGCGGACAGCCGAGAAAUGAGUGGCCUAAAUGGGAGAAUGAGCUCGCUGUCC*UUUUUU*CCGAUUCC	RNA	IDT
6.7	CS1_SHAPE	GGCCUUCGGGCCAA**G**ACUCACUGACACAGAUCCACUCACGGACAGCGGACAGCCGAGAAAUGAGUGGCCUAAAUGGGAGAAUGAGCUCGCUGUCCUUUUUUGGCUAAGGUCGAUCCGGUUCGCCGGAUCCAAAUCGGGCUUCGGUCCGGUUC	RNA	IVT
6.8	CS2	**G**ACUCACUGACACAGAUCCACUCACGGACAGCGGACUGCGCGUAUGAGUGGCGGCUAAAGAGGAGAAUGAGCGCGCUGUCC*UUUUUU*GGCUAAGG	RNA	IVT
6.9	CS2_5′A	**A**ACUCACUGACACAGAUCCACUCACGGACAGCGGACUGCGCGUAUGAGUGGCGGCUAAAGAGGAGAAUGAGCGCGCUGUCC*UUUUUU*GGCUAAGG	RNA	IVT

6.10	CS2_modified primer	**G**ACUCACUGACACAGAUCCACUCACGGACAGCGGACUGCGCGUAUGAGUGGCGGCUAAAGAGGAGAAUGAGCGCGCUGUCC*UUUUUU*CCGAUUCC	RNA	IVT
6.11	CS2_SHAPE	GGCCUUCGGGCCAA**G**ACUCACUGACACAGAUCCACUCACGGACAGCGGACUGCGCGUAUGAGUGGCGGCUAAAGAGGAGAAUGAGCGCGCUGUCC*UUUUUU*GGCUAAGGUCGAUCCGGUUCGCCGGAUCCAAAUCGGGCUUCGGUCCGGUUC	RNA	IVT
6.12	CS3	GACUCACUGACACAGAUCCACUCACGGACAGCGACGGGUGGGUAAUCUAGUGUCCGCGGAAUAGAACGAAACACGCUGUCC*UUUUUU*GGCUAAGG	RNA	IVT
6.13	CS4	**G**ACUCACUGACACAGAUCCACUCACGGACAGCGGGAUGGUGCGAACUGAGUGGGCUAAUUAGGAGAAUGAGCGCGCUGUCC*UUUUUU*GGCUAAGG	RNA	IVT
6.14	CS4_5′A	**A**ACUCACUGACACAGAUCCACUCACGGACAGCGGGAUGGUGCGAACUGAGUGGGCUAAUUAGGAGAAUGAGCGCGCUGUCC*UUUUUU*GGCUAAGG	RNA	IVT
6.15	CS4_modified primer	**G**ACUCACUGACACAGAUCCACUCACGGACAGCGGGAUGGUGCGAACUGAGUGGGCUAAUUAGGAGAAUGAGCGCGCUGUCC*UUUUUU*CCGAUUCC	RNA	IVT
6.16	CS4_SHAPE	GGCCUUCGGGCCAA**G**ACUCACUGACACAGAUCCACUCACGGACAGCGGGAUGGUGCGAACUGAGUGGGCUAAUUAGGAGAAUGAGCGCGCUGUCC*UUUUUU*GGCUAAGGUCGAUCCGGUUCGCCGGAUCCAAAUCGGGCUUCGGUCCGGUUC	RNA	IVT
6.17	CS5	**G**ACUCACUGACACAGAUCCACUCACGGACAGCGGGAGGGUGACAUCGUUGAGAGAGAAUGGGGAUAUUGAACUCGCUGUCC*UUUUUU*GGCUAAGG	RNA	IVT
6.18	CS5_5′A	**A**ACUCACUGACACAGAUCCACUCACGGACAGCGGGAGGGUGACAUCGUUGAGAGAGAAUGGGGAUAUUGAACUCGCUGUCC*UUUUUU*GGCUAAGG	RNA	IVT
6.19	CS5_modified primer	**G**ACUCACUGACACAGAUCCACUCACGGACAGCGGGAGGGUGACAUCGUUGAGAGAGAAUGGGGAUAUUGAACUCGCUGUCC*UUUUUU*CCGAUUCC	RNA	IVT
6.20	CS5_SHAPE	GGCCUUCGGGCCAA**G**ACUCACUGACACAGAUCCACUCACGGACAGCGGGAGGGUGACAUCGUUGAGAGAGAAUGGGGAUAUUGAACUCGCUGUCC*UUUUUU*GGCUAAGGUCGAUCCGGUUCGCCGGAUCCAAAUCGGGCUUCGGUCCGGUUC	RNA	IVT
7.1	PPP-CS1_pc1	(5′-triphosphate)-GACUCACUGACACAGAUCCACUCAC	RNA	IVT
7.2	P-CS1_pc1	(5′-monophosphate)-GACUCACUGACACAGAUCCACUCAC	RNA	IDT
7.3	HO-CS1_pc1	(5′-hydroxyl)-GACUCACUGACACAGAUCCACUCAC	RNA	IDT
7.4	P-CS1_pc2	(5′-monophosphate)-GGACAGCGGACAGCCGAGAAAUGAGUGGCCUAAAUGGGAG	RNA	IDT
7.5	P-CS1_pc3	(5′-monophosphate)-AAUGAGCUCGCUGUCCUUUUUUGGCUAAGG	RNA	IDT
7.6	CS1_splint1	CTCGGCTGTCCGCTGTCCGTGAGTGGATCTGTGTCAG	DNA	IDT
7.7	CS1_splint2	CCAAAAAAGGACAGCGAGCTCATTCTCCCATTTAGGCCAC	DNA	IDT
8.1	FAM-HP_sub	(5′-FAM)-ACCACCGCAUUCCGC**AG**UCCUCUCC	RNA	IDT

8.2	HP_ribozyme	GGAGAGAGAAGCGGACCAGAGAAACACACGUU GUGGUAUAUUACCUGGUA	RNA	IVT
