# Supplementary material for: A ribozyme ligase that requires a 3′ terminal phosphate on its RNA substrate
Source: Nat Commun. 2026 Jul 13;17:5634. doi: 10.1038/s41467-026-74622-8 (PMC13365474; doi:10.1038/s41467-026-74622-8)
Supplement: Supplementary file 5 — Transparent Peer Review file [file 41467_2026_74622_MOESM5_ESM.pdf]

nature portfolio

Peer Review File

A ribozyme ligase that requires a 3′ terminal phosphate on its RNA substrate

Corresponding Author: Dr Saurja DasGupta

This file contains all reviewer reports in order by version, followed by all author rebuttals in order by version.

Attachments originally included by the reviewers as part of their assessment can be found at the end of this file.

Version 0:

Reviewer comments:

Reviewer #1

(Remarks to the Author)
Summary

This study is based on a previous in vitro selection study that isolated ligase ribozymes. The current study describes a ribozyme that catalyzes the ligation between a 2'-hydroxyl and a 5'-triphosphate - but only if the 3'-hydroxyl group vicinal to the attacking 2'-hydroxyl carries a phosphate. This dependence on a 3'-phosphate is novel. This result is interesting because it generally widens our fundamental knowledge of RNA catalysis, but more importantly because it can now be used as a tool in identifying 3'-phosphates, for example for profiling RNA cleavage products of RNAs in mixtures of cellular RNAs. This manuscript is also a reminder to not ignore 'undesired' outcomes of in vitro selection experiments because they could turn out to be very interesting.
The figures are clear, and the text is well written, except that it could sometimes focus on the big picture and less on details that are sometimes not relevant. I would like to see a more detailed discussion *why* the specific solution was found in this selection but not others. Otherwise, I believe that this manuscript is suitable for publication in Nat. Comm.

Comments

-> I think that a discussion of *why* the specific solution was found in this selection but not others would be in most reader's interest. The finding that >60% of the isolated sequences showed the dependence on the 3'-phosphate is important and should be highlighted in the abstract: This means that this is not a weird outlier but it is the dominating solution. It means that this solution has clear chemical advantages for ribozyme based ligation to carry a vicinal phosphate. Please mention in the abstract, and discuss in more detail (chapter xx) why the 3'-phosphate helps: Could it be electronic effects? Please try to find the pKAs of 3'-hydroxyl groups with 2'-OH and 2'-phosphate. In previous selections for ligase ribozymes, I am not aware that a dependence on vicinal phosphates. Please discuss whether this difference could be because the selection leading up to the peculiar behavior started from ribozymes using 5'-imidazolides as activation groups as opposed to 5'-triphosphates.

- The manuscript would be more exciting to read if the sections 'Reaction between the substrate 3'-hydroxyl group...' and 'Ligation requires...' would get to the point sooner. The exciting parts of the manuscript lie in the later findings. I suggest that these two sections are summarized into one section, and only the central experiments are described and shown in the main text and figures. The detective work could be delegated to a supplementary figure and its legend. For example, the manuscript could state 'After exploring different options for the reacting groups in the catalyzed ligations (see extended data), the results showed that the ligation occurred between the alpha-phosphate of the 5'-PPP and the 2'-hydroxyl - but only if it carried a 3'-phosphate (figure X, which focuses only on the central controls).

- I suggest that the manuscript makes more clear the possible application of this ribozyme in the title. For example, the title could be modified to 'A ribozyme ligase that detects and quantifies RNA 3'-terminal phosphates'.

- Can the results also can be explained by the nucleophilic attack of a 3'- phosphate oxygen to the alpha-phosphate of the 5'-PPP? This would form a pyrophosphate bond, and would not be thermodynamically as stablebut may be kinetically easier.

Please explicitly point out in the text if there is an assay performed in ghis study that excludes this possible explanation.

Minor comments

- In the abstract, the third-last sentence carries an incorrect implication. The sentence "... these ribozymes can [...] and selectively amplify the captured RNAs" implies that these ribozymes are amplifying RNAs, which would be incorrect. Please clarify the sentence.

- the third sentence of the introduction requires one or more references.

- in figure 1, please give an estimate of the complexity of the initial library for the selection.

- At the end of page 5, please state what fraction of the clusters appear to fold into the same, or a different secondary structure as the parent ribozyme. While this could be helped by secondary structure prediction, it would be best to base it on the preservation or non-preservation of positions hat are conserved in the wildtype.

- figure 3C: For consistency, please provide the same column graph for fig. 3C that 3B provi\des for 3A. I suggest combining 3A and 3B into one sub-panel, then combining the current figure 3C with its graph into a second sub-panel. Similarly, I suggest doing the same for 3D and 3E.

- page 8, sentence 3: Please remove 'strongly'.

- page 8, end of the first paragraph: I don't understand why the slope of 1 is a feature of hydroxyl groups (as opposed to mercapto groups, for example). Please clarify or remove.

Reviewer #2

(Remarks to the Author)
This manuscript by Biswas, Dasgupta and colleagues describes the discovery of a new class of ligase ribozymes that catalyzes the attack of the 2'-OH of an RNA substrate on its own 5'-triphosphate group but only when the substrate RNA is phosphorylated on the 3'-OH vicinal to its 2'-OH. This unusual ribozyme reactivity, serendipitously isolated from a previous selection experiment aiming at another ligation activity, is proposed to be reminiscent of those of protein-based RNA repair. The isolated ribozymes are also proposed to have some potential utilities as enrichment reagents for profiling RNA cleavage products in transcriptomic studies.
The proposed chemical reactivity of this new class of interesting ribozymes is well supported by the experimental data and is certainly worth publishing somewhere. However, as listed below, more work needs to be performed with respect of the structural 2D characterization of this class of ribozymes. For instance, it is not clear how the RNA substrates are recognized by this class of ribozymes. While it would be certainly interesting to have ribozymes for fishing any 3' phosphorylated RNAs in a mixture of RNAs, the data presented herein are far from convincingly showing that this could be achieve with the ribozymes presented in this study, even by using them as possible lead compounds for further selection experiments. For instance, the RNA substrate sequence dependency of these ribozymes is still far too strong for suggesting that these ribozymes could be general enough for any practical application. Moreover, based on the data provided, the proposal that ribozyme-catalyzed ligation of 3'-phosphorylated RNA could have enabled the repair of cleaved RNA strands in primordial cell remain far too speculative.

Overall, this manuscript presents a very interesting new ribozyme activity but more work needs to be done, especially at a structural level, for suggesting a potential biotechnological use and prebiotic roles of this type of ribozymes.

Major comments

(1) The ribozymes described herein are not general enough for fishing any 3' phosphorylated RNAs. This really limits the scope of the article with respect of possible applications for fishing out 3'P RNA substrates in transcriptomic studies. Moreover, this also strongly limit the prebiotic potential of these ribozymes in mechanism of RNA repair.

(2) Part of the issue for addressing the potential general use of these ribozymes is the lack of information about the RNA substrate binding site. Therefore, it would be of great value to this article to provide a clear identification of the substrate binding site at the level of these ribozymes. Several additional analysis and experiments could be performed to address this question. By addressing them, the scope of the article is likely to increase.

(2.a) First, it is surprising that the authors did not mention anything about the similarity in sequence logos for the CS1, CS2, CS4 ribozymes, and also to a lesser extend CS5 ribozymes. As a matter of fact, these various ribozymes are likely to fold into a similar 2D structure suggesting that they belong to the same structural class of ribozymes. It might therefore be possible for the authors to determine the 2D structure of these ribozymes by sequence comparative analysis. It is possible that the structure of these ribozymes might include pseudoknots...
(2.b) Second, the authors have stated that, “since the CS1 ribozyme does not require the external template, we were surprised to find that CS2, CS4, and CS5 did require an external template for activity (Extended Data Fig. 9A)." As a matter of fact, CS1 is still working better with the external template than without. This strongly suggests that the external template has a beneficial role for this structural class of ribozyme that should also be uncovered.

(2.c) Third, the authors provide some SHAPE analysis of CS1. However, the experiment performed is unlikely to be conclusive to anything. It would be necessary to perform the same type of experiments in presence and absence of substrate, with or without external template. Moreover, the authors could also perform the experiment with 3'-phosphorylated and/or unphosphorylated substrates. In addition to CS1, the analysis could also be performed on CS2 or CS4 so that similarities or differences could be identified. This would be more informative for eventually identifying the 2D structure of this class of ribozymes as well as the substrate binding site. The 2D of the active form of CS1 is very unlikely to be like the one proposed by the authors in Fig S15. Indeed, CS1 might fold into its active structure only when it is in presence of substrate. In the case of CS2, the active structure might only be reached in presence of external template in addition to the substrate.

Minor comments

On most figures and figure legends (e.g Fig 3 and Fig 4), the reaction times are not indicated.

Reviewer #3

(Remarks to the Author)
The authors report the serendipitous discovery and thorough characterization of a new ribozyme activity that may be related to replication/ligation/error correction activity in a prebiotic RNA world. Interestingly, this activity was not the intended one that the in vitro selection was carried out for, and this will become a popular example for SELEX evolving unexpected activities.
The work is significant and state-of-the-art. All necessary controls have been carried out, and citations are appropriated. The authors carefully interpret their data. The level of detail is sufficient.
I have only one minor suggestion. It is, in my opinion, surprising that the charge of the 3'-phosphate does not seem to matter. In the oligo used in the selection, this was a phosphodiester, but in the optimized format, a phosphomonoester works as well. As this phosphate is very close to the reaction center, one could try to probe this effect more directly.
Nice work.

Version 1:

Reviewer comments:

Reviewer #1

(Remarks to the Author)
The revised manuscript has satisfactorily addressed all ofmy comments. I support publication of the revised manuscript.

Reviewer #2

(Remarks to the Author)
This revised version of the manuscript by Biswas, Dasgupta and colleagues describes the discovery of a new class of ligase ribozymes that catalyzes the attack of the 2'-OH of an RNA substrate on its own 5'-triphosphate group but only when the substrate RNA is phosphorylated on the 3'-OH vicinal to its 2'-OH.
The proposed chemical reactivity of this new class of interesting ribozymes is presently well supported by all the experimental data provided. Moreover, the authors have also provided additional pieces of information in order to address some comments pertaining to the structures of these ribozymes.
This article is certainly worth publishing but, in light of the new data provided, the discussion and conclusions pertaining to the structural characterization of these ribozymes need to be revised. As shown in the comments below and attached figure, the sequence comparative analysis of the four sequence families (CS1, CS2, CS4 and CS5) can readily provide more meaningful information on the structure of these ribozymes, strongly suggesting that CS1, CS2, CS4 and, to a lesser extend CS5, fold into a common catalytic core likely presenting pseudo-knots. Of course, as the full list of all the RNA sequences obtained for each cluster was not provided, a more extensive analysis of these sequences could certainly validate further some of the pairings uncovered below.

The authors have not performed the SHAPE analysis in presence of template and substrate. Therefore, it is likely that some of the ribozymes that they characterized by SHAPE might be in a misfolded state. This said, it is still possible to see some interesting chemical probing trends that could corroborate that CS1, CS2, CS4 and CS5 fold into the same structure rather than different ones as proposed by the authors. Clearly, using mfold as the method for folding these 2D structures is not ideal for predicting pseudoknots and should not be used if alternative, more powerful approaches are at hand such as sequence comparative analysis. Overall, the 2D structures shown in Extended figure 12 show several regions that seem to be protected while they are located in terminal or internal loops. Moreover, they also show some regions that are paired that are not protected. This indicates that the proposed mfold structures are unlikely to be correct and as seen below, might include pseudo-knot pairings...

Comments

(A) First, the extended Figure 11 should be revised in order to include indel mutations. Using CS1 as the reference and considering single point mutations and indel mutations, the Levensthein distance of CS1 from RS1 is 13 mutations, while

CS2 and CS4 are at 10 and 11 mutations away from CS1 which is significantly different from what is seen presently shown in extended Figure 11.

(B) Second, by taking as a hypothesis that CS1, CS2, CS4 and CS5 could fold into the same core, it is possible to provide by sequence comparative analysis a lot of information supporting this claim. Moreover, the SHAPE data could also make more sense, albeit it was performed without template and substrate.

As the authors did not try to investigate the structures of their ribozymes by sequence comparative analysis, to give them some hints, they will find attached a new alignment for CS1, CS2, CS4 and CS5 taking into consideration indels (attached Figure file).
As seen in this alignment, CS1, CS2 and CS4 share a similar sequence signature, with 27 nucleotides in common (see below). For instance, positions 14-20 of box 1 (UGAGUGG), 22-25 of box 2 (CUAA) and 29-39 of box 3 (GGAGAAUGAGC) are conserved between CS1, CS2 and CS4 (shaded nts) while the first and third signature boxes are 3 and 2 mutations away from RS1. This suggests that CS1, CS2 and CS4 might share a common core, distinct from RS1. As a matter of fact, CS1, CS2, CS4, and to a lesser extent CS5, share a similar core that could all include stems P1 and P2, forming a pseudoknot.
Indeed, box 1 is complementary to the sequence CCACUCA localized in the 5' constant region of these constructs, suggesting that CS1, CS2, CS4 and to a lesser extent CS5, might share the conserved stem P1. This stem is predicted by mfold for CS1, CS2 and CS4 as indicated in the proposed secondary structures in Extended Fig 12. However, it can also be formed in CS5 but is it only involving 4 or 5 bps. Note that this stem is validated by SHAPE probing for all the CS1, CS2, CS4 and CS5 constructs. For instance, in CS5, the sequence UGAG of box 1 is protected in shape indicating that the mfold is wrong and that a 2D more closely matching the one of CS1, CS2 and CS4 is strongly warranted. In extended Figure 12E, the region (21-24) CUCA is likely paired to region (49-52) UGAG forming a pseudoknot interaction. This means that the 2D structures of CS1, CS2, CS4 and CS5 could all be drawn in a way to emphasize the similarities between them. For instance, stem P1 can co-exist with stem P2 in all CS constructs. Indeed, stem P2, which is the constant paired region formed between nts(26-33) and nts(74-81) in RS1, could still be formed in CS1, CS2, CS4 and CS5 ribozymes. However, there is a lack of information as far as it concerns the number of bps involved in P2 and it is possible that its length might slightly vary from on CS construct to another as there is also an additional tertiary pairing that is validated by a covariation (see next section).

A closer look at the clustered sequences from extended data Fig 1 could provide strong “phylogenetic” clues for proposing 2D diagrams that might illustrate better the closeness of the four ribozymes clusters.
As an example, by looking at extended Fig 1A, the sequence data of CS1 likely corroborates a covariation between position 9 (A42 in CS1 extended data Fig 12) and position 40 (U73 in CS1 extended data Fig 12). It looks like that some CS1 sequences have a C at position 9 when it is a G at position 40. Moreover, when position G10 changes into C, position C39 changes into G... This would validate the existence of (at least) the WC bp doublet between A9G10 and C39U40. Interestingly, in CS2 and CS4, the equivalent positions are C8G9/C39G40 and C9G10/C39G40, respectively. In the cluster of CS4 in Extended Fig 1D, it seems that C9G10/C39G40 could also be changed into A9G10/C39U40 and/or C9U10/A39G40. The presence of this tertiary bp doublet validated by sequence comparative analysis in CS1, CS2 and CS4 strongly suggests that they fold into similar secondary and tertiary structures for their catalytic core.

The fact that the template is still useful for optimal activity of the CS ribozymes would suggest that their 3’ region is still complementary to the template and not used to form base pairs with the rest of the RNA sequence. This would disqualify any of the proposed 3 to 4 bps stems involving the template binding site. As a matter of fact, any proposed internal stems shown in the 2D structures from extended Figure 12 that involve the template binding site are either not there or due to misfolding... Note that the SHAPE reactivity observed in CS1 at the 3’ end template binding region could also be due to misfolding as the template was not present... Likewise in CS2, the surrounding of box 2 might look more protected than in CS1, CS4 and CS5 but this could be artifactual as it might result from an alternative fold resulting from the absence of the template (and substrate). In all cases, the SHAPE reactivity in the 3’ end region of all CS constructs does not mean much because of the template is lacking in SHAPE experiments.

Another clue suggesting that they have similar fold is that the box 3 region is highly modified by SHAPE in all CS constructs. Moreover, box 2 is similarly accessible in CS1, CS4 and CS5. Box 2 and 3 are highly conserved in CS1, CS2 and CS4 and this region is quite intriguing. It might adopt an unusual RNA fold (a G-quartet???), or could be involved in the recognition of the substrate and/or involved in the formation of the catalytic site.

In any case, a similar catalytic core for CS1, CS2, CS4 and CS5 is supported by the facts that:
(1) CS1, CS2, CS4 and CS5 have the same activity,
(2) CS1, CS2, CS4 and CS5 seem to have the same type of sequence reliance with the template, albeit the template is dispensable for the best class of ribozymes (CS1)
(3) Comparative sequence analysis warrants the presence of similar pseudo-knots with similar conserved regions in all CS constructs and demonstrate the presence of a covariation between position x and y for all CS constructs.
(4) The new SHAPE data is best explained by assuming that the CS constructs adopt a similar structure.

In conclusion, the authors should highlight these findings relative to the 2D/3D structure of the CS ribozymes and modify their article accordingly.
Finally, as the authors mentioned that they have a preliminary X-ray structure of the CS1 ribozyme, nothing should prevent them to take advantage of some of the preliminary data for showing that the CS2, CS4 and eventually CS5 families are likely to fold into the same active 2D/3D structures as the one of CS1 unless their CS1 ribozyme atomic structure does not

correspond to the native active fold...

Version 2:

Reviewer comments:

Reviewer #2

(Remarks to the Author)
The authors have addressed my comments and I support publication of the revised manuscript.

Open Access This Peer Review File is licensed under a Creative Commons Attribution 4.0 International License, which permits use, sharing, adaptation, distribution and reproduction in any medium or format, as long as you give appropriate credit to the original author(s) and the source, provide a link to the Creative Commons license, and indicate if changes were made.
In cases where reviewers are anonymous, credit should be given to 'Anonymous Referee' and the source.
The images or other third party material in this Peer Review File are included in the article's Creative Commons license, unless indicated otherwise in a credit line to the material. If material is not included in the article's Creative Commons license and your intended use is not permitted by statutory regulation or exceeds the permitted use, you will need to obtain permission directly from the copyright holder.
To view a copy of this license, visit https://creativecommons.org/licenses/by/4.0/

RESPONSE TO REVIEWERS' COMMENTS

We thank the Editor and Reviewers for their critical reading of our manuscript and insightful comments that have improved our manuscript. We have responded to each comment below in blue. Changes to the text are indicated in red in the revised manuscript.

Reviewer #1 (Remarks to the Author):

Summary

This study is based on a previous in vitro selection study that isolated ligase ribozymes. The current study describes a ribozyme that catalyzes the ligation between a 2'-hydroxyl and a 5'-triphosphate - but only if the 3'-hydroxyl group vicinal to the attacking 2'-hydroxyl carries a phosphate. This dependence on a 3'-phosphate is novel. This result is interesting because it generally widens our fundamental knowledge of RNA catalysis, but more importantly because it can now be used as a tool in identifying 3'-phosphates, for example for profiling RNA cleavage products of RNAs in mixtures of cellular RNAs. This manuscript is also a reminder to not ignore 'undesired' outcomes of in vitro selection experiments because they could turn out to be very interesting. The figures are clear, and the text is well written, except that it could sometimes focus on the big picture and less on details that are sometimes not relevant. I would like to see a more detailed discussion *why* the specific solution was found in this selection but not others. Otherwise, I believe that this manuscript is suitable for publication in Nat. Comm.

We thank the reviewer for recommending our work for publication in *Nature Communications*.
A point-wise response to each comment is provided below:

Comments

-> I think that a discussion of *why* the specific solution was found in this selection but not others would be in most reader's interest. The finding that >60% of the isolated sequences showed the dependence on the 3'-phosphate is important and should be highlighted in the abstract: This means that this is not a weird outlier but it is the dominating solution. It means that this solution has clear chemical advantages for ribozyme based ligation to carry a vicinal phosphate. Please mention in the abstract, and discuss in more detail (chapter xx) why the 3'-phosphate helps: Could it be electronic effects? Please try to find the pKAs of 3'-hydroxyl groups with 2'-OH and 2'-phosphate. In previous selections for ligase ribozymes, I am not aware that a dependence on vicinal phosphates. Please discuss whether this difference could be because the selection leading up to the peculiar behavior started from ribozymes using 5'-imidazolides as activation groups as opposed to 5'-triphosphates.

The reason for the ribozymes' specificity for a 3′-phosphate group on the substrate is an intriguing question. We surveyed the literature as suggested by the reviewer and found that the presence of a 3′-phosphate vicinal to a 2′-hydroxyl renders the latter less nucleophilic (Jash and Kool, *Chem Commun.*, 2022), not more. This result is consistent with an older study with mononucleotides, which measured the pKa of 2′-hydroxyl groups in the presence or absence of 3′-phosphates, and found that the pKa of a 2′-hydroxyl of adenosine is ~12.4, which rises by 1 unit to ~13.4 in adenosine 3′-phosphates (Velikyan et al. *JACS*, 2001). These results could stem from the electrostatic repulsion between the deprotonated 3′-O^- and the non-bridging Os of the 3′-phosphate. Therefore, the 3′-phosphate does not, by itself, assist in this reaction, and instead the ribozymes must overcome this slight reduction in reactivity by utilizing the 3′-phosphate in a catalytic role. In fact, now we show that a 2′-phosphorylated substrate with a 3′-hydroxyl group is not reactive for ligation (Fig. 4C), indicating that the ribozyme is unable to activate a 3′-OH in the presence of a vicinal 2′-P, further confirming catalytic specificity. The strict requirement for Mg^{2+} as the divalent cation and a modest but reproducible metal rescue of 3′-thiophosphate substrates with thiophilic Cd^{2+} (Extended Fig. 8), point to localization of a catalytic metal ion in the ligation junction as the most plausible role of 3′-phosphate. This is currently under structural

investigation.

We have now added the following text to the revised manuscript to elaborate on this aspect:
"Although a 3′-P can, in principle, accelerate substrate ligation by activating the vicinal 2′-OH nucleophile by acting as a general base catalyst, similar ligation rates with Substrate-3′P, which contains a phosphomonoester with a pKa ~7 and Substrate-TEG-Biot (Fig. 4D), which contains a phosphodiester with a pKa ~2, discount this potential catalytic role. In fact, the presence of 3′-phosphate or 3′-phosphodiester groups lowers the nucleophilicity of the vicinal 2′-hydroxyl group, [18] which is supported by slight increase in the 2′-OH pKa in the presence of a 3′-phosphate relative to a 3′-hydroxyl (~13.4 vs. ~12. 4).[19]"

We have not added the finding that >60% of the isolated sequences showed the dependence on the 3'-phosphate to the abstract, although it is an interesting result, due to the 200-word limit of the abstract in this journal.

The unique specificity to substrate 3′-phosphate could not have been a feature of the parental AIP-ligase as this ribozyme catalyzes ligation between its 3′ end and the 5′ end of the substrate. However, CS1, the dominant sequence with this new reactivity, emerged early on during selection from a partially-mutagenized library derived from the parental AIP-ligase. We have reported a detailed analysis of how sequences in the CS1-CS5 clusters were enriched across six rounds of selection elsewhere (DasGupta et al., *Proc. Natl. Acad. Sci.*, 2024).

We would also like to point the reviewer to the following text, where we discuss why ribozymes with this unexpected reactivity may have survived our selection protocol that was designed to isolate ribozymes that catalyze ligation between their 3′ ends and the 5′ end of the substrate:
"As the isolated ribozymes catalyze substrate ligation to their 5′ ends as opposed to their 3′ ends, we were surprised that these sequences could be reverse transcribed at 42 ºC using a primer (RT primer) that binds to its 3′ end by forming just 4 base-pairs. This indicates that the isolated sequences survived this step in the selection cycle by exploiting weak interactions between the RT primer and their 3′ ends (Extended Data Fig. 9)."

- The manuscript would be more exciting to read if the sections 'Reaction between the substrate 3'-hydroxyl group...' and 'Ligation requires...' would get to the point sooner. The exciting parts of the manuscript lie in the later findings. I suggest that these two sections are summarized into one section, and only the central experiments are described and shown in the main text and figures. The detective work could be delegated to a supplementary figure and its legend. For example, the manuscript could state 'After exploring different options for the reacting groups in the catalyzed ligations (see extended data), the results showed that the ligation occurred between the alpha-phosphate of the 5'-PPP and the 2'-hydroxyl - but only if it carried a 3'-phosphate (figure X, which focuses only on the central controls).

We appreciate the reviewer's suggestion to streamline our manuscript. The revised manuscript has been rewritten to reduce redundancy, and we have consolidated results together, wherever possible. We have now merged the two sections: 'Unexpected ligation to the ribozyme 5′ end' and 'Reaction between the substrate 2′-hydroxyl and the ribozyme 5′-triphosphate groups' into a single section 'Unexpected reaction between the substrate 2′-hydroxyl and the ribozyme 5′-triphosphate groups'. We have kept 'Ligation requires a 3′-phosphate group on the substrate' as standalone section because this section discusses key results about the novel reactivity of the reported ribozymes.

- I suggest that the manuscript makes more clear the possible application of this ribozyme in the title. For example, the title could be modified to 'A ribozyme ligase that detects and quantifies RNA 3'-terminal phosphates'.

While we are excited about the application of the reported ribozymes as specific reagents for probing 3′-phosphorylated RNA cleavage fragments, we want to be cautious about such claims at this time before we validate this tool in proof-of-concept transcriptomics studies. The purpose of this manuscript

is to report the discovery of a new ribozyme reactivity and present a potential application of this unique reactivity.

- Can the results also can be explained by the nucleophilic attack of a 3'- phosphate oxygen to the alpha-phosphate of the 5'-PPP? This would form a pyrophosphate bond, and would not be thermodynamically as stable but may be kinetically easier. Please explicitly point out in the text if there is an assay performed in this study that excludes this possible explanation.

This is an interesting suggestion. In the original manuscript, we showed that CS1 is inactive for ligation when the 2′-OH of the terminal nucleotide (rA16) of the 3′-biotinylated substrate is changed to a deoxynucleotide (Fig. 3F in the revised manuscript). The 3′ end of this substrate has a phosphodiester moiety, not a monophosphate; therefore, in the revised manuscript, we have performed an additional assay with substrates containing 3′-phosphate (phosphomonoester) groups, which demonstrates that substrates with a terminal deoxynucleotide (dA16) are not ligated by CS1 (Fig. R1A).
This figure has been added to the revised manuscript (Extended Data Fig. 5A) and the text has been modified accordingly to:
"3′-biotinylated or 3′-phosphorylated substrates where the terminal adenine ribonucleotides (rA16) are replaced by deoxyribonucleotides (dA16) were inactive for ligation, confirming the substrate 2′-OH as the nucleophile (Fig. 3F and Extended Data Fig. 5A)."

Additionally, we tested CS1 for ligation with a 2′-phosphorylated substrate that contains a 3′-hydroxyl. Absence of ligation confirmed that CS1 cannot activate a 3′-OH in the presence of a vicinal 2′-P or use a 2′-P as a nucleophile (Fig. R1B). This figure has been added to the revised manuscript (Fig. 4C) and the following text has been added:
"The specific requirement for the substrate terminal configuration, i.e., 2′-OH, 3′-P was further highlighted by the lack of reactivity of a substrate with a 3′-OH, 2′-P terminus (Substrate16dA-2′P). (Fig. 4C)."

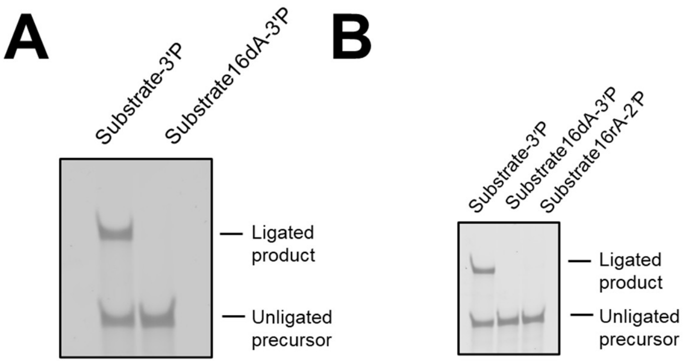

Figure R1. The reactivity of ribozymes toward substrates with different 3′ termini. **A.** CS1-catalyzed ligation with 3′-phosphorylated substrates involves the substrate's terminal 2′ hydroxyl group. **B.** CS1 cannot use the substrate terminal 2′ phosphate or the terminal 3′ hydroxyl group for ligation.

Minor Comments

- In the abstract, the third-last sentence carries an incorrect implication. The sentence "... these ribozymes can [...] and selectively amplify the captured RNAs" implies that these ribozymes are amplifying RNAs, which would be incorrect. Please clarify the sentence.

This sentence has been modified to "We show that these ribozymes can capture cleaved RNAs with 3′-phosphate and 2′-3′-cyclic phosphate termini enabling us to selectively amplify the captured RNAs" to clarify that the ribozymes can capture cleaved RNAs but do not amplify them.

- the third sentence of the introduction requires one or more references.

We have added the following reference to the revised manuscript:
Gilbert, W., The RNA world, *Nature*, **1986**, 319, 618

- in figure 1, please give an estimate of the complexity of the initial library for the selection.

The estimated complexity of the initial library for the selection was~10^{14} sequences. We have added this information in the Fig. 1 legend.

- At the end of page 5, please state what fraction of the clusters appear to fold into the same, or a different secondary structure as the parent ribozyme. While this could be helped by secondary structure prediction, it would be best to base it on the preservation or non-preservation of positions that are conserved in the wildtype.

We thank the reviewer for this suggestion. The secondary structures of CS2, CS4, and CS5 as predicted by the mFOLD server (https://www.unafold.org/) are shown below (Fig. R2) and suggest distinct structures that diverge from the experimentally-determined secondary structure of the parent AIP-ligase, RS1 (Walton, T. et al., *Proc. Natl. Acad. Sci.* 2020; DasGupta, S. et al., *Proc. Natl. Acad. Sci.* 2024).

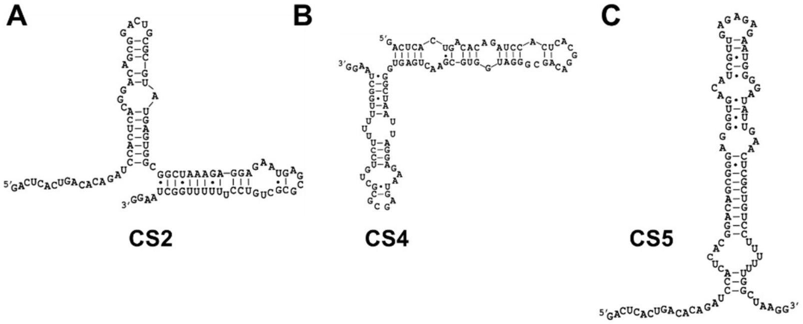

Figure R2. Computationally-predicted secondary structure of the ribozymes **A.** CS2, **B.** CS4, and **C.** CS5 by the mFOLD algorithm.

Although, we should note that another secondary structure prediction software, RNAstructure predicts a common lowest energy structure for CS1, CS2, and CS4 (but not for CS5), which is shown below (Fig R3): (https://rna.urmc.rochester.edu/RNAstructureWeb/Servers/TurboFold/TurboFold.html):

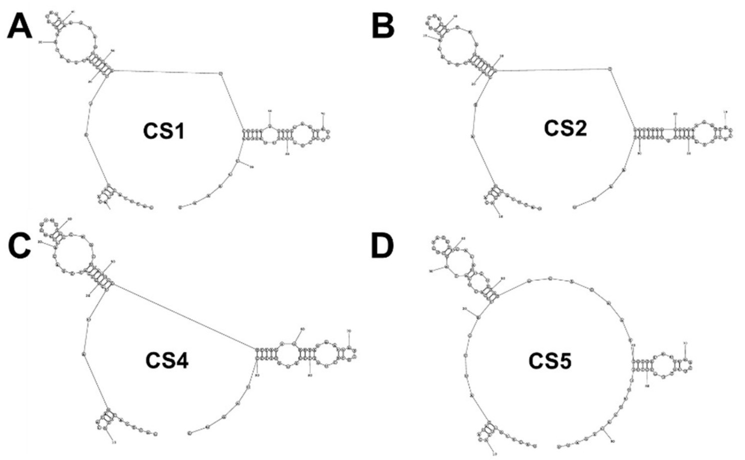

Figure R3. Computationally-predicted 'lowest-energy common' secondary structures of the ribozymes **A.** CS1, **B.** CS2, **C.** CS4, and **D.** CS5 by the RNAstructure algorithm.

However, given the limited reliability and accuracy of secondary structure prediction software, we have now provided experimentally-determined secondary structures of CS2, CS4, and CS5 (Fig. R4 C-E below) in the revised manuscript, in addition to CS1 (Extended Data Fig. 12B), which we had included previously. These SHAPE-derived structures (Extended Data Fig. 12 B-E) are also distinct from the parent AIP-ligase, RS1 (Panel A below, Extended Data Fig. 12A). The structural divergence is consistent with their sequence divergence: CS1, CS2, CS4, and CS5 are 10-16 mutations from the parental RS1, and 11-22 mutations from each other (Table 1, Extended Data Fig. 11).

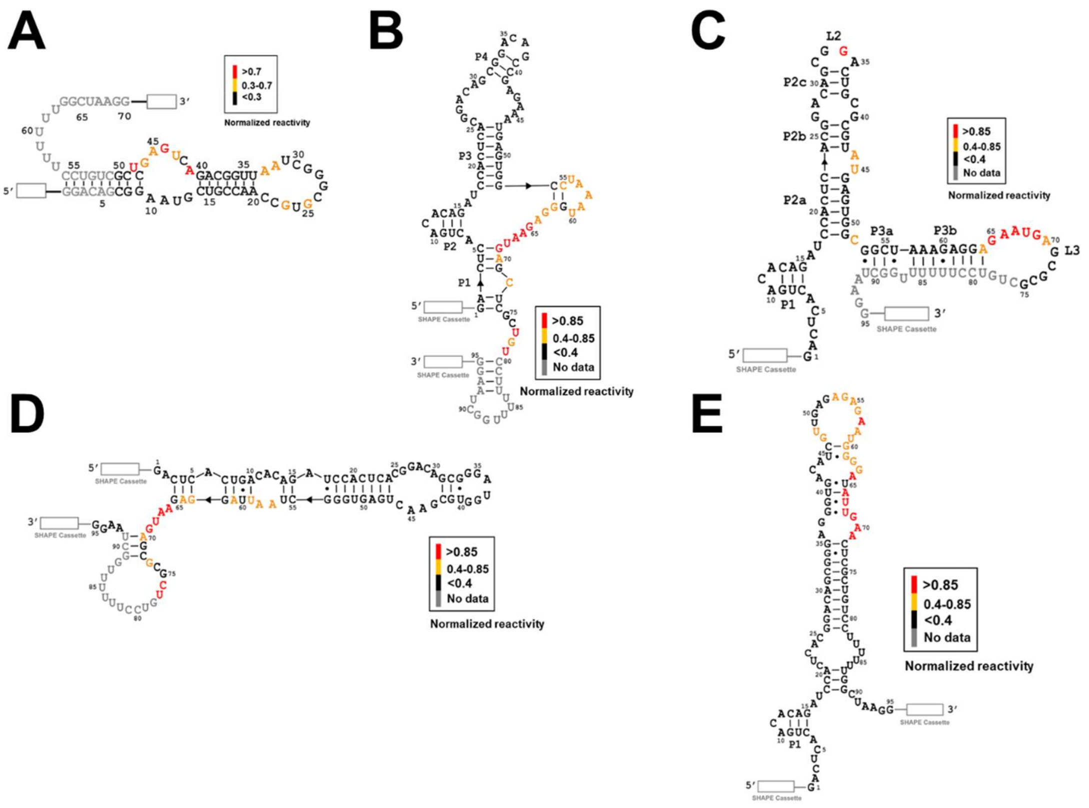

Figure R4. Secondary structure of the ribozymes A. RS1, **B.** CS1, **C.** CS2, **D.** CS4, and **E.** CS5 determined by the RNAstructure program using reactivity constraints obtained from SHAPE experiments. 5′ and 3′ SHAPE cassettes are denoted by white rectangles. Nucleotides for which no data were obtained are shown in gray. Nucleotides are colored in red, orange, and black according to their normalized SHAPE reactivities, as shown in the reactivity legend.

- figure 3C: For consistency, please provide the same column graph for fig. 3C that 3B provi\des for 3A. I suggest combining 3A and 3B into one sub-panel, then combining the current figure 3C with its graph into a second sub-panel. Similarly, I suggest doing the same for 3D and 3E.

As per the reviewer's suggestion, we have added this as a new figure panel (Fig. 3D, Fig R5 below) in the revised manuscript. Although the 14-nucleotide truncation at the 3′ end of the ribozyme preserves ligation, it slows down ligation by 8-fold relative to the full-length CS1.

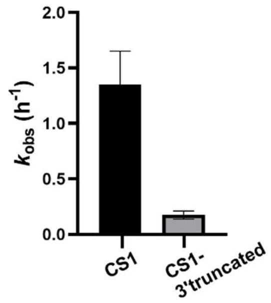

Figure R5. 3′truncation of CS1 reduces its ligation rate by 8-fold. Deleting 14 nucleotides from the 3′ end of CS1 resulted in 8-fold slower ligation relative to full-length CS1.

- page 8, sentence 3: Please remove 'strongly'.

We have removed the word 'strongly'.

- page 8, end of the first paragraph: I don't understand why the slope of 1 is a feature of hydroxyl groups (as opposed to mercapto groups, for example). Please clarify or remove.

We have now changed the sentence to "This usually indicates a single H^+ transfer in the rate-determining step in this pH regime involving the nucleophilic hydroxyl group on the substrate.". Please see: Bergman, Johnston, and Bartel, *Biochemistry* 2000, 39, 3115-3123 (Ref 16 in the main text) for reference.

Reviewer #2 (Remarks to the Author):

This manuscript by Biswas, Dasgupta and colleagues describes the discovery of a new class of ligase ribozymes that catalyzes the attack of the 2'-OH of an RNA substrate on its own 5'-triphosphate group but only when the substrate RNA is phosphorylated on the 3'-OH vicinal to its 2'-OH. This unusual ribozyme reactivity, serendipitously isolated from a previous selection experiment aiming at another ligation activity, is proposed to be reminiscent of those of protein-based RNA repair. The isolated ribozymes are also proposed to have some potential utilities as enrichment reagents for profiling RNA cleavage products in transcriptomic studies.

The proposed chemical reactivity of this new class of interesting ribozymes is well supported by the experimental data and is certainly worth publishing somewhere. However, as listed below, more work needs to be performed with respect of the structural 2D characterization of this class of ribozymes. For instance, it is not clear how the RNA substrates are recognized by this class of ribozymes. While it would be certainly interesting to have ribozymes for fishing any 3' phosphorylated RNAs in a mixture

of RNAs, the data presented herein are far from convincingly showing that this could be achieve with the ribozymes presented in this study, even by using them as possible lead compounds for further selection experiments. For instance, the RNA substrate sequence dependency of these ribozymes is still far too strong for suggesting that these ribozymes could be general enough for any practical application. Moreover, based on the data provided, the proposal that ribozyme-catalyzed ligation of 3'-phosphorylated RNA could have enabled the repair of cleaved RNA strands in primordial cell remain far too speculative.

Overall, this manuscript presents a very interesting new ribozyme activity but more work needs to be done, especially at a structural level, for suggesting a potential biotechnological use and prebiotic roles of this type of ribozymes.

Major comments

(1) The ribozymes described herein are not general enough for fishing any 3' phosphorylated RNAs. This really limits the scope of the article with respect of possible applications for fishing out 3'P RNA substrates in transcriptomic studies. Moreover, this also strongly limit the prebiotic potential of these ribozymes in mechanism of RNA repair.

We agree with the reviewer that the ribozymes described in the manuscript, in their current form, may not be suitable for capturing all 3′-phosphorylated RNAs from cells owing to the dependence on the 3'-terminal adenine of the substrate (Extended Data Figs. 15 B, C and 16). However, the ribozymes reported here, with this new enzyme reactivity, are strong starting points for generating better catalytic RNA reagents for the sequence-general enrichment of cleaved RNAs with 3′-phosphorylated end. This work presents preliminary data that support this potential, and is guiding future optimization efforts.

Similarly, the model for primordial RNA repair we propose in our manuscript illustrates how ribozymes with this new reactivity may facilitate RNA repair in an RNA World without protein-based repair pathways. The specific terminal nucleotide bias of the current ribozymes is a result of the substrate sequence using in their evolution; diverse repair ribozymes in the RNA World would be able to ligate 3′-phosphorylated broken RNAs ends harboring all four residues.

(2) Part of the issue for addressing the potential general use of these ribozymes is the lack of information about the RNA substrate binding site. Therefore, it would be of great value to this article to provide a clear identification of the substrate binding site at the level of these ribozymes. Several additional analysis and experiments could be performed to address this question. By addressing them, the scope of the article is likely to increase.

We thank the reviewer for this opportunity to mention that we are actively pursuing structural investigations of the CS1 ribozyme to identify how it binds its substrate. We managed to crystallize CS1 bound to its 16-nt substrate, and we have a high-resolution structure of the ribozyme bound to its substrate. However, the structure is incomplete, and effort is ongoing to obtain better quality data.
The fact that CS1 ligates a 3′-phosphorylated 5-nt RNA (Extended Data Fig. 15A) indicates the lack of extensive base-pairing between the ribozyme and the substrate.

(2.a) First, it is surprising that the authors did not mention anything about the similarity in sequence logos for the CS1, CS2, CS4 ribozymes, and also to a lesser extend CS5 ribozymes. As a matter of fact, these various ribozymes are likely to fold into a similar 2D structure suggesting that they belong to the same structural class of ribozymes. It might therefore be possible for the authors to determine the 2D structure of these ribozymes by sequence comparative analysis. It is possible that the structure of these ribozymes might include pseudoknots...

Although CS1, CS2, CS4, and CS5 exhibit the same ligase reactivity, their sequences significantly diverge from each other. CS1, CS2, CS4 and CS5 are 10-16 mutations from the parent ligase, RS1 (Table 1, Fig R6A below) and 11-22 from each other (Extended Data Fig. 11, Fig R6B below).

A

	Sequence (40-nt variable region; 5’ → 3’)	Mutations From RS1
**RS1**	GAAUGCUGCCAACCGUGCGGGCUAAUUGGCAGACUGAGCU	-
CS1	GACAGCCGAGAAAUGAGUGGCCUAAAUGGGAGAAUGAGCU	13
CS2	GACUGCGCGUAUGAGUGGCGGCUAAAGAGGAGAAUGAGCG	16
CS4	GGAUGGUGCGAACUGAGUGGGCUAAUUAGGAGAAUGAGCG	10
CS5	GGAGGGUGACAUCGUUGAGAGAGAAUGGGGAUAUUGAACU	16

B

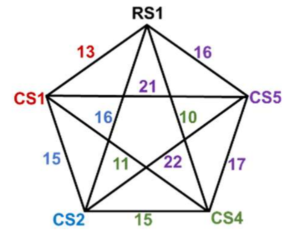

Figure R6. Sequence divergence of the evolved ribozymes from the parent ribozyme RS1. A. The 40-nt variable region from the representative sequences of the five most abundant sequence clusters, CS1, CS2, CS4, and CS5. RS1 is the parent AIP-ligase that CS1, CS2, CS4 and CS5 were evolved from. Sequence divergence from RS1 is highlighted in gray. Isolated sequences were 10-16 mutations from RS1. **B**. Sequence divergence between RS1, CS1, CS2, CS4, and CS5.

To address the reviewer’s point about 2D structure of these ribozymes, we have performed SHAPE probing on CS2, CS4, and CS5 and provided the resulting experimentally determined secondary structures in the revised manuscript, to accompany the SHAPE-derived secondary structure of CS1 that we provided in our initial submission (Extended Data Fig. 12B-E, Fig. R4 here). These results reveal that the structures of CS1, CS2, CS5, and CS5 are distinct from the parent ribozyme RS1, as well as from each other. This is consistent with the divergence of these sequences from RS1.

(2.b) Second, the authors have stated that, “since the CS1 ribozyme does not require the external template, we were surprised to find that CS2, CS4, and CS5 did require an external template for activity (Extended Data Fig. 9A)." As a matter of fact, CS1 is still working better with the external template than without. This strongly suggests that the external template has a beneficial role for this structural class of ribozyme that should also be uncovered.

CS1 is 3-fold slower without the external template as indicated by the reviewer. We have now probed this effect using a similar compensatory mutation assay as we did for CS2, CS4, and CS5 (Extended Data Fig. 10). Compensatory mutation assay with CS1 (Extended Data Fig. 10D, Fig. R7 below) suggested that the 3′ end (‘primer’) sequence of CS1, like CS2, CS4, and CS5, is involved in beneficial interactions with the last eight nucleotides of the external RNA template. Therefore, the template may interact with the first eight nucleotides of the substrate and last eight nucleotides of the ribozymes to render this three-component reaction pseudo-intramolecular thereby stimulating ligation by CS1. Unlike CS2, CS4, and CS5 however, CS1 can catalyze ligation without this template assistance.

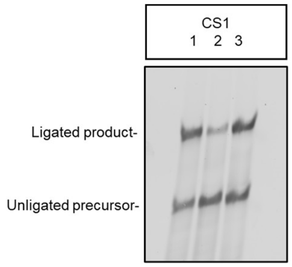

1- Primer-Template
2- Primer-Modified template
3- Modified Primer- Modified Template

Figure R7. The role of template in RNA ligation by CS1. Base-pairing interaction between the template and the 3′-primer sequence of CS1 assists ligation. Disrupting this interaction by mutations in

the template reduce ligation efficiency; however, compensatory mutations in the 3′-primer sequence of the ribozymes rescue ligation.

(2.c) Third, the authors provide some SHAPE analysis of CS1. However, the experiment performed is unlikely to be conclusive to anything. It would be necessary to perform the same type of experiments in presence and absence of substrate, with or without external template. Moreover, the authors could also perform the experiment with 3’-phosphorylated and/or unphosphorylated substrates. In addition to CS1, the analysis could also be performed on CS2 or CS4 so that similarities or differences could be identified. This would be more informative for eventually identifying the 2D structure of this class of ribozymes as well as the substrate binding site. The 2D of the active form of CS1 is very unlikely to be like the one proposed by the authors in Fig S15. Indeed, CS1 might fold into its active structure only when it is in presence of substrate. In the case of CS2, the active structure might only be reached in presence of external template in addition to the substrate.

We agree with the reviewer that getting SHAPE structures of the ribozymes with their substrates and with/without a template may provide a better description of their active fold and, maybe even, the mode of substrate binding. However, this involves performing SHAPE experiments with multiple RNA components, each of which will be modified by the SHAPE reagent, in addition to the target ribozyme. This scenario may lead to creation of alternative structures for the ribozyme, which will likely result in SHAPE profiles that are averages of their alternative structures, complicating further analysis.

We do, however, provide new SHAPE-derived structures of CS2, CS4, and CS5 (Extended Data Fig. 12C-E, Fig. R4 here), in addition to the previously provided CS1 in the revised manuscript. Moreover, as mentioned before, we are currently using X-ray crystallography to elucidate the catalytic mechanism of CS1, which includes identifying the binding interactions with its substrate.

Minor comments

On most figures and figure legends (e.g Fig 3 and Fig 4), the reaction times are not indicated.

We have incorporated the changes in the figure legends of the revised manuscript, where relevant.

Reviewer #3 (Remarks to the Author):

The authors report the serendipitous discovery and thorough characterization of a new ribozyme activity that may be related to replication/ligation/error correction activity in a prebiotic RNA world. Interestingly, this activity was not the intended one that the in vitro selection was carried out for, and this will become a popular example for SELEX evolving unexpected activities. The work is significant and state-of-the-art. All necessary controls have been carried out, and citations are appropriated. The authors carefully interpret their data. The level of detail is sufficient. I have only one minor suggestion. It is, in my opinion, surprising that the charge of the 3'-phosphate does not seem to matter. In the oligo used in the selection, this was a phosphodiester, but in the optimized format, a phosphomonoester works as well. As this phosphate is very close to the reaction center, one could try to probe this effect more directly.
Nice work.

We thank the reviewer for their enthusiasm for this work.

Because the substrates with both phosphodiesters and monoesters work equally well, we think the role of the 3′-phosphate group is to bind to a catalytic metal ion and not directly activate the 2'-OH nucleophile. We explain this argument in detail in the revised manuscript:

“Although a 3′-P can, in principle, accelerate substrate ligation by activating the vicinal 2′-OH nucleophile by acting as a general base catalyst, similar ligation rates with Substrate-3′P, which contains a phosphomonoester with a pKa ~7 and Substrate-TEG-Biot (Fig. 4C), which contains a phosphodiester

with a pKa ~2, discount this potential catalytic role. A more likely role of the 3′-P is in localizing catalytic divalent cations to the active site."

To examine the nature of the interaction between the terminal phosphate group and a putative catalytic metal ion, we tested ligation with different divalent cations (Extended Data Fig. 8C) and observed that ligation works only with Mg^{2+}. Further, we measured reaction kinetics with a substrate containing either a terminal phosphate or a thiophosphate in the presence or absence of the thiophilic cation Cd^{2+}, in the background of Mg^{2+} (Extended Data Fig. 8A, B). We observed a metal rescue value of ~2.5, which may reflect an inner-sphere Mg^{2+} interaction with the 3′-phosphate. We are trying to elucidate the catalytic mechanism of the CS1 ribozyme using X-ray crystallography, and we have an incomplete crystal structure of the ribozyme bound to the substrate, which we are trying to improve upon.

RESPONSE TO REVIEWERS' COMMENTS

REVIEWER COMMENTS

Reviewer #1 (Remarks to the Author):

The revised manuscript has satisfactorily addressed all of my comments. I support publication of the revised manuscript.

We thank the reviewer for their support.

Reviewer #2 (Remarks to the Author):

This revised version of the manuscript by Biswas, Dasgupta and colleagues describes the discovery of a new class of ligase ribozymes that catalyzes the attack of the 2'-OH of an RNA substrate on its own 5'-triphosphate group but only when the substrate RNA is phosphorylated on the 3'-OH vicinal to its 2'-OH.
The proposed chemical reactivity of this new class of interesting ribozymes is presently well supported by all the experimental data provided. Moreover, the authors have also provided additional pieces of information in order to address some comments pertaining to the structures of these ribozymes.
This article is certainly worth publishing but, in light of the new data provided, the discussion and conclusions pertaining to the structural characterization of these ribozymes need to be revised. As shown in the comments below and attached figure, the sequence comparative analysis of the four sequence families (CS1, CS2, CS4 and CS5) can readily provide more meaningful information on the structure of these ribozymes, strongly suggesting that CS1, CS2, CS4 and, to a lesser extend CS5, fold into a common catalytic core likely presenting pseudo-knots. Of course, as the full list of all the RNA sequences obtained for each cluster was not provided, a more extensive analysis of these sequences could certainly validate further some of the pairings uncovered below.

The authors have not performed the SHAPE analysis in presence of template and substrate. Therefore, it is likely that some of the ribozymes that they characterized by SHAPE might be in a misfolded state. This said, it is still possible to see some interesting chemical probing trends that could corroborate that CS1, CS2, CS4 and CS5 fold into the same structure rather than different ones as proposed by the authors. Clearly, using mfold as the method for folding these 2D structures is not ideal for predicting pseudoknots and should not be used if alternative, more powerful approaches are at hand such as sequence comparative analysis. Overall, the 2D structures shown in Extended figure 12 show several regions that seem to be protected while they are located in terminal or internal loops. Moreover, they also show some regions that are paired that are not protected. This indicates that the proposed mfold structures are unlikely to be correct and as seen below, might include pseudo-knot pairings...

We sincerely appreciate the effort the reviewer has made in their comparative sequence analysis of the ribozymes we report in this work, which has provided new structural insights. We incorporated some of the reviewer's findings in the revised version of our manuscript.

We have addressed the reviewer's comments below:

Comments

(A) First, the extended Figure 11 should be revised in order to include indel mutations. Using CS1 as the reference and considering single point mutations and indel mutations, the Levensthein distance of CS1 from RS1 is 13 mutations, while CS2 and CS4 are at 10 and 11 mutations away from CS1 which is significantly different from what is seen presently shown in extended Figure 11.

We aligned the sequences of RS1, CS1, CS2, CS4, CS5 to include indels as per the reviewer's suggestion in Extended Data Fig. 12. Although the mutational distances between some sequences decreased after alignment (e.g., CS1←→ CS2: 10 vs. 15, CS2←→ CS4: 12 vs. 15), mutational distances between other ribozyme sequences increased (e.g., CS2←→ CS5: 26 vs. 22, CS5←→ RS1: 23 vs. 16), or remain unchanged (e.g., CS1←→ RS1: 13 vs. 13, CS1←→ CS4: 11 vs. 11). While there is strong conservation in certain features in these sequences, their structural similarity is not yet firmly established; therefore, we kept Extended Data Fig. 11 to show apparent sequence divergence in these sequences (before any intentional alignment), but also added a figure with the sequences aligned (Extended Data Fig. 12).

```
RS1: GACUCACUGACACAGAUCCACUCACGGACAGCG-GAAUGCUGCCA--ACCGUGCG-GG--CUAAUUG-GCAGACUGAGCUCGCUGUCCUUUUUUGGCUAAGG

CS1: GACUCACUGACACAGAUCCACUCACGGACAGCG-GACAGCCGAGA--AAUGAGUG-GC--CUAAAUG-GGAGAAUGAGCUCGCUGUCCUUUUUUGGCUAAGG
CS2: GACUCACUGACACAGAUCCACUCACGGACAGCG-GACUGC-GCGU--A-UGAGUG-GCGGCUAAA-GAGGAGAAUGAGCGCGCUGUCCUUUUUUGGCUAAGG
CS4: GACUCACUGACACAGAUCCACUCACGGACAGCGGGAUGGU-GCGA--ACUGAGUG-GG--CUAAUU-AGGAGAAUGAGCGCGCUGUCCUUUUUUGGCUAAGG
CS5: GACUCACUGACACAGAUCCACUCACGGACAGCGGGAGGGU-GACAUCGUUGAGAGAG-----AA-UGGGGAUAUUGAACUCGCUGUCCUUUUUUGGCUAAGG
```

Fig. R1. Sequence alignment of the isolated ribozymes by considering insertions/deletions. Putative base-paired stems are underlined and colored accordingly. Nucleotides reactive to SHAPE probing are shown in bold and colored orange or red, where orange indicates a normalized SHAPE reactivity of >0.85 and red indicates a normalized SHAPE reactivity of 0.4-0.85.

(B) Second, by taking as a hypothesis that CS1, CS2, CS4 and CS5 could fold into the same core, it is possible to provide by sequence comparative analysis a lot of information supporting this claim. Moreover, the SHAPE data could also make more sense, albeit it was performed with template and substrate.
As the authors did not try to investigate the structures of their ribozymes by sequence comparative analysis, to give them some hints, they will find attached a new alignment for CS1, CS2, CS4 and CS5 taking into consideration indels (attached Figure file).

We thank the reviewer for this highly informative figure. Interestingly, we had initially prepared a similar figure, but decided to limit structural discussions in order to highlight the novel reactivity, which in our opinion, is a more interesting finding from this work. We now include a similar figure (Extended Data Fig. 12) in the revised manuscript.

As seen in this alignment, CS1, CS2 and CS4 share a similar sequence signature, with 27 nucleotides in common (see below). For instance, positions 14-20 of box 1 (UGAGUGG), 22-25 of box 2 (CUAA) and 29-39 of box 3 (GGAGAAUGAGC) are conserved between CS1, CS2 and CS4 (shaded nts) while the first and third signature boxes are 3 and 2 mutations away from RS1. This suggests that CS1, CS2 and CS4 might share a common core, distinct from RS1. As a matter of fact, CS1, CS2, CS4, and to a lesser extent CS5, share a similar core that could all include stems P1 and P2, forming a pseudoknot.
Indeed, box 1 is complementary to the sequence CCACUCA localized in the 5' constant region of these constructs, suggesting that CS1, CS2, CS4 and to a lesser extent CS5, might

share the conserved stem P1. This stem is predicted by mfold for CS1, CS2 and CS4 as indicated in the proposed secondary structures in Extended Fig 12. However, it can also be formed in CS5 but is it only involving 4 or 5 bps. Note that this stem is validated by SHAPE probing for all the CS1, CS2, CS4 and CS5 constructs. For instance, in CS5, the sequence UGAG of box 1 is protected in shape indicating that the mfold is wrong and that a 2D more closely matching the one of CS1, CS2 and CS4 is strongly warranted. In extended Figure 12E, the region (21-24) CUCA is likely paired to region (49-52) UGAG forming a pseudoknot interaction. This means that the 2D structures of CS1, CS2, CS4 and CS5 could all be drawn in a way to emphasize the similarities between them. For instance, stem P1 can co-exist with stem P2 in all CS constructs. Indeed, stem P2, which is the constant paired region formed between nts(26-33) and nts(74-81) in RS1, could still be formed in CS1, CS2, CS4 and CS5 ribozymes. However, there is a lack of information as far as it concerns the number of bps involved in P2 and it is possible that its length might slightly vary from on CS construct to another as there is also an additional tertiary pairing that is validated by a covariation (see next section).

We agree that comparative sequence analysis as the reviewer suggested generates some useful information about a possible common structure shared among these ribozymes. We have revised our manuscript to include a new section – ‘Comparative structural analysis of the isolated ligase ribozymes’ to address the points raised by the reviewer. We now mention that CS1, CS2, and CS4 likely feature a conserved stem composed of 5′-CCACUCA-3′ and 3′-GGUGAGU-5′ regions, and CS5 likely contains a shorter stem composed of 5′-CUCA-3′ and 3′-GAGU-5′. The existence of this stem (referred to P1 by the reviewer) is supported by the overall insensitivity of this region to SHAPE modification. We also mention that the longer stem is featured in the SHAPE-derived secondary structures of CS1, CS2, and CS4, but the shorter stem is not featured in the structure of CS5, although the protection pattern for the residues involved in the formation of the shorter stem (composed of 5′-CUCA-3′ and 3′-GAGU-5), may indicate base-pairing interactions.

We are less certain about the presence of stem P2 composed of 5′-GGACAGCG-3′ and 3′-CCUGUCGC-5′ regions. Both these regions were part of the constant domain of the RNA library, designed to form an 8-bp stem, unlike 3′-GGUGAGU-5′ in putative stem P1, which emerged as a result of selection. Therefore, the mere presence of potentially base-pairing 5′-GGACAGCG-3′ and 3′-CCUGUCGC-5′ regions does not imply that they form a stem, as it is quite possible that these residues are utilized to create other structural features of the ribozymes. In fact, high reactivity of certain residues within the 3′-CCUGUCGC-5′ region in CS1 and CS4 (See Extended Data Figs. 12 and 13) points to the contrary.

The reviewer’s point that the similar role of the ribozyme 3′-end (the ‘primer’ sequence, see Fig. 1) in binding the template for all four ligases is yet another indication of a common structure is interesting, but difficult to confirm as CS5 clearly differs structurally from CS1, CS2, and CS4, but uses its 3′-end to bind the template, like the other ribozymes. Further prediction is complicated by the lack of reactivity data for a significant portion of this region (Extended Data Figs. 12, 13; residues with no associated SHAPE data are shown in gray).

Considering these observations, we now present TurboFold predicted ‘common’ secondary structures for all four ribozymes (Extended Data Fig. 14). TurboFold is an iterative probabilistic RNA secondary structure prediction algorithm for estimating common secondary structures for multiple sequences, even when they have substantial divergence. While this yielded a common secondary structure for CS1, CS2, and CS4, the predicted CS5

structure that was the closest to the rest still diverged from them significantly, as expected from sequence comparative analysis (Extended Data Fig 12). While TurboFold predicted the presence of stem P1, it did not predict stem P2, which is consistent with the SHAPE-derived structures.

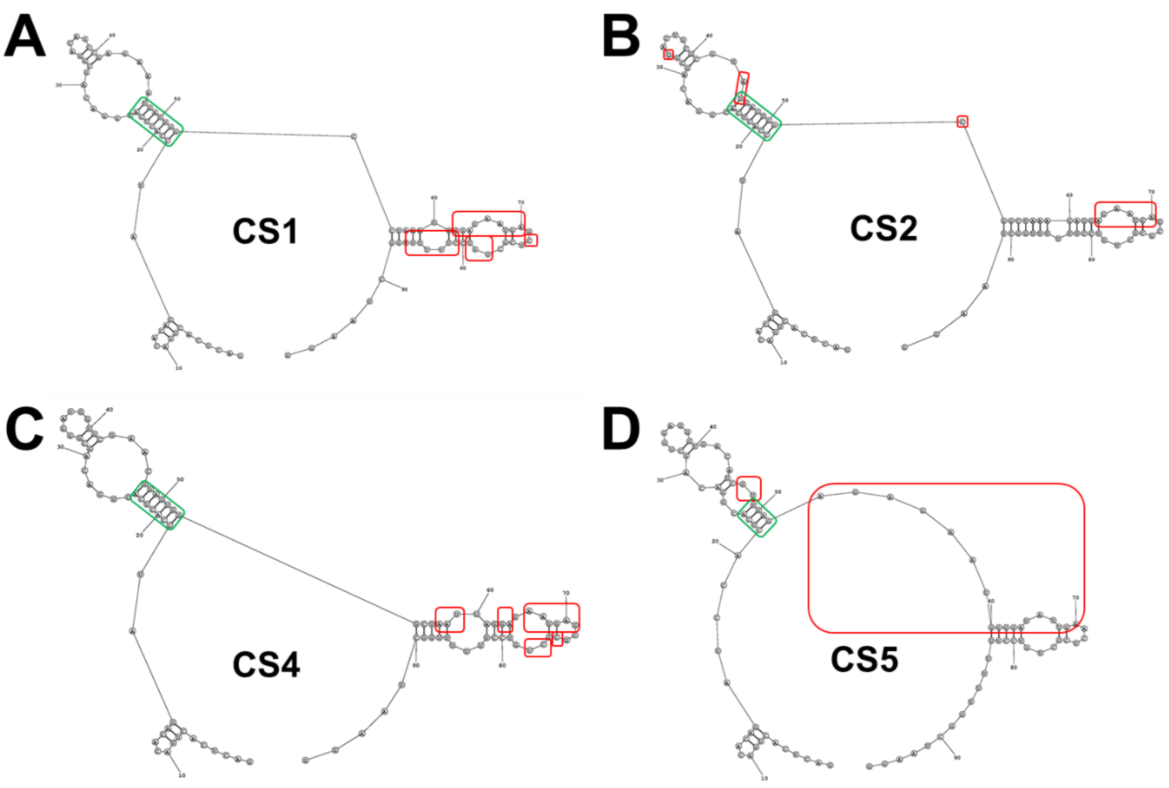

Fig. R2. Computationally-predicted common secondary structures of CS1, CS2, CS4, and CS5. Secondary structures of (A) CS1, (B) CS2, (C) CS4, and (D) CS5 determined by the TurboFold algorithm within the RNAstructure program.[25, 50] SHAPE-reactive regions (normalized reactivity of >0.4) in each RNA are highlighted in red boxes (See Extended Data Figs. 12 and 13). The base-paired stems composed of 5′-CCACUCA-3′ and 3′-GGUGAGU-5′ regions common to CS1, CS2, and CS4 in TurboFold-predicted and SHAPE-derived structures are highlighted in green boxes. A shorter version of this stem composed of 5′-CUCA-3′ and 3′-GAGU-5′ is featured in the TurboFold-predicted structure (but not in the SHAPE-derived structure) of CS5 is also highlighted in a green box. The predicted structures of CS1, CS2, and CS4 converge into an overall common fold, whereas CS5 appears to assume a different fold.

Finally, we caveat this discussion by considering the possibility of local misfolding during SHAPE probing as this experiment was not performed in the presence of the substrate or template to avoid complications arising from simultaneous SHAPE modification of multiple RNAs. In the absence of high-resolution structures, it is difficult to claim structural relationships between these ligases, although there are likely significant similarities due to the observations outlined above.

A closer look at the clustered sequences from extended data Fig 1 could provide strong "phylogenetic" clues for proposing 2D diagrams that might illustrate better the closeness of the four ribozymes clusters.
As an example, by looking at extended Fig 1A, the sequence data of CS1 likely corroborates a covariation between position 9 (A42 in CS1 extended data Fig 12) and position 40 (U73 in

CS1 extended data Fig 12). It looks like that some CS1 sequences have a C at position 9 when it is a G at position 40. Moreover, when position G10 changes into C, position C39 changes into G... This would validate the existence of (at least) the WC bp doublet between A9G10 and C39U40. Interestingly, in CS2 and CS4, the equivalent positions are C8G9/C39G40 and C9G10/C39G40, respectively. In the cluster of CS4 in Extended Fig 1D, it seems that C9G10/C39G40 could also be changed into A9G10/C39U40 and/or C9U10/A39G40. The presence of this tertiary bp doublet validated by sequence comparative analysis in CS1, CS2 and CS4 strongly suggests that they fold into similar secondary and tertiary structures for their catalytic core.

This observation of potential covariation in the ribozyme sequences is interesting. Although the isolated ribozyme sequences mostly consist of highly conserved residues as is clear from Extended Data Fig. 1, the reviewer picked up on some possible covariations.
For example, covariation analysis suggests nucleotides 42 and 73 (9 and 40 in Extended Data Fig. 1) and 43 and 72 (10 and 39 in Extended Data Fig. 1) of CS1 may form base-pairs: A42-U73 or C42-G73, and G43-C72 or C43-G72. Both nucleotides, 42 and 73 are SHAPE-unreactive, which could indicate the presence of the A42-U73 pair, although both residues are unpaired in the SHAPE-derived structure of CS1. However, the G43-C72 pair the reviewer proposed may not be real as C72 exhibits SHAPE reactivity (Extended Data Figs. 12, 13).
The covariation suggested by the reviewer in positions 41/73 and 42/72 (8/40 and 9/39 in Extended Data Fig. 1, respectively) of CS2 is not entirely apparent from Extended Data Figs. 1, where all positions are highly conserved. Similarly, although covariation in positions 42 (9)/73 (40) may suggest a base pair, high SHAPE reactivity of G73 suggest flexibility.
Considering the weak covariation observed from comparative sequence analysis and some contradictory results from experimental secondary structure probing, we decided not to include a discussion on covariation in this manuscript.

The fact that the template is still useful for optimal activity of the CS ribozymes would suggest that their 3' region is still complementary to the template and not used to form base pairs with the rest of the RNA sequence. This would disqualify any of the proposed 3 to 4 bps stems involving the template binding site. As a matter of fact, any proposed internal stems shown in the 2D structures from extended Figure 12 that involve the template binding site are either not there or due to misfolding... Note that the SHAPE reactivity observed in CS1 at the 3' end template binding region could also be due to misfolding as the template was not present... Likewise in CS2, the surrounding of box 2 might look more protected than in CS1, CS4 and CS5 but this could be artifactual as it might result from an alternative fold resulting from the absence of the template (and substrate). In all cases, the SHAPE reactivity in the 3' end region of all CS constructs does not mean much because of the template is lacking in SHAPE experiments.

As the reviewer rightly pointed out elsewhere, SHAPE probing was performed without the template; therefore, the accurate secondary structure of the 3′ end may not be captured in this experiment. We would like to clarify that any secondary structure involving the 3′ end residues is purely a result of computational prediction as we do not have SHAPE data for those positions (hence, shown in gray in Extended Data Figs. 12, 13). Therefore, the last eight nucleotides in the primer (5′-GGCUAAGG-3′) may very well be unpaired, and primed to bind the template during catalysis, as the reviewer suggests.
The SHAPE reactivity in CS1 the reviewer is referring to here is likely in residues 77-79 (5′-UGU-3′), which was not proposed to bind to the template.

Another clue suggesting that they have similar fold is that the box 3 region is highly modified by SHAPE in all CS constructs. Moreover, box 2 is similarly accessible in CS1, CS4 and CS5. Box 2 and 3 are highly conserved in CS1, CS2 and CS4 and this region is quite intriguing. It might adopt an unusual RNA fold (a G-quartet???), or could be involved in the recognition of the substrate and/or involved in the formation of the catalytic site.

We thank the reviewer for pointing this out. We have included this point in our revised manuscript:
"We also noticed high SHAPE reactivities in the 5'-AAUGA-3' region (residues 66-70) in all four ribozymes, indicating that this region is unpaired, further pointing to the possibility of a shared structure."
Box 2 and Box 3 regions are G-rich, but they contain 5-6 Gs, whereas the minimum number of Gs required for the simplest two-tiered G-quadruplex is 8 (2 G-quartets). Therefore, we do not think this region forms a G-quadruplex. A single G-quartet is not a stable structure.

In any case, a similar catalytic core for CS1, CS2, CS4 and CS5 is supported by the facts that:
(1) CS1, CS2, CS4 and CS5 have the same activity,
(2) CS1, CS2, CS4 and CS5 seem to have the same type of sequence reliance with the template, albeit the template is dispensable for the best class of ribozymes (CS1)
(3) Comparative sequence analysis warrants the presence of similar pseudo-knots with similar conserved regions in all CS constructs and demonstrate the presence of a covariation between position x and y for all CS constructs.
(4) The new SHAPE data is best explained by assuming that the CS constructs adopt a similar structure.
In conclusion, the authors should highlight these findings relative to the 2D/3D structure of the CS ribozymes and modify their article accordingly.

As mentioned above, we have taken these findings into account and summarized them in a new section in our revised manuscript.

Finally, as the authors mentioned that they have a preliminary X-ray structure of the CS1 ribozyme, nothing should prevent them to take advantage of some of the preliminary data for showing that the CS2, CS4 and eventually CS5 families are likely to fold into the same active 2D/3D structures as the one of CS1 unless their CS1 ribozyme atomic structure does not correspond to the native active fold...

Unfortunately, the crystal structure appears to be of a misfolded ribozyme, which is why we did not include any of that data here.

```
RS1 ACUCACUGACACAGAUCCACUCACGGACAGCG-GAAUGCUGCCA--ACCGUGCG-GG--CUAAUUG-GCAGACUGAGCUCGCUGUCCUUUUUUGGCUAAGG 13 from CS1

                                                    Box 1    Box 2      Box 3
                    P1(5')  P2(5')         x        P1(3')                   ****  y P2(3')
                    (((((((.(((((((( ........((.....)))))) ).  ....... ..........))))))))))
                    (((((((.((((((([[ [....]]]((.....)))))) ).  ....... ..........))..))))))
                    (((((((.(((((((. .......(((.....)))))) ).  ....... ..........))))))))))
                    (((((((.((((((.. ......((((.....)))))) ).  ....... ..........))))))))))
                    (((((((.(((((... ....((.(((.....)))))) ).  ....... ..........))))))))))
CS1 ACUCACUGACACAGAUCCACUCACGGACAGCG-GACAGCCGAGA--AAUGAGUG-GC--CUAAAUG-GGAGAAUGAGCUCGCUGUCCUUUUUUGGCUAAGG ref
CS2 ACUCACUGACACAGAUCCACUCACGGACAGCG-GACUGC-GCGU--A-UGAGUG-GCGGCUAAA-GAGGAGAAUGAGCGCGCUGUCCUUUUUUGGCUAAGG 10 from CS1
CS4 ACUCACUGACACAGAUCCACUCACGGACAGCGGGAUGGU-GCGA--ACUGAGUG-GG--CUAAUUA-GGAGAAUGAGCGCGCUGUCCUUUUUUGGCUAAGG 11 from CS1
                    ((.((((.(((((((. .......((......)))).).)     ..... ...........))))))))
CS5 ACUCACUGACACAGAUCCACUCACGGACAGCGGGAGGGU-GACAUCGUUGAGAGAG-----AA-UGGGGAUAUUGAACUCGCUGUCCUUUUUUGGCUAAGG 20 from CS1
```

Figure: Alignment for CS1, CS2, CS4 and CS5 taking into consideration indels.

Conserved box regions are shaded in grey. The nts indicated by * in box 3 are those highly reactive to SHAPE in CS1 CS2, CS4 and CS5 ribozymes. Positions x and y are those found covarying... Brackets indicate possible 2D pairing. Note that the pairing formed around x and y and proven by covariation could be between 2 and 5 bps and can somewhat compete with the formation of P2 if more than 2 bps.
